# Controlling for cellular heterogeneity using single-cell deconvolution of gene expression reveals novel markers of colorectal tumors exhibiting microsatellite instability

**DOI:** 10.18632/oncotarget.27935

**Published:** 2021-04-13

**Authors:** Matthew A.M. Devall, Graham Casey

**Affiliations:** ^1^Center for Public Health Genomics, Department of Public Health Sciences, University of Virginia, Charlottesville, VA, USA

**Keywords:** colorectal cancer, single-cell deconvolution, microsatellite instability, RNA-sequencing, enteroendocrine

## Abstract

Approximately 15% of colorectal cancer (CRC) cases present with high levels of microsatellite instability (MSI-H). Bulk RNA-sequencing approaches have been employed to elucidate transcriptional differences between MSI-H and microsatellite stable (MSS) CRC tumors. These approaches are frequently confounded by the complex cellular heterogeneity of tumors. We performed single-cell deconvolution of bulk RNA-sequencing on The Cancer Genome Atlas colon adenocarcinoma (TCGA-COAD) dataset. Cell composition within each dataset was estimated using CIBERSORTx. Cell composition differences were analyzed using linear regression. Significant differences in abundance were observed for 13 of 19 cell types between MSI-H and MSS/MSI-L tumors in TCGA-COAD. This included a novel finding of increased enteroendocrine (*q* = 3.71E^-06^) and reduced colonocyte populations (*q* = 2.21E^-03^) in MSI-H versus MSS/MSI-L tumors. We were able to validate some of these differences in an independent biopsy dataset. By incorporating cell composition into our regression model, we identified 3,193 differentially expressed genes (*q* = 0.05), of which 556 were deemed novel. We subsequently validated many of these genes in an independent dataset of colon cancer cell lines. In summary, we show that some of the challenges associated with cellular heterogeneity can be overcome using single-cell deconvolution, and through our analysis we highlight several novel gene targets for further investigation.

## INTRODUCTION

Colorectal cancer (CRC) is a complex, heterogenous disease. At least two broad molecular pathways contribute to the development of CRC tumors: microsatellite instability (MSI) and chromosomal instability (CIN). Microsatellite instability-high (MSI-H) tumors account for ~15% of CRC tumors, and are driven by a dysregulation of mismatch repair (MMR) [1]. The majority of MSI-H tumors (80%) occur via acquired epigenetic silencing of the MMR gene *MLH1*. In contrast, microsatellite stable (MSS) tumors account for the majority (~85%) of CRC tumors, and are defined by increased loss or gain and rearrangement of chromosomes (CIN phenotype) [2]. MSI-H and MSS tumors have been shown to differ with regards to survival [3] and response to treatment [4], but the molecular mechanisms driving the differences between these tumor types remain poorly understood. One commonly employed approach to interrogate differences between tumor types is through a comparative analysis of RNA-sequencing (RNA-seq) data [5]. However, the cellular heterogeneity of tumors can mask important gene expression differences identified by RNA-seq. To improve understanding of the molecular mechanisms across tumor subtypes, cellular composition must be adequately controlled.

Various methodological advances have been made to address the problems arising from tumor cellular heterogeneity including the sorting of cell populations prior to RNA-seq and the development of single-cell RNA-seq (scRNA-seq) approaches. However, these studies are often limited by factors such as availability of sufficient material or high cost associated with scRNA-seq. While these methods increase resolution, they are often hindered by small study designs, which reduces generalizability. The application of single-cell deconvolution approaches using bulk RNA-seq data therefore provides an opportunity to infer cell composition differences of large tumor datasets at reduced cost [6]. Indeed, our group has previously employed single-cell deconvolution to quantify and account for variation in cell composition in a large colon organoid study [7].

In this study, we aim to identify differences in cell composition between MSI-H and MSS/microsatellite instability-low (MSI-L) tumors in The Cancer Genome Atlas Colon Adenocarcinoma (TCGA-COAD) dataset [8]. We achieve this by using a machine learning approach [6] and by incorporating scRNA-seq data derived from normal colon biopsies [9] to deconvolute bulk RNA-seq data [8]. We estimate cell type abundance and identify novel cellular composition differences between MSI-H and MSS/MSI-L tumors, which we then validate in an independent cohort of CRC tumors [10]. Following adjustment for cell composition, we identified novel differentially expressed genes (DEG)s between MSI-H and MSS/MSI-L tumors in TCGA-COAD and replicate many of these DEGs in an independent analysis of colon cancer cell lines derived from Cancer Cell Line Encyclopedia (CCLE) [11]. Together, we provide data showing that single-cell deconvolution analysis of tumors can be used to address cellular heterogeneity, and has the potential to reveal novel insight into tumor biology.

## RESULTS

### Differential expression of genes specific to immune cell types are commonly overexpressed in MSI-H tumors

MSS and MSI-L tumors were merged for comparisons to MSI-H tumors in all downstream analysis based on the similarities of expression profiles observed between MSI-L and MSS tumors (Supplementary Figure 1A). Further, a total of 89 significant DEGs (*q* = 0.05) were identified in our preliminary regression analysis of MSI-L vs MSS tumors. This was in stark contrast to the extent of transcriptomic variation observed between MSI-H versus MSI-L (5,472 DEGs) or MSI-H versus MSS tumors (8,641 DEGs), where 61.48- and 97.01-fold more DEGs were reported than in MSI-L vs MSS tumors, respectively (Supplementary Figure 1B). This is in line with clinical practice, where MSI-L tumors are often considered to be similar to MSS tumors [12]. This grouping has also been used in other studies [13].

**Figure 1:**
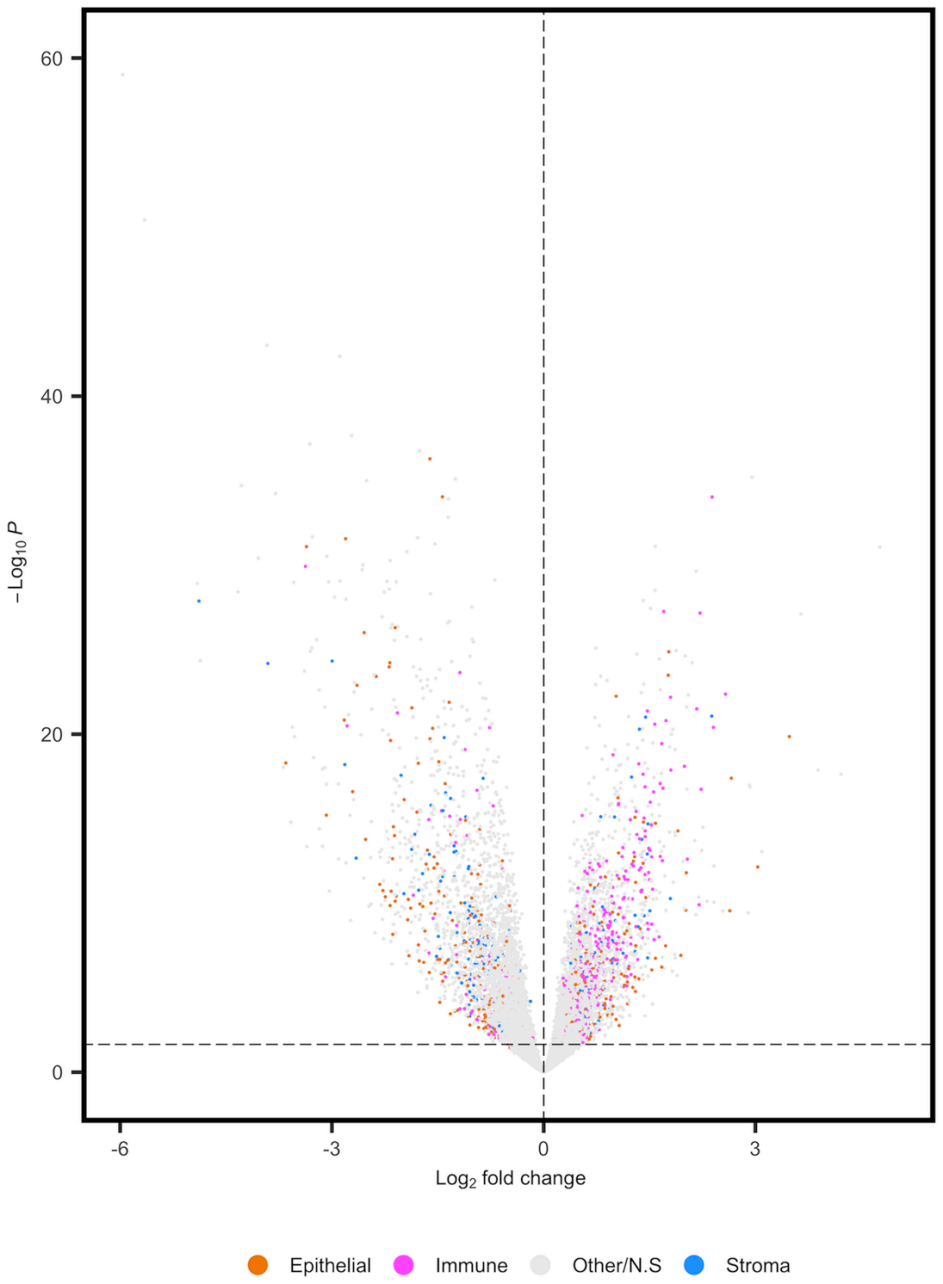
Differential expression analysis of TCGA-COAD prior to adjustment for cell composition. Volcano plot displaying direction of effect for bulk cell types. Positive log2fold changes correspond to increased expression in MSI-H tumors.

Differential expression analysis of RNA-seq data from MSI-H versus MSS/MSI-L tumors identified 8,693 FDR corrected DEGs (*q* = 0.05). Our preliminary analysis aimed to determine the potential impact of cell composition on the DEGs reported in an RNA-seq analysis of MSI-H versus MSS/MSI-L tumors. We found that 17.92% (1,558) of these DEGs were potential markers of specific cell types [9]. In total, 72.62% (515/656) of significant DEGs corresponding to immune cell markers were expressed at higher levels in MSI-H versus MSS/MSI-L tumors (Figure 1). This finding is in line with reports that MSI-H tumors are most frequently associated with increased immune cell populations [14]. We extended this analysis by increasing the resolution of the cell types considered (Supplementary Table 1). We found that expression markers of both transit amplifying (TA) and CD8^+^T cell populations were consistently higher in MSI-H versus MSS/MSI-L tumors. Further, significant reductions in expression were identified for 41 of 50 FDR corrected stem cell-related genes in MSI-H tumors, including *LGR5* (*q* = 2.67E^-04^). Significant reductions in expression were also identified for 101 of 146 FDR corrected colonocyte expression markers in MSI-H tumors. Thus, many of the significant differences identified in standard regression analysis of tumor biopsies are reflective of variation in cell composition across tumors.

**Figure 2:**
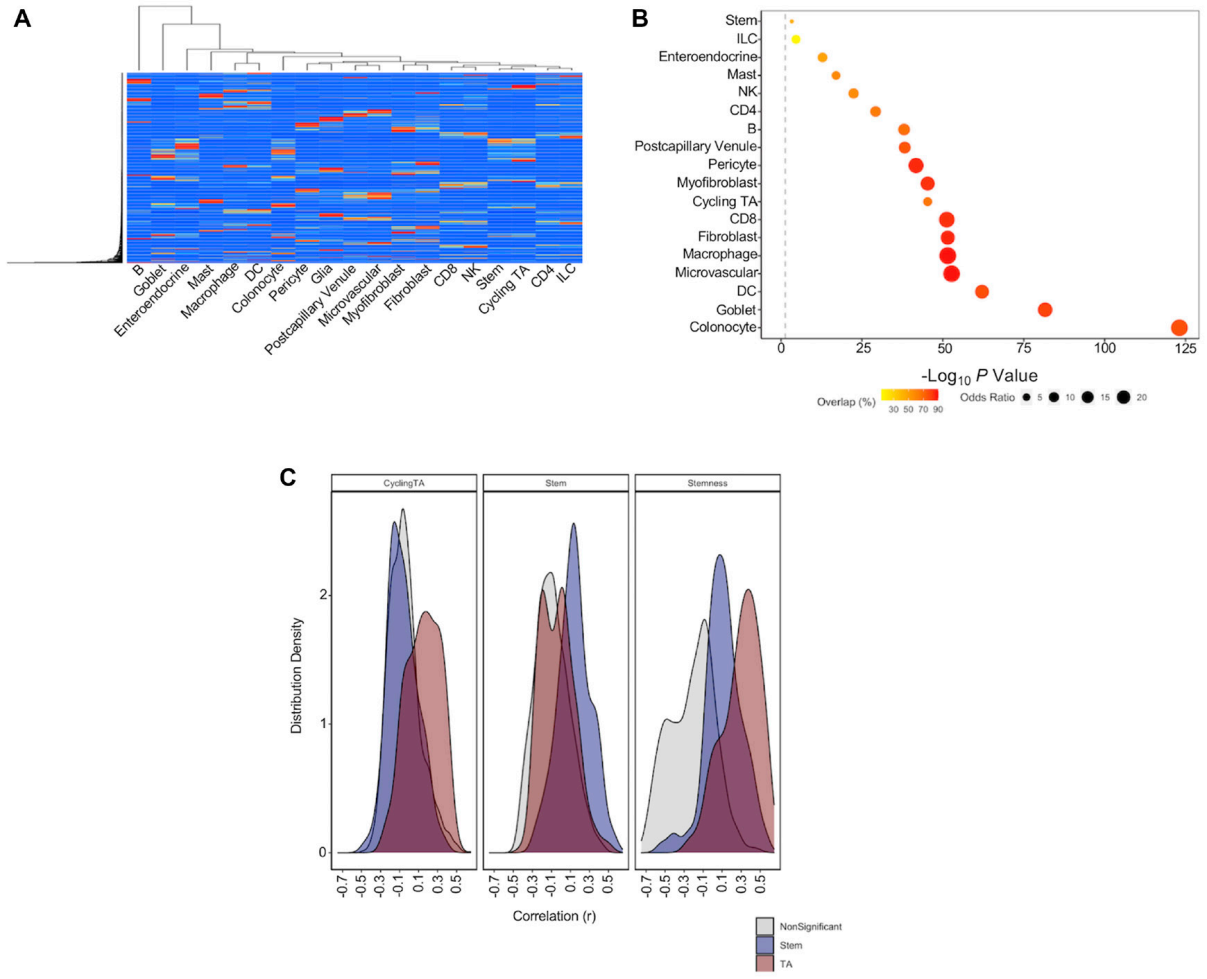
Single cell deconvolution of bulk RNA-seq datasets. (**A**) Heatmap to show separation of scRNA-seq cell populations based upon expression of signature genes. (**B**) Summary of enrichment analysis (one-way Fisher’s exact test) for cell type markers in differential expression analysis of cell scores. Grey line represents log_10_(0.05). Percentage overlap reflects percentage of cell type markers for a given cell type that were significant within regression of cell score. (**C**) Cell scores for cycling TA and stem cells generated using this approach, and a marker of stemness generated in a previous study were correlated to expression markers of TA cells (red), stem cells (blue) and significant markers of other colon cell types (grey).

To address the challenge of cellular heterogeneity and to accurately capture cell composition of TCGA-COAD tumors, we employed single-cell deconvolution using publicly available scRNA-seq data generated from normal colon biopsies [9]. A signature matrix was generated from scRNA-seq data, which stratified cell populations based upon the average gene expression of select genes (defined by CIBERSORTx) across the 19 cell types considered (Figure 2A). Cell scores were then generated for these cell types using this signature matrix [6]. Regression analysis was performed on each cell score to determine whether cell scores capture known expression markers of relevant cell types. One-way Fisher’s exact tests determined significant enrichments for known expression markers in the DEGs identified in these cell score regressions (Figure 2B) [9]. Glial cell gene expression markers were not provided within the scRNA-seq dataset. As such, we identified canonical gene expression markers for glial cells using an online database [15]. Of the eight canonical markers of enteric glial cells identified, *ALDH1A3* (*q* = 1.49E^-25^), *SLC18A2* (*q* = 3.39E^-11^), *S100B* (*q* = 9.69E^-11^), *FOXD3* (*q* = 5.98E^-10^), *SLC18A2* (*q* = 3.39E^-11^), *GFAP* (*q* = 3.58E^-09^) and *GFRA3* (*q* = 1.24E^-04^) were significantly upregulated (*q* = 0.05) in glial cell regressions of TCGA-COAD tumors. These findings highlight that the deconvolution approach used here accurately captures the expected expression of relevant cell types.

We used immunedeconv [16] to generate stromal and immune cell scores in matched tumor samples using four additional deconvolution methods [17–20]. We were able to estimate the relative performance of each method by correlating cell type expression markers to cell scores generated from each approach. We considered performance as the difference (shift) in median correlation between cell score and expression markers of that cell type, and the median correlation of the same cell score to expression markers of other cell types. The performance of our cell scores was comparable to other approaches (Supplementary Table 2).

Finally, we compared the correlation of CRC stem cell markers *LGR5*, *CD24* and *EPCAM* to both a previously generated stemness score [21] and to the stem cell scores generated in our analysis. We found that *LGR5*, *CD24* and *EPCAM* were more positively correlated with stem cell scores generated in our method (*r* = 0.26; *r* = 0.45; *r* = 0.38 respectively) than with the stemness score (*r* = 0.08; *r* = 0.14; *r* = 0.30 respectively). We extended this approach to determine the relative ability of stemness scores, stem cell scores and cycling TA cell scores to capture the expression of markers of normal colon stem cells and TA cells [9] (Figure 2C). We find that stem cell scores generated here are better able to distinguish TA and stem cell gene maker expression than markers of stemness. These results are perhaps unsurprising given that the stemness score was originally designed as a pan cancer score of global dedifferentiation, rather than a colon-specific marker of stem cell content.

### Defining a high-resolution cellular roadmap of CRC tumors

**Figure 3:**
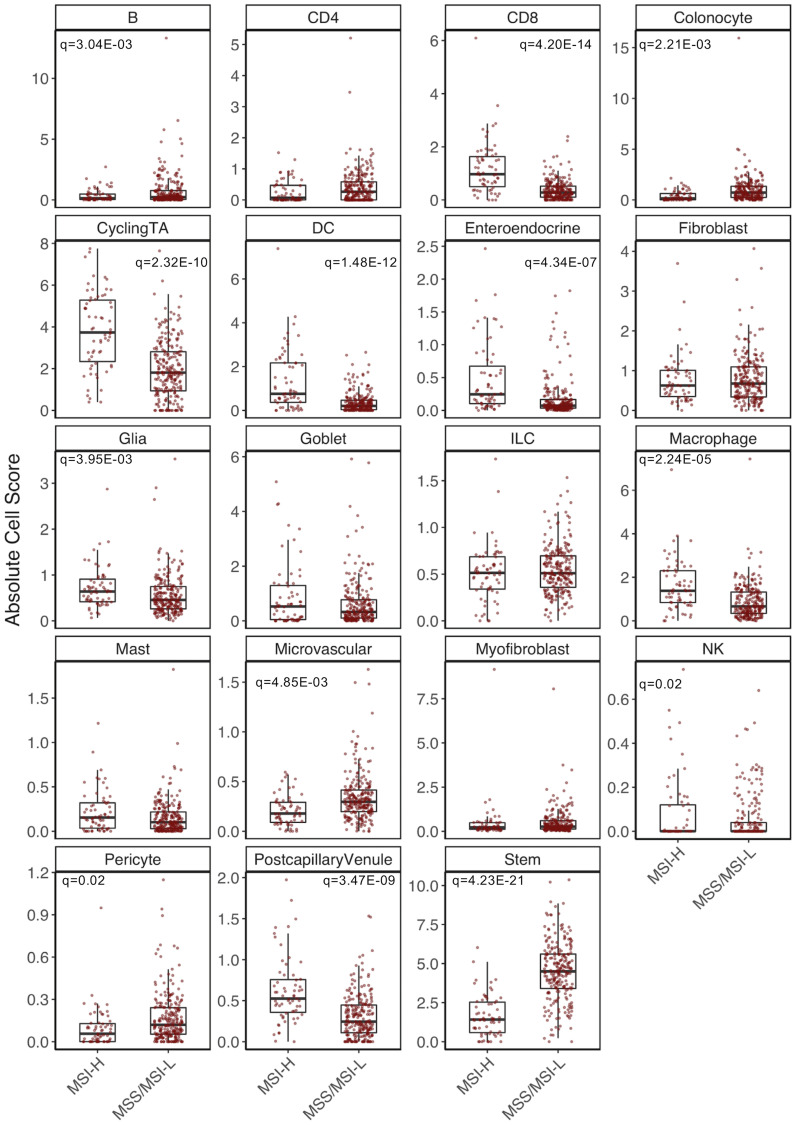
Cell composition analysis of TCGA-COAD dataset. Significant differences determined through linear regression analysis while adjusting for colon location, sex, consensus purity estimate and tumor stage.

We first aimed to determine differences in overall cell composition between MSI-H and MSS/MSI-L tumors. A linear regression was performed on MSI status for each cell score. Significant differences in 13 of 19 cell populations were identified (*q* = 0.05) (Figure 3). Increased cell abundance was observed for six of eight immune cell populations in MSI-H tumors, in line with increased immune cell content associated MSI-H tumors [14]. A cytolytic signature was generated for each sample by averaging the expression of six genes (*GZMA*, *GZMB*, *GZMH*, *GZMK*, *GZMM* and *PRF1*), as demonstrated in Rooney et al. [22]. This signature was strongly correlated to CD8^+^T cell content (*r* = 0.76). Further, MSI-H CD8^+^T cells were found to have a significantly increased cytolytic score (*P* = 1.53E^-53^), indicating an increased potential for tumor immune cell killing in MSI-H samples. We also observed an increase in enteroendocrine cell (EEC) content (*q* = 3.71E^-06^), in MSI-H versus MSS/MSI-L tumors. To the best of our knowledge, this analysis represents the first to report this finding. We also observed a decrease in colonocyte (*q* = 2.21E^-03^) and stem cell content (*q* = 4.23E^-21^) as well as an increase in the cycling TA cell population (*q* = 2.32E^-10^) in MSI-H tumors, highlighting the importance of considering cellular heterogeneity of epithelial cells in these analyses.

We were able to validate some of these changes in cellular composition in a second, smaller cohort of MSI-H tumors (GSE146889) [10]. We were unable to capture EEC, dendritic cell or innate lymphoid cell gene signatures (Supplementary Figure 2). As a result, no analysis was performed on these cell types in this dataset. We replicated reduced stem cell (*P* = 0.02) as well as increased cycling TA (*P* = 7.05E^-03^) and macrophage cell content (*P* = 0.029) in MSI-H versus MSS tumors. Further, we also observed trends for a significant increase in CD8^+^T cells (*P* = 0.076) and a reduction in colonocytes (*P* = 0.08) in MSI-H versus MSS tumors (Supplementary Figure 3).

For sensitivity, we repeated our analysis of TCGA-COAD by stratifying MSS and MSI-L. Here, we found that all 13 significant cell populations remained significant (*q* = 0.05) in a regression of MSI-H versus MSS, while no significant differences were observed between MSS and MSI-L tumors. Of the 13 significantly different cell populations identified between MSI-H and MSS tumors, seven were also found to be significantly different between MSI-H and MSI-L tumors (Table 1). Replication of three additional cell types was confirmed at a nominal significance threshold (*P* = 0.05). These data further support a strong similarity between MSS and MSI-L tumors.

**Table 1:** Cell composition analysis of MSI-H versus MSS and MSI-L versus MSS tumors in TCGA-COAD

Cell-Type	MSI-H vs MSS (*n* = 242)			MSI-H vs MSI-L (*n* = 116)			MSI-L vs MSS (*n* = 231)			Significant in MSI-H vs MSS/MSI-L
	T-Statistic	*P*	FDR	T-Statistic	*P*	FDR	T-Statistic	*P*	FDR	
B	**-3.29**	**1.14E^-03^**	**2.16E^-03^**	-2.19	0.03	0.08	-0.73	0.47	0.68	True
CD4^+^T	-2.08	0.04	0.053	-1.29	0.20	0.27	0.88	0.38	0.68	False
CD8^+^T	**7.87**	**1.37E^-13^**	**1.30E^-12^**	**3.59**	**5.09E^-04^**	**2.41E^-03^**	1.05	0.30	0.68	True
Colonocytes	**-4.26**	**3.03E^-05^**	**7.20E^-05^**	**-2.68**	**8.58E^-03^**	**0.02**	1.39	0.17	0.53	True
CyclingTA	**6.57**	**3.26E^-10^**	**1.55E^-09^**	**4.29**	**3.94E^-05^**	**3.74E^-04^**	0.82	0.41	0.68	True
DC	**7.11**	**1.40E^-11^**	**8.87E^-11^**	**3.65**	**4.14E^-04^**	**2.42E^-03^**	1.52	0.13	0.53	True
Enteroendocrine	**4.89**	**1.92E^-06^**	**6.08E^-06^**	**2.72**	**7.63E^-03^**	**0.02**	-0.18	0.86	0.92	True
Fibroblast	-0.19	0.85	0.90	0.17	0.91	0.96	-0.89	0.37	0.68	False
Glia	**3.07**	**2.34E^-03^**	**3.71E^-03^**	1.28	0.20	0.27	-0.50	0.62	0.84	True
Goblet	1.92	0.06	0.08	0.46	0.65	0.82	1.85	0.07	0.53	False
ILC	-0.31	0.76	0.85	0.31	0.76	0.88	-0.73	0.47	0.68	False
Macrophage	**4.63**	**6.05E^-06^**	**1.64E^-05^**	2.00	0.048	0.09	-0.11	0.92	0.92	True
Mast	1.82	0.07	0.08	1.34	0.18	0.27	0.19	0.85	0.92	False
Microvascular	**-3.66**	**3.13E^-04^**	**6.60E^-04^**	-2.09	0.04	0.08	1.42	0.16	0.53	True
Myofibroblast	0.01	0.99	0.99	0.05	0.96	0.96	-0.86	0.39	0.68	False
NK	**2.44**	**0.02**	**0.02**	1.30	0.20	0.27	0.15	0.88	0.92	True
Pericyte	**-3.23**	**1.42E^-03^**	**2.45E^-03^**	-0.27	0.79	0.88	-1.72	0.09	0.53	True
Postcapillary Venule	**6.21**	**2.49E^-09^**	**9.46E^-09^**	**3.07**	**2.73E^-03^**	**0.01**	-0.20	0.85	0.92	True
Stem	**-10.84**	**2.03E^-22^**	**3.86E^-21^**	**-6.52**	**2.41E^-09^**	**4.58E^-08^**	-1.60	0.11	0.53	True

Positive test statistic indicates increased cell content in tumors with greater MSI. FDR corrections were calculated for each regression analysis individually and FDR significance was set at 5%. Bond fold indicates cell-types that pass FDR correction for that regression analysis.

### Differential expression following adjustment for cell composition

**Figure 4:**
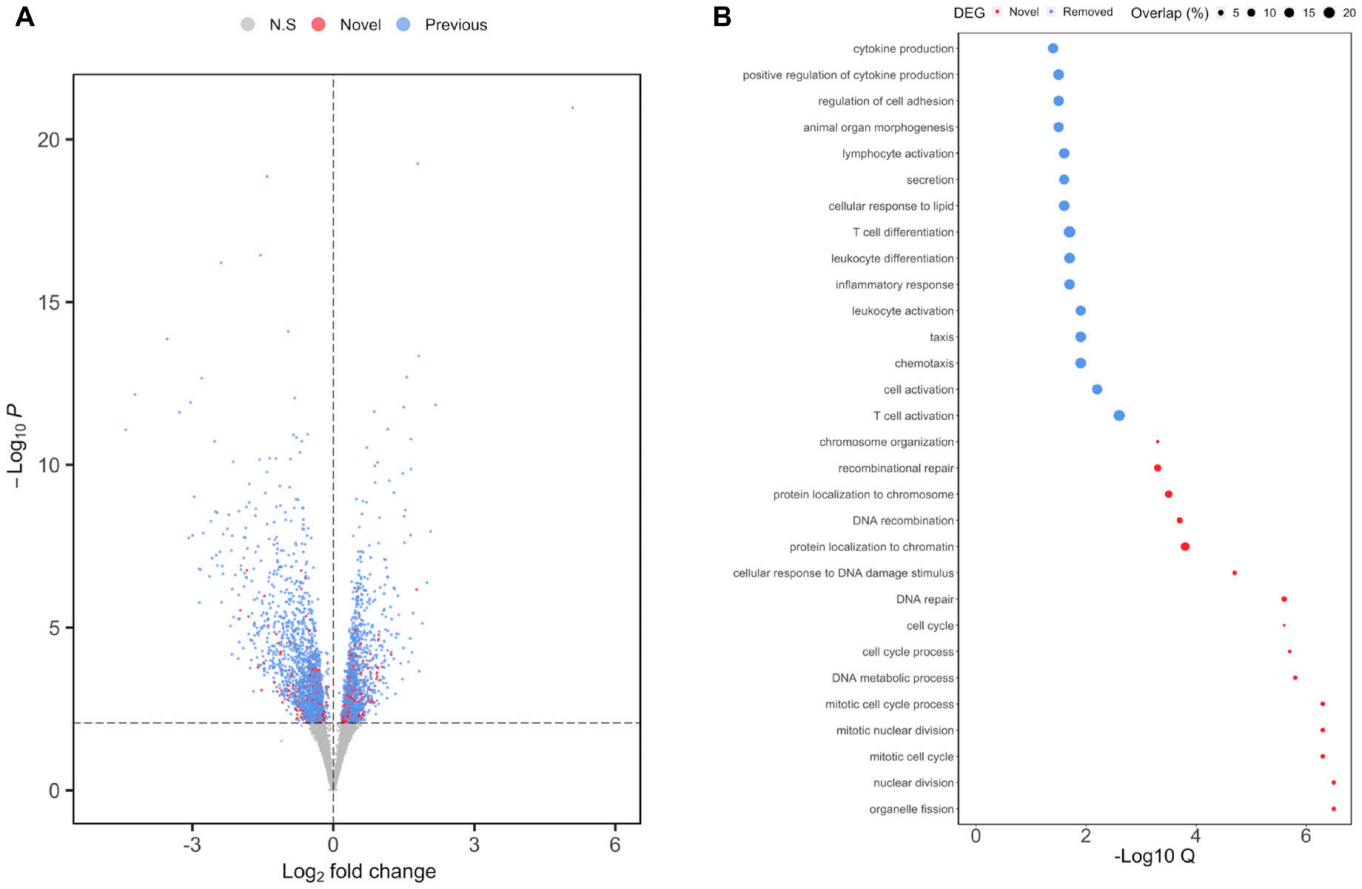
Differential expression analysis of MSI status in TCGA-COAD following adjustment for cell composition. (**A**) Volcano plot of results from regression on MSI status in TCGA-COAD cohort. Novel significant DEGs (*q* = 0.05) are highlighted in red, whereas DEGs identified also identified prior to adjustment for cell composition are highlighted in blue (**B**) Pathway analysis of DEGs found to be significantly reduced in MSI-H versus MSS/MSI-L tumors. Color reflects pathway analysis performed on either novel (red) or on DEGs no longer considered to be significant (*q* = 0.05) following adjustment for cell composition. Size of each circle represent the percentage of overlap between the number of DEGs and the total number of genes within a given pathway.

To correct for the effects of cell composition in our analysis of MSI-H versus MSS/MSI-L tumors, we repeated our initial regression while incorporating cell composition scores. We identified 3,193 DEGs (*q* = 0.05), of which 556 were not reported in our original analysis and were thus deemed novel (Figure 4A). Pathway analysis performed on the novel DEGs that displayed reduced expression in MSI-H compared to MSS/MSI-L tumors revealed an enrichment for DNA repair (*q* = 2.81E^-06^). Indeed, 124 Gene Ontology biological processes were enriched in this analysis, of which 15 were associated with DNA damage, mismatch or repair processes (Figure 4B). Interestingly, *PMS1* (*q* = 1.90E^-03^), *MSH2* (*q* = 2.80E^-03^) and *MSH6* (*q* = 0.02) only reached significance following adjustment for cell composition. Inherited mutations of these genes are associated with Lynch syndrome, a genetic condition that greatly increases the risk of MSI-H tumor development [23]. In contrast, pathway analysis of downregulated DEGs that were no longer significant following adjustment for cell composition revealed enrichments for cell-specific processes such as T cell activation (*q* = 2.40E^-03^), leukocyte differentiation (*q* = 0.03) and T cell differentiation (*q* = 0.03). However, a notable absence for enrichment of pathways associated with DNA repair or mismatch repair was observed (Figure 4B) [24, 25]. Together, these findings highlight that adjusting for cell composition, leads to the identification of important pathways, and reduced the reporting of findings that can be attributed to cell-specific variation. A full list of the pathways and DEGs identified within each analysis can be found in (Supplementary File 1).

Given the cellular heterogeneity of tumor biopsies and the relatively small sample size of the GSE146889, we did not attempt to replicate these differences in this cohort. Instead, we performed a similar regression analysis to identify DEGs associated with MSI status in a dataset of colon cancer cell lines [11]. Here we generated cell scores for four epithelial cell populations (Supplementary Figure 4). Regression analysis was then performed on MSI status while adjusting for cell composition. We were able to replicate 607 of these DEGs at nominal significance (*P* = 0.05) and 221 following FDR correction (*q* = 0.05) (Supplementary Table 3). A one-way Fisher’s exact test was performed, which revealed that there was a significant enrichment of overlap between the FDR corrected DEGs identified in these two datasets (Odds ratio = 1.68, *P* = 3.09E^-10^). Thus, our secondary analysis of colon cancer cell lines provides an independent replication of the results identified in TCGA-COAD tumors. With regards to the 556 DEGs identified in TCGA-COAD only after adjustment for cell composition, 56 were also identified in colon cancer cell lines dataset (*P* = 0.05), of which 18 remained significant following FDR correction (Supplementary Table 5). The three most significant novel genes identified in MSI-H vs MSS/MSI-L tumors that were subsequently replicated in colon cancer cell lines were *AGMO, LINC02577* and *KIF1A*, all of which displayed reduced expression in MSI-H cell lines and tumors compared to MSS/MSI-L cell lines and tumors. To the best of our knowledge, roles for these three genes in MSI-H tumors have yet to be defined.

Network analysis for the identification of candidate modules associated with microsatellite instability.

We regressed out the effects of cell composition and additional covariates prior to our network analysis (see Methods) to determine patterns of differential co-expression between MSI-H and MSS/MSI-L tumors. We performed weighted gene co-expression network analysis (WGCNA) [26], which identified a network consisting of 96 modules of coordinated expression (Supplementary Figure 5). Of these modules, 35 were found to be significantly different between MSI-H and MSS/MSI-L tumors following strict Bonferroni correction (*q* = 0.05) (Supplementary File 2, Figure 5). We used an independent method to validate the co-expression observed within the 96 modules identified in our analysis. We uploaded gene lists for each module into STRING [27]. For each module, we calculated enrichment scores for protein-protein interaction (PPI). Of the 97 modules, 87 were enriched for PPI, including 34 of the 35 significant modules identified (Supplementary Table 4). Intramodular analysis was then performed to determine the relationship between a gene’s significance and its module membership. Modules relevant to differences between MSI-H and MSS/MSI-L tumors should contain genes with a high module membership that are also significantly associated with the trait of interest. We found that these two characteristics were significantly positively correlated (*P* = 0.05) in 18 of the 35 modules identified (Supplementary Figure 6). To provide functional characterization, pathway analysis was performed for each of these 18 modules (Supplementary File 2).

**Figure 5:**
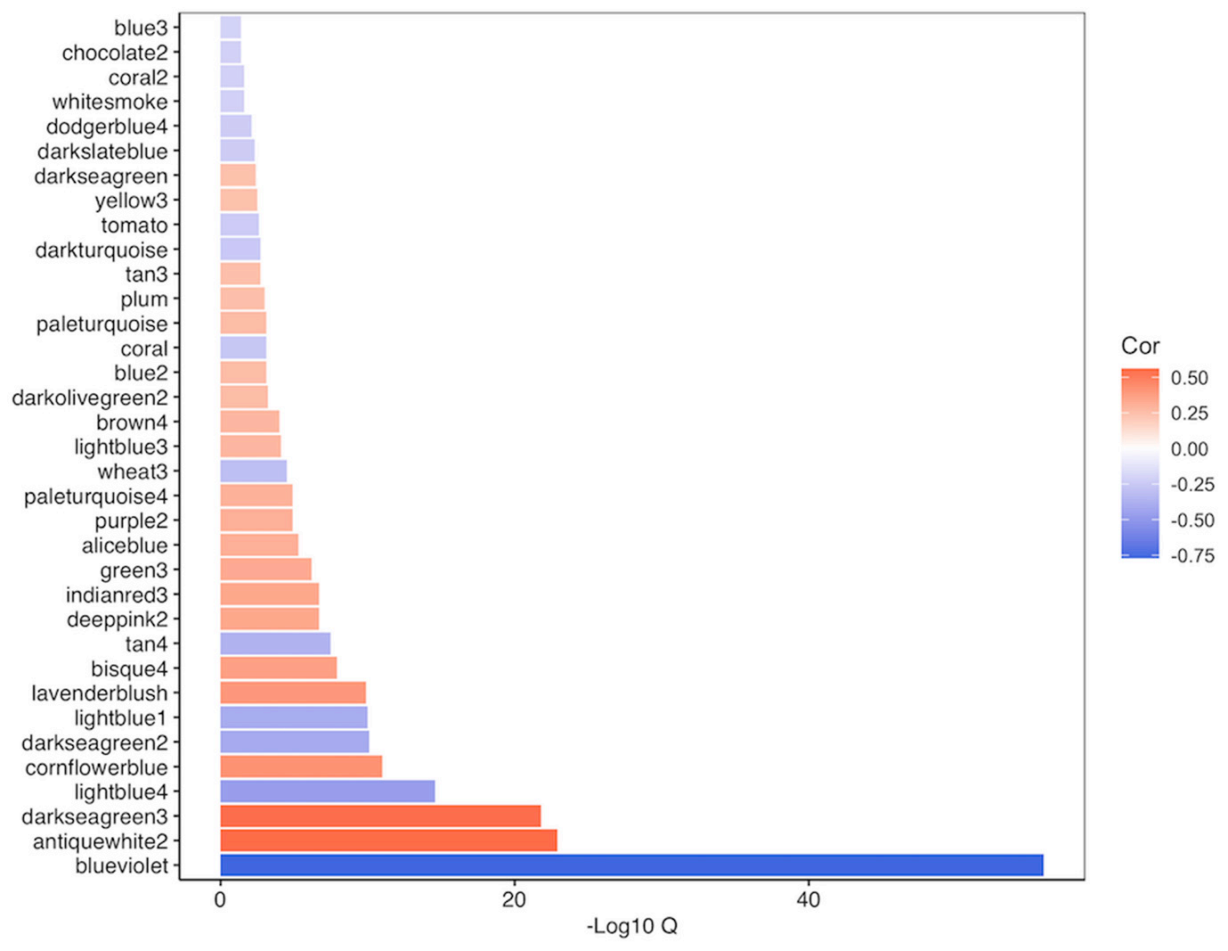
Overview of relationship of significant modules (*q* = 0.05) identified through WGCNA of TCGA-COAD following adjustment for cell composition to MSI status. Negatively correlated modules are indicative of modules primarily consisting of genes that were reduced in MSI-H versus MSS/MSI-L tumors.

**Figure 6:**
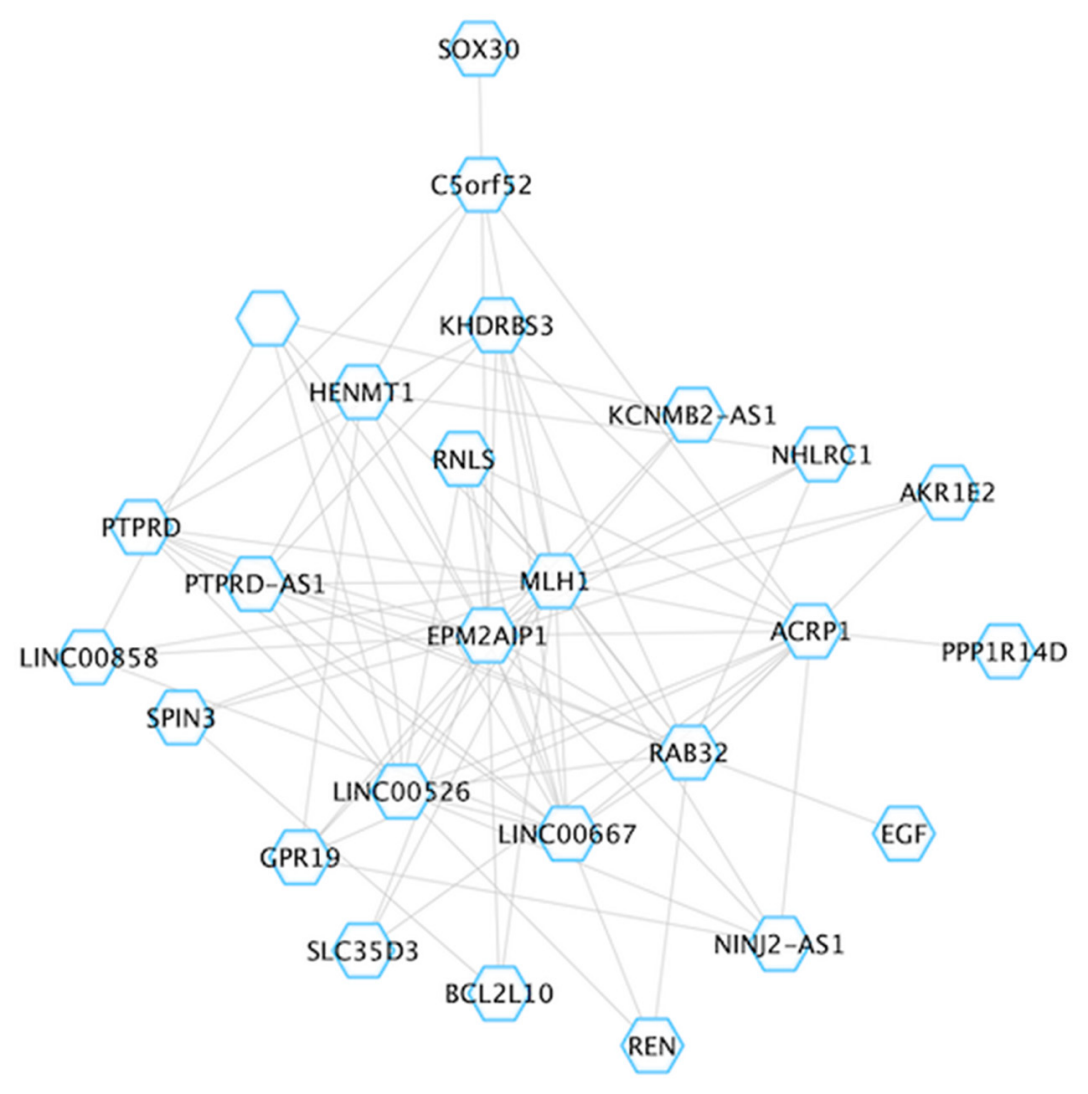
Overview of the blueviolet module. For visualization, the network was imported into Cytoscape. Grey lines reflect edges (connections) between hubs (genes).

**Figure 7:**
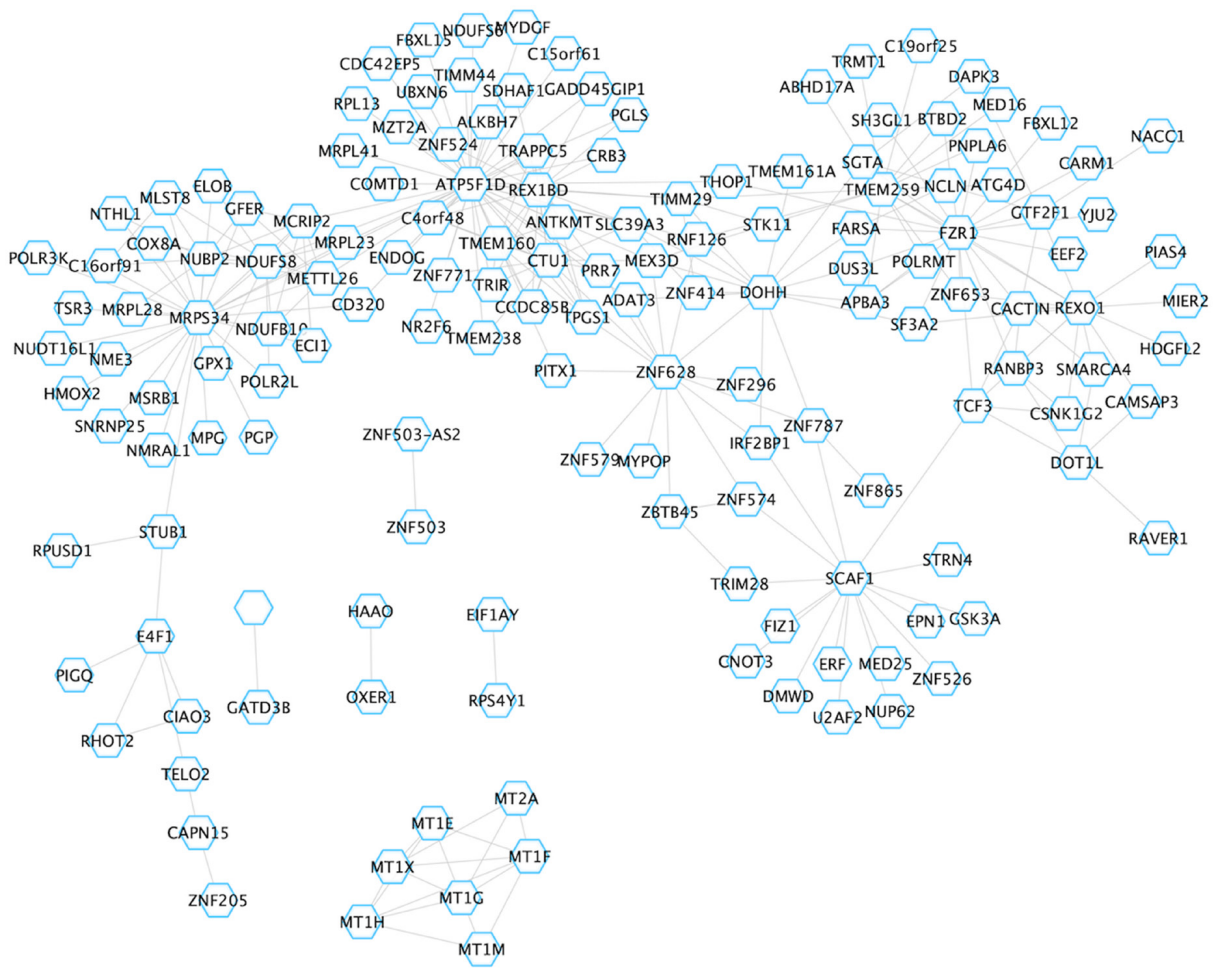
Overview of the brown4 module. For visualization, the network was imported into Cytoscape. Grey lines reflect edges (connections) between hubs (genes).

The blueviolet module was the most significant module identified in our analysis (*q* = 9.07E^-57^), and consisted of 28 genes, including central hubs *MLH1* and *EPM2AIP1* (Figure 6). This module also contained nodes for *RAB32*, *EGF* and *PTPRD*. *RAB32* is a ras proto-oncogene family member that has been previously associated with MSI-H tumors [28, 29], while both *EGF* and *PTPRD* have important roles in the regulation of cell growth and differentiation [30, 31]. Better understanding the relationship between *MLH1* and other genes in this module may provide improved insight into MSI-H tumor biology. Brown4’s module eigengene was significantly positively correlated with MSI-H status (*q* = 5.10E^-06^), indicating that the average expression of each gene within brown4 is increased in MSI-H versus MSS/MSI-L tumors (Figure 7). Brown4 was of particular interest given that this large module contained 81 of the 556 novel DEGs identified in our single-gene approach. We used pathway analysis to determine the molecular functionality of brown4. Here, we found that many of the Gene Ontology [24, 25] biological processes enriched in this module corresponded to apoptosis, such as positive regulation of apoptotic signaling pathways (*q* = 2.00E^-03^) and intrinsic apoptotic response to DNA damage (*q* = 0.013). Of the top 20 genes with the greatest module membership to this module, 10 were deemed to be novel in our single-gene analysis (*ZNF628, DAPK3, TMEM259, INAFM1, RPUSD1, CAPN15, UBALD1, MAP1S, ZBTB45* and *ADAT3*). We uploaded genes within the brown4 module to CHEA3 in an attempt to identify transcription factors that may be driving this module [32]. Here, we identified *ZBTB45* as the transcription factor most likely to regulate brown4, a novel gene with high module membership (*r* = 0.78, *P* = 1.48E^-60^). Taken together, WGCNA reveals novel insight into aberrant pathway activation between MSI-H and MSS/MSI-L tumors, which may lead to better understanding of tumor subtypes.

## DISCUSSION

We demonstrate the utility of single-cell deconvolution of bulk RNA-seq to aid in the interrogation of cellular heterogeneity of tumors using single-cell RNA-seq data from normal tissue. We first aimed to determine cell type composition differences between MSI-H and MSS/MSI-L CRC tumors. We replicated a number of known findings, including increased CD8^+^T cells and macrophages in MSI-H tumors, which are consistent with results of a meta-analysis of MSI-H tumors across several microarray datasets [33], as well as in MMR deficient CRC tumors [34]. CD8^+^T tumor infiltrating lymphocytes (TIL)s were also seen in greater number in MSI-H tumors [35]. Further, we were able to identify novel changes in cell composition including an increase in EEC and a reduction in stem and colonocyte cell populations in MSI-H versus MSS/MSI-L tumors. It is unclear whether these differences contribute to the etiology of MSI-H tumors. Importantly, we were able to replicate differences in cell content for three of the 13 cell types identified in TCGA-COAD (stem cell, cycling TA cells and macrophages) in independent datasets and provide some evidence for replication for an additional two cell types (CD8^+^T cell and colonocyte) that trended in the same direction. While this provides some evidence of replication, additional validation in a larger, independent cohort should still be considered an important step to improve the generalizability of our findings.

To the best of our knowledge, changes in EEC content have not yet been described as a distinguishing feature between MSI-H and MSS/MSI-L tumors. EECs are sensory cells that play a fundamental role in the orchestration of mucosal immunity by modulating activity of several immune cell types [36]. Despite comprising only approximately 1% of the gut, these cells form the largest endocrine system in humans, while also aiding in the maintenance of the stem cell niche [36]. Previous studies have identified a subpopulation of EECs that can either migrate to the small intestinal crypt base, or remain localized there [37]. Additional research in the small intestine has shown that pre-terminal EECs are able to reconstitute LGR5^+^ stem cells upon stem cell loss [38]. It is therefore possible that the increase in EECs reflects a population aiming to reconstitute a diminished stem cell pool, but additional work will be required to confirm this.

A reduction in colonocyte cell content was observed in MSI-H versus MSS/MSI-L tumors in both TCGA-COAD and GSE146889. Colonocytes (enterocytes of the colon) are the most abundant epithelial cell type of the colon, primarily functioning to facilitate the absorption of nutrients and water [39]. Both *CDX2* and *HNF1A* have previously been shown to play a role in enterocyte differentiation [40, 41]. Our initial analysis found that both of these genes, as well as a number of other genes required for enterocyte differentiation such as *GADD45GIP1* and *ELF3* [42], were significantly downregulated in MSI-H versus MSS/MSI-L tumors prior to adjustment for cell composition. Unsurprisingly however, these cell-specific genes did not remain significant following adjustment for cell composition. It remains unclear whether the reduction in colonocyte cell population is a function of reduced expression of absorptive transcriptional activators or due to other physiological constraints such as colon location, despite efforts to correct for this in our regression models. Two intriguing additional possibilities should be considered. The first, as hypothesized with enteroendocrine cells, colonocyte precursors may undergo dedifferentiation to replenish the stem cell pool, as has been shown to occur in enterocytes of the small intestine [43]. Over time, this may significantly reduce the availability of colonocyte populations. The second is that the reduced content observed here may also contribute to, not replenish the observed stem cell reduction. Under physiological conditions, differentiated colonocytes act to metabolize butyrate, leading to the establishment of an oxygen-butryate gradient along the crypt axis. Adequately maintaining a stable oxygen-butyrate gradient is vital for the protection of stem cells, as an increase in butryare has been shown to reduce their poliferative ability [39]. Butyrate is frequently associated with reduced tumor growth and is generated through the gut microbiota. Distinct patterns of gut microbiota have been associated with MSI-H status [44]. However, it remains unknown if differences in the butyrate concentration gradient occur and if so, how they may be able to better define MSI-H tumors.

Previously, we have shown that correction for cell composition can reduce the impact of cell variation in DEG reporting of a colon organoid model exposed to ethanol [7]. Here, we use a similar approach to identify 3,193 DEGs in our regression of MSI-H versus MSS/MSI-L tumors, of which 556 were not significant prior to adjustment, and as such were deemed to be novel. Pathway analysis of DEGs displaying reduced expression in MSI-H tumors revealed an enrichment for the DNA repair pathway. Importantly, pathway analysis of genes no longer considered to be significanct following adjustment for cell compostion did not identify enrichments for repair pathways. Instead these pathways were enriched for cell-specific processes such as T-cell activation. Together, these findings indicate that adjusting for cell composition enriches for biological signals that affect the system as a whole. Hypermethylation of *MLH1* is primarily considered to be the hallmark of non-familial MSI-H tumors, while mutations in *MLH1,* *MSH2*, *MSH6* and to a lesser extent, *PMS1*, are known to drive Lynch syndrome, an inherited condition that increases the risk primarily for MSI-H-related CRC [23]. However, two recent studies have shown that reduced expression of *MSH2* and *MSH6* protein have also been identified in sporadic CRC [45, 46]. While we were unable to replicate differences in these genes in colon cancer cell lines, their identification in TCGA-COAD following the adjustment for cell composition does reflect the importance of performing deconvolution methods.

Beyond differential expression of DNA repair genes, highly significant reductions in the expression of *AGMO*, *LINC02577* and *KIF1A* in MSI-H tumors may be worth further consideration. These genes represent the three most significant novel genes that were found to be replicated in our analysis of colon cancer cell lines. Differential expression of *LINC02577* has recently been associated with CRC [47, 48], though to the best of our knowledge this gene has not been found to be differentially expressed in MSI-H tumors. Long non-coding RNAs have a variety of molecular functions, but are frequently regarded as a “sponge” for microRNAs, thus reducing their bioavailabilty to regulate the expression of downstream mRNA targets [49]. To further understand the role that this gene may play in MSI-H tumor biology, further studies should look to incorporate additional omic layers. Little is also known about the role *AGMO* may play in cancer, which primarily functions to aid in the synthesis of membrane lipids. However, recent studies have indicated a potential role for this gene in the regulation of Wnt secretion [50, 51]. Correct regulation of Wnt signaling is vital to the maintenance of healthy rates of stem cell proliferation and differentiation. Aberrant activation of the Wnt signaling pathway has been shown to be a major driver of colon cancer [52]. Further, MSS tumors are more likely to be driven through aberrant activation of Wnt signaling genes [53]. Reduced expression of *AGMO* may therefore have an important role in distinguishing MSI-H from MSS/MSI-L tumors and may contribute to the reduction in stem cell content observed by reducing Wnt activation. *KIF1A* has been associated with head and neck squamous cell carcinoma [54] and has an important role in cell division. Pathway analysis of the novel downregulated DEGs identified in our analysis revealed a number of enrichments for cell division processes, indicating that variation in this pathway may be somewhat important in distinguishing MSI-H and MSS/MSI-L tumors. Indeed, a failure of the mismatch repair system to verify microsatellite repeat counts during cell division leads to variation in the length of their sequences. However, it remains unclear as to how reduced expression in *KIF1A* may affect MSI-H tumors.

To further interrogate the transcriptomic variation between MSI-H and MSS/MSI-L tumors, we performed WGCNA [26]. This method employes a level of guilt by association. For example, *MLH1* was central to the structure of blueviolet, the most significantly different module identified between MSI-H and MSS/MSI-L tumors. Thus, genes within this module may be critical to distinguishing MSI-H from MSS/MSI-L tumor biology. *PTPRD* was also found within this module. This gene plays an important role in the regulation of cell growth and differentiation [30], which make it an interesting target for further consideration in poorly differentiated tumors. Further, mutations in *PTPRD* have recently been shown to be frequent in T-cell rich B-cell lymphomas that display *MLH1* haploinsufficiency [55], adding weight to the validity of our co-expression analysis. Together, these data highlight the potential importance of this gene and its interplay with *MLH1* in MSI-H tumors.

The identification of the brown4 module was of particular interest given that it contained 15.64% of the novel DEGs identified within our single-gene analysis, including *ZBTB45*. *ZBTB45* was identified as one of the top 20 most significant hub genes for brown4, indicating an important role for this gene in a module enriched for apoptosis-related pathways. This result was confirmed using CHEA3 [32], an online tool that aims to identify, rank and prioritize regulatory transcription factors that may be affecting a given set of genes. Increased rates of apoptosis have previously been reported in MSI-H versus MSS or MSI-L tumors [56], where the authors were unable to fully attribute the increased apoptotic index to an increase in TILs. Improving our understanding of transcriptional networks that drive changes in apoptosis between tumor subtypes may help to explain the survival advantage generally observed in MSI-H tumors [56].

There are a number of limitations to this study. In our analysis, we used a consensus measure of tumor purity to control for tumor heterogeneity across samples. However, while we are able to infer the cell types present within TCGA-COAD and simulatenously account for tumor heterogeneity, we were not able to specify the origins of these cell types, i.e., intra-tumoral versus intra-epithelial. In many instances, our definition of cell type populations is also limited to either the resolution defined by the scRNA-seq study used for deconvolution [9], or the resolution of cell populations that could be delineated accurately through deconvolution. Subpopulations of EECs could not be defined using our approach. EECs consists of multiple sub-lineages, often classified by their principal hormone product. These secretory cell populations vary in density across the gastrointestinal tract [57]. We also do not consider the potentially confounding effects of other cancer related molecular pathways in our analysis, such as CpG Island Methylator Phenotype (CIMP). There is considerable overlap between MSI-H status and high levels of CIMP (CIMP-H). However, CIMP-H has also been observed in a subset of MSS tumors [58]. Finally, we do not consider the role of somatic mutations in driving expression. However, adopting such approaches in future, larger studies may provide additional insight into MSI-H tumors.

In summary, we employ a machine learning approach to deconvolute MSI-H and MSS/MSI-L tumor gene expression across TCGA-COAD and two additional cohorts. We identify novel changes in cell composition for EECs and colonocytes that suggests previously uncharacterized roles for these cell populations in contributing to MSI-H tumor development. Finally we use both single-gene and network analysis to identify several novel genes that may play an important role in MSI-H tumor biology.

## MATERIALS AND METHODS

### RNA-seq data pre-processing

HT-Seq count and phenotype data were downloaded from the R package TCGAbiolinks [59]. For data collection, pre-processing and alignment details, please refer to the original publications [8, 59, 60]. Single-cell deconvolution of bulk RNA-seq has been shown to perform best on larger datasets [6]. Thus, we first used a total 409 samples to estimate cell populations in CRC tumors. For the analysis of gene expression differences in MSI-H versus MSS/MSI-L tumors, a total of 294 samples were considered (MSI-H = 63, MSS = 178, MSI-L = 53). Samples were removed if they had missing phenotype information for MSI status, consensus purity estimates, tumor stage, or lacked specific colon location information, i.e., colon location data was labelled either “NA” or “colon, NOS”. Cancer stages were broadly categorized into main hierarchical groupings (stage 1–4). Samples were also broadly categorized into one of three location groupings: left (descending, sigmoid, splenic flexure), right (ascending, cecum, hepatic flexure) and transverse, which were considered based upon the developmental origins of colon tissue. Given that the transverse colon is derived from either midgut or hindgut (depending on which region of the transverse colon), we considered this a distinct colon segment. Consensus purity estimates were downloaded from a previously published analysis of TCGA-COAD [61].

We identified a second, smaller CRC cohort with available MSI data on June 1st, 2020, by searching gene expression omnibus (GEO) [62] using keywords “MSI” and “colorectal cancer” and only considered data generated using RNA-seq. This dataset also contained RNA-seq count data for endometrial cancer, which was not considered here. Of note, the majority of MSI-H individuals considered in this dataset were putative Lynch syndrome (32/36), while the majority of TCGA-COAD is considered to be sporadic MSI-H tumors. Raw counts were downloaded from GEO, accession: GSE146889 for downstream analysis.

For further validation, we also downloaded RNA-seq count and TPMs from the Broad CCLE website (https://portals.broadinstitute.org/ccle). Details for RNA-seq library generation and pre-processing can be found in the original article [11].

### Single-cell deconvolution of bulk RNA-seq data

We downloaded publicly available scRNA-seq data derived from normal colon biopsies [9]. To reduce heterogeneity in single-cell expression, only cells derived from healthy colon were considered for this analysis. Transcripts per million (TPM)s were generated using scater [63]. Given the size of the dataset, cells were randomly downsized to permit upload to CIBERSORTx [6]. For model evaluation, cell composition scores were correlated to known gene expression markers in an attempt to determine relative performance.

The final dataset consisted of 19,567 genes across 5,412 cells. Multiple similar cell types were merged to aid in this analysis, for example: B cells (plasma, germinal center, follicular). M cells were removed due to low abundance in the original analysis (*n* = 10). Tuft cells were removed after multiple attempts to define population led to inadequate identification of cell population markers. Secretory TA cells were removed given their similar transcriptional profile to mature secretory cell populations and cycling TA cells. TA1 and TA2 cell populations were also not considered, given their similarity to cycling TA cells. Epithelial progenitor cells of goblets and colonocytes were also removed in favor of their mature cell populations to aid in their distinction from cycling TA cells. A total of 19 distinct cell types were considered in the final analysis. We note that this represents a reduction in granularity from the 51 unique cell types identified in scRNA-seq analysis of normal colon [9].

Following upload to CIBERSORTx [6], single cells were clustered based upon overall similarity of expression using default parameters, with the following notable exceptions: minimum expression = 0; number of significant genes to define cell type = 150; sampling = 1, *q* = 0.001. Following this, TPMs from TCGA-COAD samples were imported and cell composition scores were estimated. For deconvolution the following parameters were set: 500 permutations; quantile normalization was disabled; S-mode was set for batch correction; scores were generated in absolute mode. Cell scores were then centered and scaled about the mean prior to incorporation as covariates in a regression model. The same parameters were also used for deconvolution of GSE146889 [10].

For colon cancer cell lines [11], a total of 3,988 cells across four epithelial cell types were considered for deconvolution (cycling TA, stem cell, colonocyte progenitors and immature goblets). Deconvolution was performed as above, with one exception: the number of genes used to define cell types was set to a range of 100–600.

### Regression analysis

Differentially expressed genes DEGs were identified through regression analysis performed in DESeq2 [64]. Several regression models were used in this study.

#### Differential expression

For the analysis of cell type agnostic differential expression we used the following model:

*Expression* ~ *Stage* + *Sex* + Consensus Purity Estimate + *Colon* *Location* + *Scores* + *MSI*

Where: expression = gene expression of each gene for each individual; score = cell score (continuous variable); stage = cancer stage (factored 1–4); sex = biological sex; consensus purity estimate = tumor purity (continuous variable); colon location = location of biopsy taken (factor); MSI = microsatellite instability status.

#### Cell composition

To analyze differences in absolute cell scores between MSI-H and MSS/MSI-L a linear regression model was used.

*Score* ~ *Stage* + *Sex* + *Consensus Purity Estimate* + *Colon* *Location* + *MSI*

Where: score = cell score for each cell type and individual (continuous); stage = cancer stage (factored 1-4); sex = biological sex; consensus purity estimate = tumor purity (continuous variable); colon location = location of biopsy taken (factor); MSI = microsatellite instability status.

### WGCNA

Genes with a count of less than 10 in 150 samples were filtered, leaving a total of 17,361 genes for downstream network analysis. Raw counts were converted into counts per million and the effects of tumor stage (factor), sex, colon location (factor), tumor purity and cell compositon were regressed out prior to WGCNA [26] using limma [65]. Heirarchical clustering analysis was used to determine outliers based on their average dissimilarity, which led to the removal of four samples. A total of 289 samples and 17,361 genes were therefore used to construct the network. WGCNA was performed under default settings with the exception of the following parameters: a soft power of four was chosen, where the degree of independence was determined to be 0.876; blocksize was set to the number of genes used; signed hybrid and pearson correlation were preferred; minimum module size was set to 10; deep split was set to 3 and strongly correlated modules (*r* = 0.7) were merged prior to association testing.

## SUPPLEMENTARY MATERIALS















## Abbreviations

5-FU: 5-fluorouracil
CCLE: Cancer Cell Line Encyclopedia
CIMP: CpG Island Methylator Phenotype
CIMP-H: (CpG Island Methylator Phenotype high)
CIN: chromosomal instability
CRC: colorectal cancer
DEG: differentially expressed gene
EEC: enteroendocrine cell
MMR: mismatch repair
MSI: microsatellite instability
MSI-H: microsatellite instability high
MSI-L: microsatellite instability low
MSS: microsatellite stable
RNA-seq: RNA-sequencing
scRNA-seq: single-cell RNA-sequencing
TA: transit amplifying
TCGA-COAD: The Cancer Genome Atlas Colon Adenocarcinoma
TIL: tumor infiltrating lymphocyte
TPM: transcript per million
