# Supplementary material for: Controlling for cellular heterogeneity using single-cell deconvolution of gene expression reveals novel markers of colorectal tumors exhibiting microsatellite instability
Source: Oncotarget. 2021 Apr 13;12(8):767–82. doi: 10.18632/oncotarget.27935 (PMC8057268; doi:10.18632/oncotarget.27935)
Supplement: Supplementary file 1 [file oncotarget-12-767-s001.pdf]

# Controlling for cellular heterogeneity using single-cell deconvolution of gene expression reveals novel markers of colorectal tumors exhibiting microsatellite instability

## SUPPLEMENTARY MATERIALS

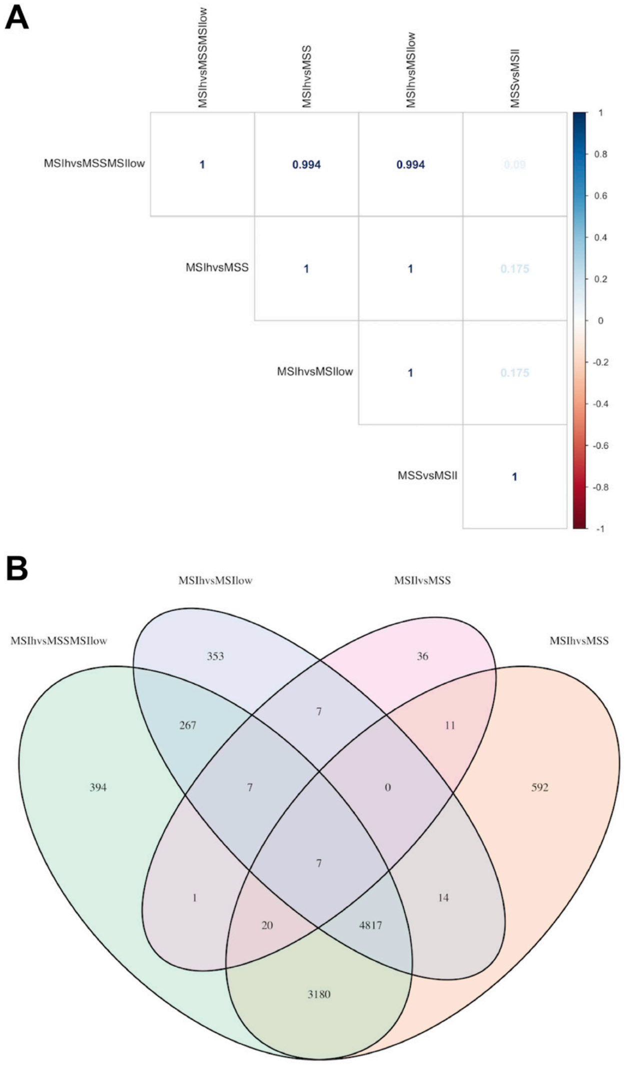


**Supplementary Figure 1: Determination of TCGA-COAD comparisons.** (**A**) Test-statistics for each regression were correlated to determine the similarity of global transcriptomic response between analyses. (**B**) Significant DEGs ($q = 0.05$) identified in each regression were overlaid using a Venn diagram to determine the extent of overlap.

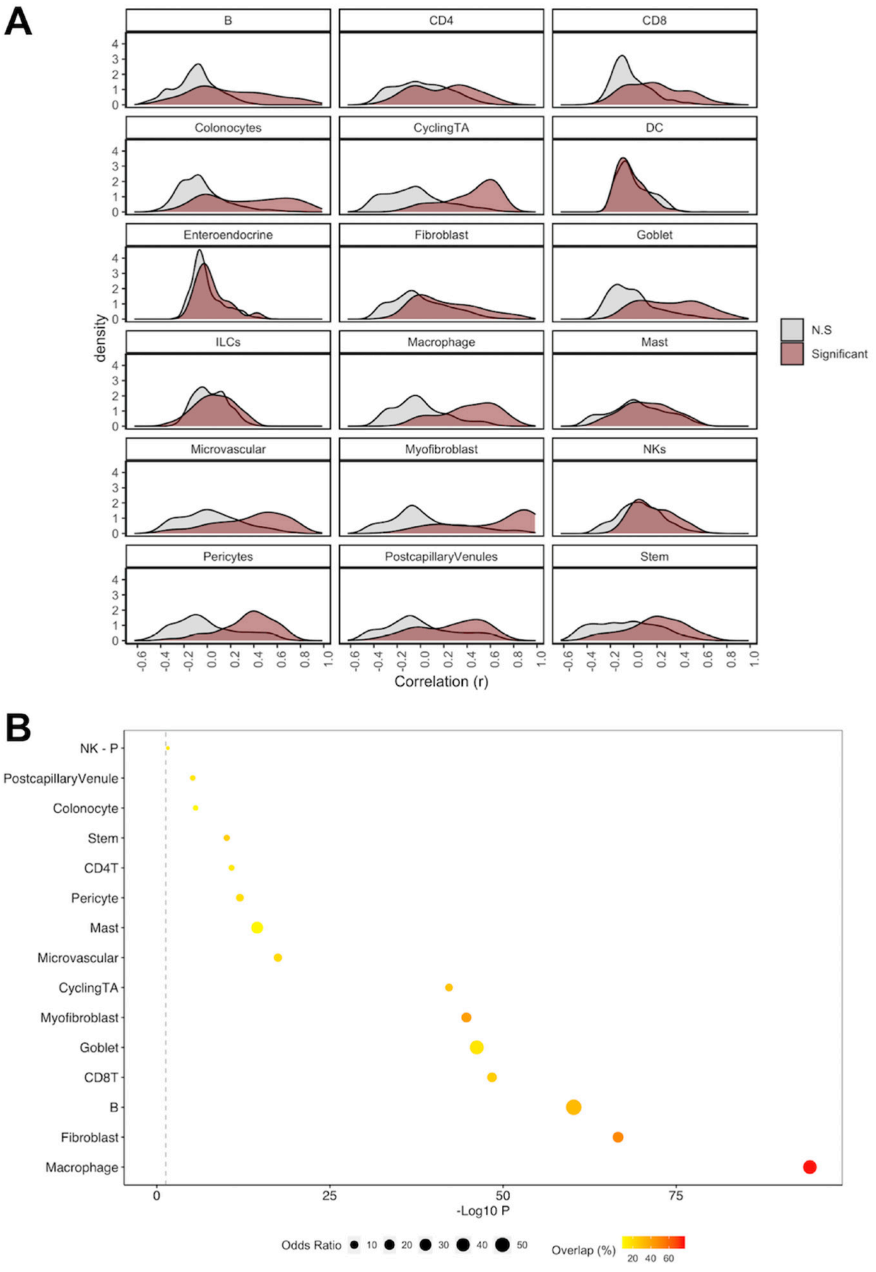


**Supplementary Figure 2: Single-cell deconvolution of GSE146889 CRC tumors.** (**A**) Cell scores were correlated to markers of cell types. Correlations between cell scores and significant markers of that cell type are shown in red. For background, correlations between a cell score and markers of other cell types were displayed in grey. (**B**) Summary of enrichment analysis (one-way Fisher's exact test) for cell type markers in differential expression analysis of cell scores. Grey line represents $\log_{10}(0.05)$. Percentage overlap reflects percentage of cell type markers for a given cell type that were significant within regression of cell score. For NK cells, enrichment was only identified using nominally significant DEGs ($P = 0.05$).

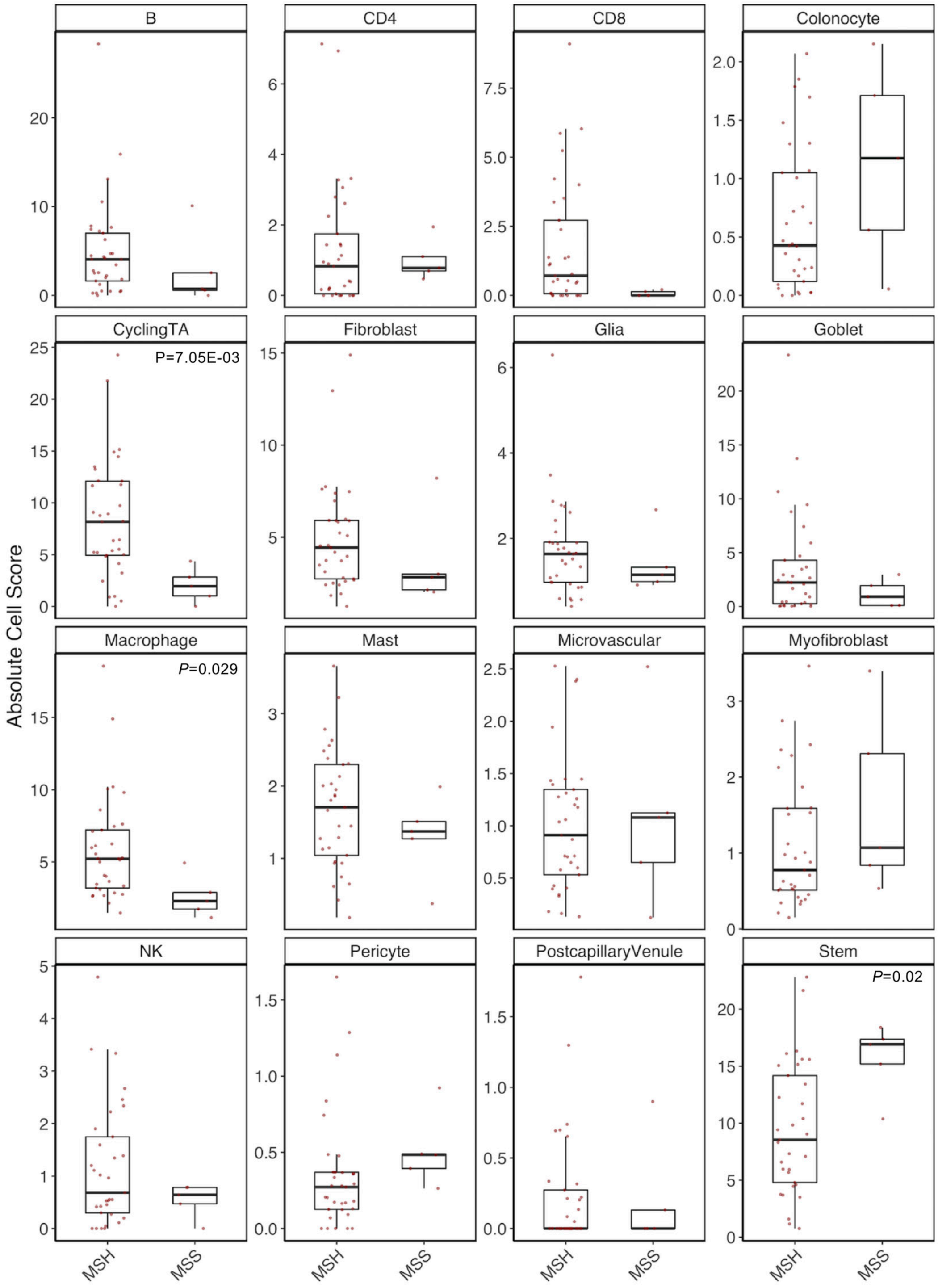


**Supplementary Figure 3: Summary of cell score regressions on MSI status in GSE146889 CRC tumor dataset.**

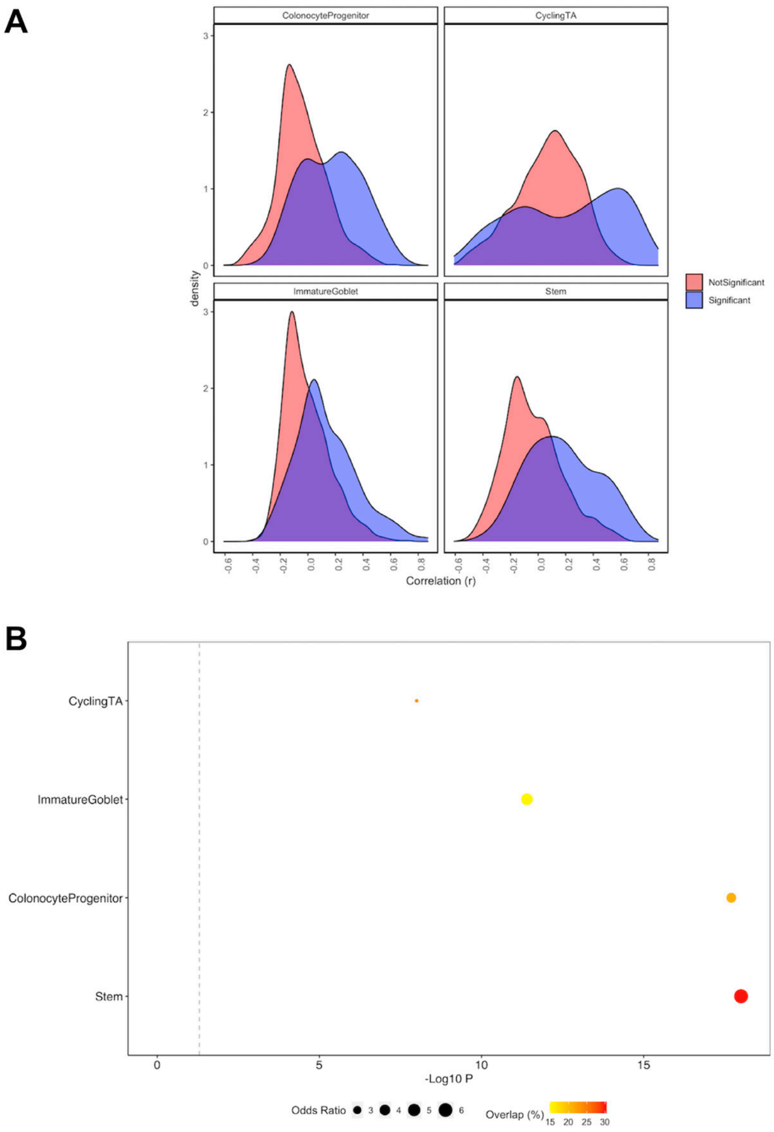


**Supplementary Figure 4: Single-cell deconvolution of CCLE colon cancer cell lines.** (**A**) Cell scores were correlated to markers of cell types. Correlations between cell scores and significant markers of that cell type are shown in blue. For background, correlations between a cell score and markers of other cell types were displayed in red. (**B**) Summary of enrichment analysis (one-way Fisher's exact test) for cell type markers in differential expression analysis of cell scores. Grey line represents $\log_{10}(0.05)$. Percentage overlap reflects percentage of cell type markers for a given cell type that were significant within regression of cell score.

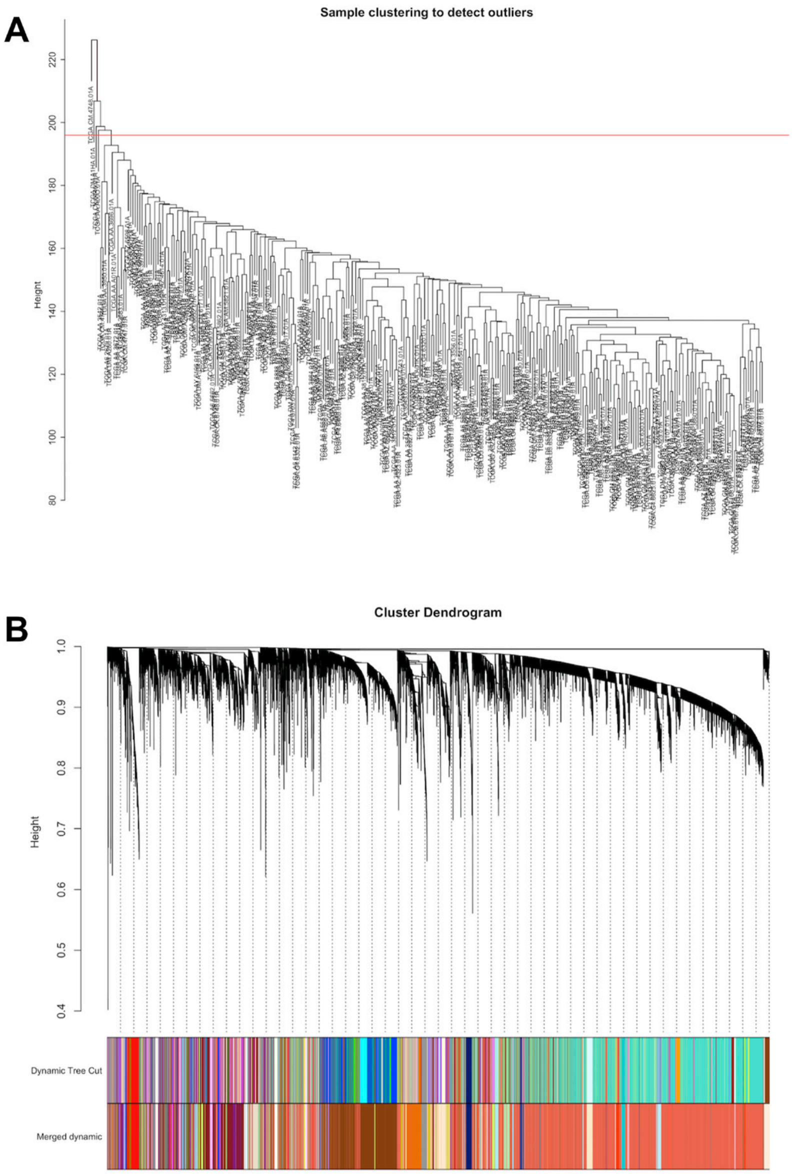


**Supplementary Figure 5: Overview of WGCNA performed in TCGA-COAD dataset.** (**A**) Hierarchical clustering identified four samples as potential outliers based on their dissimilarity to other samples. These four samples were removed. (**B**) Adjacency matrix was raised to the power of 4 and transformed into a topological overlap matrix. Hierarchical clustering was performed on this matrix. Genes with high levels of co-expression are grouped. Module colors were assigned (dynamic tree cut) and modules that are highly co-expressed ($r = 0.7$) were merged (merged dynamic). These merged modules were used for downstream association testing.

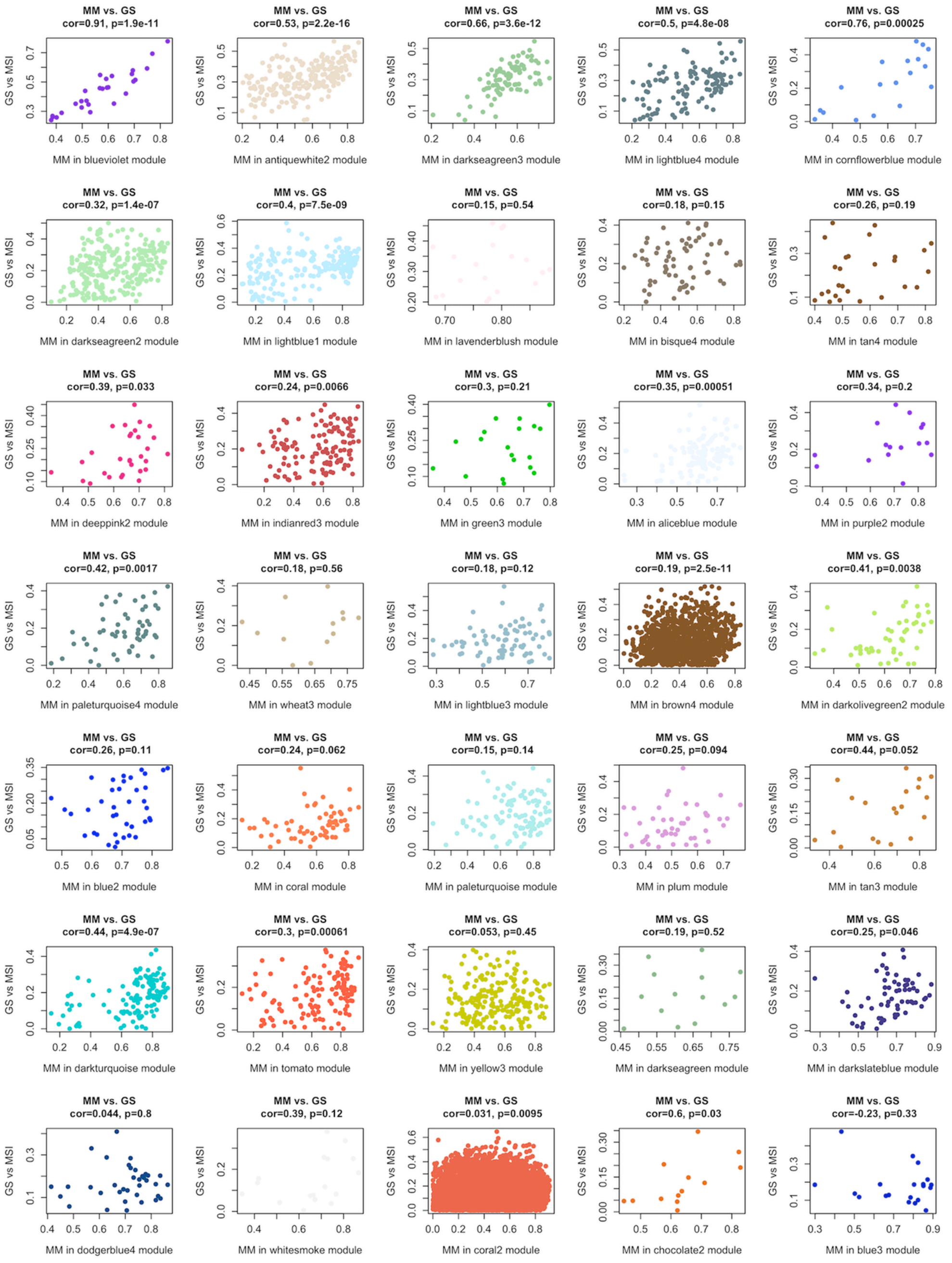


**Supplementary Figure 6: Summary of correlation results between gene significance and module membership for each of the 35 modules significantly associated with MSI status ($q$ = 0.05).**

**Supplementary Table 1: Overlap of cell type markers with DEGs identified in MSI-H vs MSS/MSI-L analysis of TCGA-COAD cohort**

| Cell Type | No. DEGs overexpressed in MSI-H | No. DEGs underexpressed in MSI-H |
|---|---|---|
| B cell | 37 [3] | 16 [4] |
| CD4T | 42 [4] | 7 [0] |
| CD8T | 60 [1] | 3 [1] |
| Colonocyte | 45 [2] | 101 [6] |
| CyclingTA | 91 [9] | 28 [4] |
| DC | 59 [4] | 13 [1] |
| Enteroendocrine | 16 [1] | 17 [2] |
| Fibroblast | 49 [3] | 107 [10] |
| Glia | NA$^+$ | NA |
| Goblet | 52 [6] | 29 [2] |
| ILCs | 18 [1] | 13 [2] |
| Macrophages | 80 [2] | 9 [1] |
| Mast | 29 [6] | 17 [1] |
| Microvascular | 22 [3] | 13 [7] |
| Myofibroblasts | 10 [2] | 20 [1] |
| NKs | 16 [0] | 1 [0] |
| Pericytes | 8 [0] | 26 [1] |
| Postcapillary Venules | 18 [3] | 15 [1] |
| Stem | 9 [7] | 41 [2] |

Values without brackets represent FDR corrected DEGs ($q = 0.05$). Values in brackets represent the number of additional DEGs that were nominally significant ($P = 0.05$) that did not reach FDR correction. $^+$Cell markers not available.

**Supplementary Table 2: Correlation of cell-type expression markers to cell scores generated in each approach.** See Supplementary Table 2

**Supplementary Table 3: Cell type agnostic DEGs found to be significant ($q = 0.05$) in regression analysis of MSI status in TCGA-COAD data that were replicated in similar analysis of CCLE ($P = 0.05$).** See Supplementary Table 3

**Supplementary Table 4: Overview of modules identified in WGCNA.** See Supplementay Table 4

**Supplementary Table 5: Novel significant DEGs ($q = 0.05$) identified in TCGA-COAD regression of MSI-H vs MSS/MSI-L tumors following adjustment for cell composition that were replicated in a similar analysis of colon cancer cell lines ($P = 0.05$).** See Supplementary Table 5
