# Supplementary material for: Controlling for cellular heterogeneity using single-cell deconvolution of gene expression reveals novel markers of colorectal tumors exhibiting microsatellite instability
Source: Oncotarget. 2021 Apr 13;12(8):767–82. doi: 10.18632/oncotarget.27935 (PMC8057268; doi:10.18632/oncotarget.27935)
Supplement: Supplementary file 2 [file oncotarget-12-767-s002.docx]

**Supplementary Table 2: Correlation of cell-type expression markers to cell scores generated in each approach.** Median and interquartile range for all correlations was calculated for genes found to be most significantly expressed in that cell-type and those most significantly expressed in other cell-types, as defined through scRNA-seq. Methods ranked in order of largest difference between median correlations. **A)** B Cell **B)** CD4+T Cell **C)** CD8+T cell **D)** NK cell **E)** Macrophage **F)** Dendritic Cell **G)** Fibroblast. *P*-values recorded at 0 were less that of P=2.2E^-16^, the smallest floating-point number recorded in R.

**(A)**

|  | Significant | | Non-Significant | |  |
| --- | --- | --- | --- | --- | --- |
| Method | **R** | **P** | **R** | **P** | **Shift** |
| mcpCounter | 0.35  [0.05,0.58] | 5.78E^-13^  [0, 0.04] | 0.06  [-0.06, 0.23] | 5.28E^-03^  [1.82E-06, 0.22] | 0.29 |
| EPIC | 0.27  [0.05,0.58] | 3.45E^-08^  [0, 0.11] | 0.05  [-0.04, 0.17] | 0.046  [3.01E^-04^, 0.37] | 0.22 |
| xCell | 0.20  [0.04,0.49] | 5.07E^-05^  [0, 0.13] | 0.03  [-0.04, 1.13] | 0.12  [4.93E^-03^, 0.49] | 0.17 |
| Present | 0.16  [0.02,0.32] | 4.24E^-04^  [1.90E^-10^, 0.13] | 0.02  [-0.05, 0.09] | 0.12  [2.78E-03, 0.51] | 0.14 |
| Quantiseq | 0.21  [0.07,0.47] | 1.06E^-05^  [0, 0.11] | 0.07  [-0.03, 0.18] | 0.03  [7.21E^-05^, 0.31] | 0.14 |

**(B)**

|  | Significant | | Non-Significant | |  |
| --- | --- | --- | --- | --- | --- |
| Method | **R** | **P** | **R** | **P** | **Shift** |
| Present | 0.31  [0.08, 0.45] | 1.78E-10  [0,0] | 0.06  [-0.05, 0.29] | 6.00E^-03^  [3.53E^-09^, 0.26] | 0.25 |
| Quantiseq | 0.33  [0.12, 0.50] | 2.10E-11  [0,0] | 0.10  [-0.03, 0.27] | 2.78E-03  [6.99E^-08^, 0.22] | 0.23 |
| EPIC | 0.28  [0.12, 0.39] | 1.42E^-08^  [0,0] | 0.06  [-0.05, 0.18] | 0.02  [3.21E^-05^, 0.27] | 0.22 |
| xCell | 0.01  [-4.54E^-03^, 0.05] | 0.69  [7.23E^-04^, 0.79] | -4.58E^-03^  [-0.02, 0.02] | 0.68  [0.47, 0.84] | 0.015 |
| mcpCounter | NA^#^ | NA | NA | NA | NA |

# Not available

**(C)**

|  | Significant | | Non-Significant | |  |
| --- | --- | --- | --- | --- | --- |
| Method | **R** | **P** | **R** | **P** | **Shift** |
| Present | 0.47  [0.15, 0.67] | 0  [0, 8.31E^-04^] | 0.03  [-0.06, 0.14] | 0.048  [1.74E^-04^, 0.37] | 0.44 |
| Quantiseq | 0.45  [0.16, 0.69] | 0  [0, 5.74E^-04^] | 0.05,  [-0.06, 0.16] | 0.04,  [9.54E^-05^, 0.31] | 0.40 |
| xCell | 0.37  [0.13, 0.63] | 2.40E-14  [0, 1.83E^-03^] | 0.02  [-0.05, 0.13] | 0.10  [1.20E^-03^, 0.46] | 0.27 |
| mcpCounter | 0.28  [0.09, 0.39] | 1.17E^-08^  [4.44E^-16^, 0.02] | 0.04  [-0.03, 0.13] | 0.11  4.13E^-03^, 0.46] | 0.17 |
| EPIC | -6.88E^-03^  [-0.07, -0.04] | 0.30 [  0.03, 0.64) | 0.03  [-0.14, 0.06] | 0.07  [6.64E^-04^, 0.41] | -0.04 |
| Present | 0.47  [0.15, 0.67] | 0  [0, 8.31E-04] | 0.03  [-0.06, 0.14] | 0.048  [1.74E^-04^, 0.37] | 0.44 |

**(D)**

|  | Significant | | Non-Significant | |  |
| --- | --- | --- | --- | --- | --- |
| Method | **R** | **P** | **R** | **P** | **Shift** |
| EPIC | 0.13  [0.02,0.26] | 4.01E-03  [1.48E^-07^, 0.22] | 5.16E^-03^  [-0.05, 0.09] | 0.18  [0.01, 0.55] | 0.12 |
| Present | 0.10  [0.04, 0.20] | 0.014  [1.71E^-05^, 0.16] | 0.03  [-0.05, 0.16] | 0.06  [2.92E^-04^, 0.39] | 0.04 |
| xCell | 0.02  [-1.98E^-03^, 0.08] | 0.43  [0.11, 0.86] | -3.06E^-03^  [-0.02, -0.04] | 0.60 [  0.33, 0.79] | 0.02 |
| mcpCounter | 0.04  [-0.01, 0.10] | 0.32  [0.06, 0.56] | 0.02  [-0.02, 0.06] | 0.37  [0.12, 0.69] | 0.02 |
| Quantiseq | -0.12  [-0.18, 3.68E^-03^] | 2.99E-03  [2.58E^-03^, 0.26] | -0.06  [-0.13, 0.04] | 0.048  [1.16E^-03^, 0.32] | -0.18 |

**(E)**

|  | Significant | | Non-Significant | |  |
| --- | --- | --- | --- | --- | --- |
| Method | **R** | **P** | **R** | **P** | **Shift** |
| xCell | 0.61  [0.34, 0.78] | 0  [0, 9.17E^-12^] | 0.03  [-0.08, 0.26] | 3.14E^-03^  [1.78E^-08^, 0.21] | 0.58 |
| Present | 0.63  [0.58, 0.69] | 0  [0,0] | 0.06  [-0.08, 0.29] | 1.78E^-03^  [5.05E^-10^, 0.15] | 0.57 |
| EPIC | 0.60  [0.36, 0.73] | 0  [0, 5.68E^-13^] | 0.05  [-0.08, 0.28] | 1.98E^-03^  [3.05E^-09^, 0.17] | 0.55 |
| Quantiseq | 0.42  [0.24, 0.51] | 0  [0, 1.28E^-06^] | 0.07  [-0.05, 0.22] | 7.71E^-03^  [1.74E^-06^, 0.24] | 0.35 |
| mcpCounter | NA^#^ | NA | NA | NA | NA |

# Not available

**(F)**

|  | Significant | | Non-Significant | |  |
| --- | --- | --- | --- | --- | --- |
| Method | **R** | **P** | **R** | **P** | **Shift** |
| xCell | 0.38  [0.18, 0.58] | 3.00E^-15^  [0, 2.61E^-04^] | 0.06  [-0.08, 0.28] | 1.97E^-03^  [2.75E^-09^, 0.16] | 0.32 |
| mcpCounter^#^ | 0.24  [0.10, 0.31] | 1.32E^-06^  [1.83E^-10^, 7.00E^-03]^ | 0.08  [-0.04, 0.19] | 9.55E^-03^  [2.36E^-05^, 0.25] | 0.16 |
| Present | 0.17  [0.05, 0.29] | 4.94E^-04^  [3.17E-09, 0.13] | 0.03  [ -0.07, 0.14] | 0.04  [4.87E^-04^, 0.32] | 0.14 |
| Quantiseq^#^ | 5.20E^-04^  [-0.04, 0.05] | 0.43  [0.17, 0.78] | 0.01  [-0.03, 0.06] | 0.34  [0.10, 0.65] | -0.01 |
| EPIC | NA^+^ | NA | NA | NA | NA |

# Myeloid dendritic cell

+ Not available

**(G)**

|  | Significant | | Non-Significant | |  |
| --- | --- | --- | --- | --- | --- |
| Method | **R** | **P** | **R** | **P** | **Shift** |
| EPIC^#^ | 0.39  [0.11, 0.62] | 1.44E^-15^  [0, 0.01] | 0.03  [-0.09, 0.28] | 1.47E^-03^  [6.52E^-10^, 0.19] | 0.36 |
| mcpCounter | 0.39  [0.12, 0.64] | 6.66E^-16^  [0, 7.73E-03] | 0.04  [-0.09, 0.29] | 3.39E-03  [2.73E^-09^, 0.21] | 0.35 |
| xCell | 0.34  [0.09, 0.51] | 6.61E^-12^  [0, 0.047] | 0.02  [-0.07, 0.25] | 0.02  [7.99E^-07^, 0.30] | 0.32 |
| Present | 0.31  [0.11, 0.27] | 9.09E^-11^  [0, 0.02] | 0.05  [-0.07, 0.27] | 2.28E^-03^  [7.39E^-05^, 0.21] | 0.26 |
| Quantiseq | NA^+^ | NA | NA | NA | NA |

# Cancer-associated fibroblasts

+ Not available
