# Supplementary material for: Controlling for cellular heterogeneity using single-cell deconvolution of gene expression reveals novel markers of colorectal tumors exhibiting microsatellite instability
Source: Oncotarget. 2021 Apr 13;12(8):767–82. doi: 10.18632/oncotarget.27935 (PMC8057268; doi:10.18632/oncotarget.27935)
Supplement: Supplementary file 3 [file oncotarget-12-767-s003.docx]

**Supplementary Table 3: Cell type agnostic DEGs found to be significant (q=0.05) in regression analysis of MSI status in TCGA-COAD data that were replicated in similar analysis of CCLE (*P*=0.05).** Negative log_2_Fold change corresponds to DEGs displaying significantly reduced expression in MSI-H tumors and cell lines.

| Ensembl ID | HGNC Symbol | TCGA-COAD | | | | | CCLE | | | | |
| --- | --- | --- | --- | --- | --- | --- | --- | --- | --- | --- | --- |
|  |  | log2Fold Change | lfcSE | Stat | *P* | FDR | log2Fold Change | lfcSE | Stat | *P* | FDR |
| ENSG00000184719 | RNLS | -1.452 | 0.258 | -5.619 | 1.92E-08 | 3.77E-06 | -4.850 | 0.692 | -7.007 | 2.43E-12 | 4.58E-09 |
| ENSG00000234284 | ZNF879 | -0.754 | 0.260 | -2.897 | 3.77E-03 | 0.028 | -5.799 | 0.865 | -6.707 | 1.99E-11 | 2.77E-08 |
| ENSG00000165443 | PHYHIPL | -1.761 | 0.583 | -3.022 | 2.51E-03 | 0.021 | -7.296 | 1.088 | -6.707 | 1.99E-11 | 2.77E-08 |
| ENSG00000129682 | FGF13 | -1.789 | 0.303 | -5.898 | 3.67E-09 | 1.04E-06 | -5.427 | 0.843 | -6.435 | 1.24E-10 | 1.47E-07 |
| ENSG00000198382 | UVRAG | -0.273 | 0.077 | -3.539 | 4.02E-04 | 5.83E-03 | -0.928 | 0.149 | -6.222 | 4.91E-10 | 5.43E-07 |
| ENSG00000198677 | TTC37 | -0.682 | 0.114 | -5.991 | 2.09E-09 | 7.04E-07 | -1.381 | 0.226 | -6.105 | 1.03E-09 | 9.99E-07 |
| ENSG00000171243 | SOSTDC1 | -2.713 | 0.404 | -6.713 | 1.91E-11 | 1.42E-08 | -5.656 | 0.967 | -5.848 | 4.97E-09 | 3.79E-06 |
| ENSG00000204889 | KRT40 | -1.757 | 0.610 | -2.879 | 3.99E-03 | 0.029 | -6.110 | 1.045 | -5.844 | 5.09E-09 | 3.79E-06 |
| ENSG00000117983 | MUC5B | 1.323 | 0.439 | 3.013 | 2.59E-03 | 0.022 | 3.607 | 0.647 | 5.576 | 2.46E-08 | 1.51E-05 |
| ENSG00000170271 | FAXDC2 | -0.835 | 0.211 | -3.953 | 7.73E-05 | 1.90E-03 | -3.707 | 0.669 | -5.540 | 3.03E-08 | 1.80E-05 |
| ENSG00000171827 | ZNF570 | -0.791 | 0.233 | -3.392 | 6.93E-04 | 8.61E-03 | -5.475 | 0.997 | -5.493 | 3.94E-08 | 2.21E-05 |
| ENSG00000203288 | TDRKH-AS1 | -1.286 | 0.197 | -6.534 | 6.38E-11 | 4.11E-08 | -1.839 | 0.335 | -5.483 | 4.17E-08 | 2.31E-05 |
| ENSG00000150471 | ADGRL3 | -1.218 | 0.301 | -4.045 | 5.24E-05 | 1.45E-03 | -4.569 | 0.844 | -5.414 | 6.17E-08 | 3.21E-05 |
| ENSG00000137673 | MMP7 | -2.327 | 0.411 | -5.658 | 1.53E-08 | 3.24E-06 | -5.836 | 1.078 | -5.413 | 6.20E-08 | 3.21E-05 |
| ENSG00000124228 | DDX27 | -0.549 | 0.112 | -4.882 | 1.05E-06 | 8.14E-05 | -1.056 | 0.198 | -5.336 | 9.48E-08 | 4.53E-05 |
| ENSG00000100354 | TNRC6B | 0.354 | 0.106 | 3.334 | 8.57E-04 | 0.010 | 0.991 | 0.189 | 5.244 | 1.57E-07 | 6.37E-05 |
| ENSG00000146021 | KLHL3 | -0.937 | 0.257 | -3.649 | 2.63E-04 | 4.36E-03 | -4.060 | 0.782 | -5.192 | 2.08E-07 | 7.93E-05 |
| ENSG00000153071 | DAB2 | -0.533 | 0.147 | -3.630 | 2.83E-04 | 4.60E-03 | -3.418 | 0.662 | -5.163 | 2.43E-07 | 9.04E-05 |
| ENSG00000181449 | SOX2 | -2.011 | 0.663 | -3.032 | 2.43E-03 | 0.021 | -4.701 | 0.911 | -5.159 | 2.49E-07 | 9.15E-05 |
| ENSG00000091129 | NRCAM | -1.206 | 0.384 | -3.138 | 1.70E-03 | 0.016 | -4.265 | 0.833 | -5.118 | 3.09E-07 | 1.09E-04 |
| ENSG00000198553 | KCNRG | 1.843 | 0.201 | 9.153 | 5.54E-20 | 5.17E-16 | 1.773 | 0.348 | 5.101 | 3.37E-07 | 1.16E-04 |
| ENSG00000100116 | GCAT | 0.713 | 0.165 | 4.323 | 1.54E-05 | 5.92E-04 | 1.855 | 0.365 | 5.078 | 3.81E-07 | 1.27E-04 |
| ENSG00000144214 | LYG1 | 1.621 | 0.221 | 7.347 | 2.03E-13 | 4.11E-10 | 2.213 | 0.444 | 4.985 | 6.18E-07 | 1.85E-04 |
| ENSG00000163531 | NFASC | -0.923 | 0.223 | -4.146 | 3.38E-05 | 1.05E-03 | -3.281 | 0.661 | -4.966 | 6.85E-07 | 1.98E-04 |
| ENSG00000163584 | RPL22L1 | 1.569 | 0.222 | 7.057 | 1.70E-12 | 2.11E-09 | 1.990 | 0.402 | 4.955 | 7.22E-07 | 2.06E-04 |
| ENSG00000197859 | ADAMTSL2 | -0.784 | 0.224 | -3.507 | 4.53E-04 | 6.37E-03 | -4.644 | 0.939 | -4.944 | 7.64E-07 | 2.17E-04 |
| ENSG00000169905 | TOR1AIP2 | 0.395 | 0.103 | 3.853 | 1.17E-04 | 2.54E-03 | 0.939 | 0.190 | 4.934 | 8.06E-07 | 2.24E-04 |
| ENSG00000168679 | SLC16A4 | -0.640 | 0.237 | -2.695 | 7.03E-03 | 0.044 | -2.460 | 0.502 | -4.902 | 9.50E-07 | 2.43E-04 |
| ENSG00000213062 |  | -1.419 | 0.217 | -6.536 | 6.33E-11 | 4.11E-08 | -1.916 | 0.391 | -4.896 | 9.77E-07 | 2.48E-04 |
| ENSG00000101115 | SALL4 | -1.037 | 0.263 | -3.946 | 7.95E-05 | 1.93E-03 | -3.869 | 0.791 | -4.894 | 9.90E-07 | 2.50E-04 |
| ENSG00000168243 | GNG4 | -2.955 | 0.403 | -7.336 | 2.20E-13 | 4.11E-10 | -4.275 | 0.876 | -4.880 | 1.06E-06 | 2.62E-04 |
| ENSG00000150551 | LYPD1 | -1.317 | 0.303 | -4.354 | 1.34E-05 | 5.33E-04 | -3.880 | 0.796 | -4.875 | 1.09E-06 | 2.65E-04 |
| ENSG00000146666 | LINC00525 | -1.473 | 0.301 | -4.896 | 9.79E-07 | 7.71E-05 | -2.881 | 0.599 | -4.811 | 1.51E-06 | 3.37E-04 |
| ENSG00000196437 | ZNF569 | -0.729 | 0.244 | -2.983 | 2.86E-03 | 0.023 | -3.605 | 0.754 | -4.780 | 1.75E-06 | 3.79E-04 |
| ENSG00000170099 | SERPINA6 | -2.368 | 0.520 | -4.551 | 5.33E-06 | 2.83E-04 | -4.300 | 0.906 | -4.748 | 2.06E-06 | 4.06E-04 |
| ENSG00000213246 | SUPT4H1 | 0.516 | 0.145 | 3.565 | 3.64E-04 | 5.42E-03 | 1.332 | 0.281 | 4.743 | 2.10E-06 | 4.13E-04 |
| ENSG00000161681 | SHANK1 | 0.716 | 0.269 | 2.658 | 7.87E-03 | 0.047 | 4.351 | 0.926 | 4.699 | 2.61E-06 | 4.76E-04 |
| ENSG00000174827 | PDZK1 | -1.545 | 0.324 | -4.770 | 1.84E-06 | 1.21E-04 | -3.178 | 0.677 | -4.693 | 2.69E-06 | 4.87E-04 |
| ENSG00000243955 | GSTA1 | -1.326 | 0.451 | -2.939 | 3.29E-03 | 0.026 | -4.596 | 0.980 | -4.691 | 2.72E-06 | 4.90E-04 |
| ENSG00000156076 | WIF1 | -2.807 | 0.703 | -3.994 | 6.49E-05 | 1.69E-03 | -5.653 | 1.210 | -4.673 | 2.97E-06 | 5.22E-04 |
| ENSG00000231312 | MAP4K3-DT | -0.504 | 0.129 | -3.919 | 8.88E-05 | 2.08E-03 | -1.243 | 0.268 | -4.645 | 3.40E-06 | 5.80E-04 |
| ENSG00000057593 | F7 | -1.747 | 0.461 | -3.790 | 1.51E-04 | 3.04E-03 | -4.807 | 1.037 | -4.636 | 3.56E-06 | 5.94E-04 |
| ENSG00000089199 | CHGB | -1.800 | 0.527 | -3.415 | 6.37E-04 | 8.10E-03 | -4.179 | 0.909 | -4.600 | 4.23E-06 | 6.80E-04 |
| ENSG00000100150 | DEPDC5 | 0.295 | 0.091 | 3.246 | 1.17E-03 | 0.012 | 0.729 | 0.159 | 4.596 | 4.32E-06 | 6.84E-04 |
| ENSG00000181143 | MUC16 | -1.731 | 0.560 | -3.093 | 1.98E-03 | 0.018 | -4.342 | 0.946 | -4.590 | 4.42E-06 | 6.95E-04 |
| ENSG00000198947 | DMD | -1.096 | 0.258 | -4.251 | 2.13E-05 | 7.48E-04 | -4.236 | 0.924 | -4.585 | 4.55E-06 | 7.10E-04 |
| ENSG00000100979 | PLTP | -0.934 | 0.248 | -3.769 | 1.64E-04 | 3.20E-03 | -3.544 | 0.775 | -4.572 | 4.83E-06 | 7.38E-04 |
| ENSG00000134769 | DTNA | -1.686 | 0.315 | -5.349 | 8.87E-08 | 1.26E-05 | -4.152 | 0.909 | -4.567 | 4.94E-06 | 7.49E-04 |
| ENSG00000177932 | ZNF354C | -0.657 | 0.247 | -2.657 | 7.88E-03 | 0.047 | -3.987 | 0.874 | -4.565 | 5.00E-06 | 7.56E-04 |
| ENSG00000185269 | NOTUM | -2.735 | 0.505 | -5.419 | 6.01E-08 | 9.26E-06 | -3.936 | 0.863 | -4.562 | 5.08E-06 | 7.61E-04 |
| ENSG00000196917 | HCAR1 | -2.002 | 0.508 | -3.939 | 8.19E-05 | 1.96E-03 | -4.596 | 1.031 | -4.457 | 8.32E-06 | 1.14E-03 |
| ENSG00000227533 | SLC2A1-AS1 | -1.201 | 0.292 | -4.110 | 3.95E-05 | 1.18E-03 | -2.437 | 0.548 | -4.449 | 8.63E-06 | 1.17E-03 |
| ENSG00000213839 | TMX2P1 | 0.401 | 0.129 | 3.109 | 1.88E-03 | 0.017 | 0.822 | 0.185 | 4.446 | 8.73E-06 | 1.17E-03 |
| ENSG00000180999 | C1orf105 | -2.138 | 0.409 | -5.234 | 1.66E-07 | 2.04E-05 | -3.741 | 0.842 | -4.443 | 8.85E-06 | 1.18E-03 |
| ENSG00000125352 | RNF113A | -0.357 | 0.116 | -3.078 | 2.08E-03 | 0.019 | -0.941 | 0.212 | -4.434 | 9.23E-06 | 1.20E-03 |
| ENSG00000185905 | C16orf54 | -1.044 | 0.249 | -4.191 | 2.78E-05 | 9.04E-04 | -4.330 | 0.981 | -4.416 | 1.00E-05 | 1.28E-03 |
| ENSG00000147206 | NXF3 | 1.946 | 0.548 | 3.550 | 3.85E-04 | 5.65E-03 | 3.933 | 0.893 | 4.406 | 1.05E-05 | 1.33E-03 |
| ENSG00000144619 | CNTN4 | -0.598 | 0.194 | -3.076 | 2.10E-03 | 0.019 | -5.718 | 1.302 | -4.390 | 1.13E-05 | 1.41E-03 |
| ENSG00000237515 | SHISA9 | -2.169 | 0.658 | -3.299 | 9.72E-04 | 0.011 | -3.825 | 0.872 | -4.385 | 1.16E-05 | 1.42E-03 |
| ENSG00000054793 | ATP9A | -0.738 | 0.154 | -4.798 | 1.61E-06 | 1.10E-04 | -1.867 | 0.430 | -4.345 | 1.39E-05 | 1.62E-03 |
| ENSG00000258754 | LINC01579 | -1.107 | 0.339 | -3.266 | 1.09E-03 | 0.012 | -3.803 | 0.881 | -4.317 | 1.58E-05 | 1.78E-03 |
| ENSG00000182648 | LINC01006 | -1.038 | 0.211 | -4.917 | 8.79E-07 | 7.03E-05 | -2.266 | 0.526 | -4.307 | 1.66E-05 | 1.86E-03 |
| ENSG00000168386 | FILIP1L | -0.374 | 0.132 | -2.835 | 4.58E-03 | 0.032 | -3.093 | 0.722 | -4.281 | 1.86E-05 | 2.02E-03 |
| ENSG00000214182 | PTMAP5 | 0.830 | 0.192 | 4.328 | 1.51E-05 | 5.83E-04 | 1.571 | 0.368 | 4.274 | 1.92E-05 | 2.07E-03 |
| ENSG00000139219 | COL2A1 | -2.047 | 0.500 | -4.097 | 4.18E-05 | 1.23E-03 | -3.926 | 0.922 | -4.257 | 2.07E-05 | 2.18E-03 |
| ENSG00000140873 | ADAMTS18 | -1.179 | 0.365 | -3.228 | 1.25E-03 | 0.013 | -5.331 | 1.253 | -4.253 | 2.11E-05 | 2.20E-03 |
| ENSG00000146013 | GFRA3 | -2.065 | 0.412 | -5.009 | 5.47E-07 | 5.00E-05 | -3.970 | 0.936 | -4.242 | 2.22E-05 | 2.27E-03 |
| ENSG00000183722 | LHFPL6 | -0.310 | 0.111 | -2.794 | 5.20E-03 | 0.035 | -3.337 | 0.794 | -4.204 | 2.62E-05 | 2.64E-03 |
| ENSG00000139211 | AMIGO2 | -1.135 | 0.207 | -5.478 | 4.30E-08 | 7.23E-06 | -3.101 | 0.739 | -4.198 | 2.69E-05 | 2.66E-03 |
| ENSG00000228784 | LINC00954 | -1.406 | 0.315 | -4.468 | 7.88E-06 | 3.72E-04 | -2.676 | 0.647 | -4.133 | 3.58E-05 | 3.22E-03 |
| ENSG00000159217 | IGF2BP1 | -3.432 | 0.609 | -5.634 | 1.76E-08 | 3.50E-06 | -3.587 | 0.875 | -4.101 | 4.12E-05 | 3.54E-03 |
| ENSG00000140632 | GLYR1 | -0.384 | 0.109 | -3.508 | 4.52E-04 | 6.36E-03 | -0.953 | 0.233 | -4.084 | 4.42E-05 | 3.73E-03 |
| ENSG00000198910 | L1CAM | -1.543 | 0.387 | -3.990 | 6.59E-05 | 1.71E-03 | -3.202 | 0.785 | -4.081 | 4.49E-05 | 3.78E-03 |
| ENSG00000126803 | HSPA2 | -1.337 | 0.230 | -5.822 | 5.80E-09 | 1.48E-06 | -2.146 | 0.527 | -4.076 | 4.58E-05 | 3.83E-03 |
| ENSG00000101040 | ZMYND8 | -0.645 | 0.126 | -5.136 | 2.81E-07 | 3.04E-05 | -1.067 | 0.263 | -4.055 | 5.01E-05 | 4.09E-03 |
| ENSG00000113361 | CDH6 | -0.539 | 0.162 | -3.323 | 8.90E-04 | 0.010 | -3.654 | 0.902 | -4.052 | 5.09E-05 | 4.10E-03 |
| ENSG00000175928 | LRRN1 | -1.296 | 0.462 | -2.806 | 5.02E-03 | 0.034 | -4.184 | 1.036 | -4.038 | 5.40E-05 | 4.32E-03 |
| ENSG00000162408 | NOL9 | 0.390 | 0.112 | 3.490 | 4.83E-04 | 6.63E-03 | 0.873 | 0.216 | 4.037 | 5.40E-05 | 4.32E-03 |
| ENSG00000180875 | GREM2 | -1.274 | 0.356 | -3.578 | 3.46E-04 | 5.22E-03 | -4.387 | 1.088 | -4.033 | 5.51E-05 | 4.36E-03 |
| ENSG00000137502 | RAB30 | -0.523 | 0.170 | -3.078 | 2.09E-03 | 0.019 | -1.598 | 0.399 | -4.008 | 6.13E-05 | 4.67E-03 |
| ENSG00000109158 | GABRA4 | -2.212 | 0.600 | -3.689 | 2.25E-04 | 3.96E-03 | -5.509 | 1.377 | -4.001 | 6.30E-05 | 4.73E-03 |
| ENSG00000113889 | KNG1 | -2.370 | 0.433 | -5.479 | 4.29E-08 | 7.23E-06 | -3.740 | 0.935 | -3.999 | 6.36E-05 | 4.75E-03 |
| ENSG00000114115 | RBP1 | -1.104 | 0.363 | -3.038 | 2.38E-03 | 0.020 | -4.460 | 1.120 | -3.980 | 6.89E-05 | 5.04E-03 |
| ENSG00000124155 | PIGT | -0.338 | 0.110 | -3.070 | 2.14E-03 | 0.019 | -0.939 | 0.238 | -3.942 | 8.08E-05 | 5.66E-03 |
| ENSG00000228742 | LINC02577 | -1.638 | 0.336 | -4.879 | 1.06E-06 | 8.21E-05 | -3.420 | 0.869 | -3.937 | 8.25E-05 | 5.76E-03 |
| ENSG00000183161 | FANCF | -0.523 | 0.112 | -4.653 | 3.28E-06 | 1.95E-04 | -0.869 | 0.221 | -3.930 | 8.49E-05 | 5.86E-03 |
| ENSG00000022277 | RTF2 | -0.315 | 0.099 | -3.178 | 1.48E-03 | 0.015 | -0.871 | 0.223 | -3.912 | 9.16E-05 | 6.19E-03 |
| ENSG00000145147 | SLIT2 | -0.880 | 0.268 | -3.279 | 1.04E-03 | 0.011 | -3.286 | 0.841 | -3.907 | 9.35E-05 | 6.27E-03 |
| ENSG00000204335 | SP5 | -0.895 | 0.338 | -2.647 | 8.12E-03 | 0.048 | -2.936 | 0.753 | -3.898 | 9.71E-05 | 6.47E-03 |
| ENSG00000154027 | AK5 | -1.131 | 0.336 | -3.366 | 7.63E-04 | 9.18E-03 | -3.294 | 0.845 | -3.896 | 9.79E-05 | 6.47E-03 |
| ENSG00000147041 | SYTL5 | -1.797 | 0.329 | -5.456 | 4.86E-08 | 7.82E-06 | -3.119 | 0.801 | -3.895 | 9.81E-05 | 6.47E-03 |
| ENSG00000169744 | LDB2 | -0.348 | 0.125 | -2.776 | 5.50E-03 | 0.037 | -4.689 | 1.206 | -3.887 | 1.01E-04 | 6.61E-03 |
| ENSG00000140093 | SERPINA10 | -2.279 | 0.547 | -4.164 | 3.13E-05 | 9.88E-04 | -4.538 | 1.170 | -3.880 | 1.04E-04 | 6.76E-03 |
| ENSG00000197837 | H4-16 | -0.720 | 0.199 | -3.625 | 2.89E-04 | 4.66E-03 | -1.554 | 0.402 | -3.868 | 1.10E-04 | 7.02E-03 |
| ENSG00000272953 |  | -1.017 | 0.245 | -4.159 | 3.20E-05 | 1.01E-03 | -1.550 | 0.401 | -3.867 | 1.10E-04 | 7.03E-03 |
| ENSG00000123560 | PLP1 | -1.470 | 0.437 | -3.368 | 7.56E-04 | 9.14E-03 | -3.037 | 0.788 | -3.856 | 1.15E-04 | 7.28E-03 |
| ENSG00000248429 | FAM198B-AS1 | -2.142 | 0.360 | -5.950 | 2.67E-09 | 8.32E-07 | -3.521 | 0.918 | -3.837 | 1.25E-04 | 7.73E-03 |
| ENSG00000149295 | DRD2 | -1.870 | 0.462 | -4.050 | 5.13E-05 | 1.42E-03 | -4.140 | 1.079 | -3.837 | 1.25E-04 | 7.73E-03 |
| ENSG00000167046 |  | -1.060 | 0.326 | -3.248 | 1.16E-03 | 0.012 | -1.651 | 0.431 | -3.831 | 1.28E-04 | 7.87E-03 |
| ENSG00000116329 | OPRD1 | -1.778 | 0.450 | -3.953 | 7.70E-05 | 1.90E-03 | -3.646 | 0.957 | -3.810 | 1.39E-04 | 8.38E-03 |
| ENSG00000130544 | ZNF557 | 0.298 | 0.099 | 3.011 | 2.61E-03 | 0.022 | 0.782 | 0.207 | 3.785 | 1.53E-04 | 8.97E-03 |
| ENSG00000183530 | PRR14L | 0.257 | 0.095 | 2.713 | 6.66E-03 | 0.042 | 0.770 | 0.204 | 3.772 | 1.62E-04 | 9.22E-03 |
| ENSG00000269696 |  | -0.966 | 0.302 | -3.202 | 1.36E-03 | 0.014 | -4.054 | 1.079 | -3.758 | 1.71E-04 | 0.010 |
| ENSG00000258837 |  | -2.265 | 0.439 | -5.164 | 2.42E-07 | 2.72E-05 | -4.022 | 1.070 | -3.757 | 1.72E-04 | 0.010 |
| ENSG00000062524 | LTK | 1.206 | 0.386 | 3.128 | 1.76E-03 | 0.017 | 3.155 | 0.840 | 3.756 | 1.73E-04 | 0.010 |
| ENSG00000149634 | SPATA25 | -1.267 | 0.231 | -5.497 | 3.86E-08 | 6.67E-06 | -1.396 | 0.375 | -3.723 | 1.97E-04 | 0.010 |
| ENSG00000117114 | ADGRL2 | -0.409 | 0.154 | -2.663 | 7.74E-03 | 0.047 | -2.541 | 0.684 | -3.713 | 2.05E-04 | 0.011 |
| ENSG00000157617 | C2CD2 | 0.272 | 0.098 | 2.785 | 5.35E-03 | 0.036 | 1.663 | 0.449 | 3.704 | 2.12E-04 | 0.011 |
| ENSG00000260604 |  | -2.430 | 0.423 | -5.744 | 9.25E-09 | 2.16E-06 | -2.854 | 0.771 | -3.701 | 2.15E-04 | 0.011 |
| ENSG00000131558 | EXOC4 | -0.337 | 0.086 | -3.933 | 8.39E-05 | 2.00E-03 | -0.894 | 0.242 | -3.688 | 2.26E-04 | 0.011 |
| ENSG00000104332 | SFRP1 | -1.054 | 0.374 | -2.819 | 4.81E-03 | 0.033 | -3.740 | 1.017 | -3.679 | 2.34E-04 | 0.012 |
| ENSG00000007933 | FMO3 | -0.827 | 0.236 | -3.499 | 4.67E-04 | 6.52E-03 | -4.186 | 1.148 | -3.646 | 2.66E-04 | 0.013 |
| ENSG00000173826 | KCNH6 | -1.271 | 0.437 | -2.910 | 3.61E-03 | 0.027 | -2.995 | 0.823 | -3.638 | 2.74E-04 | 0.013 |
| ENSG00000261716 | H2BC20P | -0.588 | 0.171 | -3.439 | 5.83E-04 | 7.63E-03 | -1.223 | 0.336 | -3.635 | 2.78E-04 | 0.013 |
| ENSG00000182224 | CYB5D1 | 0.652 | 0.157 | 4.149 | 3.34E-05 | 1.04E-03 | 0.901 | 0.249 | 3.612 | 3.04E-04 | 0.014 |
| ENSG00000140057 | AK7 | 0.694 | 0.224 | 3.093 | 1.98E-03 | 0.018 | 1.934 | 0.537 | 3.603 | 3.14E-04 | 0.014 |
| ENSG00000204217 | BMPR2 | -0.470 | 0.125 | -3.773 | 1.61E-04 | 3.17E-03 | -1.027 | 0.285 | -3.597 | 3.22E-04 | 0.014 |
| ENSG00000134627 | PIWIL4 | -0.960 | 0.182 | -5.270 | 1.37E-07 | 1.76E-05 | -1.700 | 0.474 | -3.587 | 3.34E-04 | 0.015 |
| ENSG00000135929 | CYP27A1 | -0.964 | 0.217 | -4.447 | 8.69E-06 | 4.02E-04 | -2.529 | 0.706 | -3.580 | 3.44E-04 | 0.015 |
| ENSG00000132801 | ZSWIM3 | -0.497 | 0.146 | -3.413 | 6.42E-04 | 8.14E-03 | -1.102 | 0.309 | -3.571 | 3.56E-04 | 0.015 |
| ENSG00000197273 | GUCA2A | -1.605 | 0.413 | -3.887 | 1.01E-04 | 2.29E-03 | -3.839 | 1.079 | -3.558 | 3.74E-04 | 0.016 |
| ENSG00000124479 | NDP | -1.330 | 0.437 | -3.047 | 2.31E-03 | 0.020 | -3.295 | 0.929 | -3.547 | 3.89E-04 | 0.016 |
| ENSG00000141664 | ZCCHC2 | 0.555 | 0.103 | 5.360 | 8.34E-08 | 1.21E-05 | 0.926 | 0.261 | 3.542 | 3.97E-04 | 0.016 |
| ENSG00000124226 | RNF114 | -0.308 | 0.091 | -3.384 | 7.14E-04 | 8.76E-03 | -0.736 | 0.208 | -3.540 | 4.01E-04 | 0.017 |
| ENSG00000235142 | LINC02532 | -1.279 | 0.364 | -3.516 | 4.38E-04 | 6.24E-03 | -2.749 | 0.781 | -3.522 | 4.29E-04 | 0.017 |
| ENSG00000071051 | NCK2 | -0.332 | 0.097 | -3.428 | 6.08E-04 | 7.84E-03 | -0.867 | 0.246 | -3.521 | 4.30E-04 | 0.017 |
| ENSG00000134398 | ERN2 | 0.576 | 0.215 | 2.675 | 7.47E-03 | 0.046 | 2.683 | 0.762 | 3.518 | 4.34E-04 | 0.017 |
| ENSG00000162738 | VANGL2 | -0.962 | 0.294 | -3.268 | 1.08E-03 | 0.012 | -2.994 | 0.851 | -3.516 | 4.37E-04 | 0.017 |
| ENSG00000171121 | KCNMB3 | -1.015 | 0.211 | -4.812 | 1.49E-06 | 1.05E-04 | -1.819 | 0.519 | -3.507 | 4.54E-04 | 0.018 |
| ENSG00000125966 | MMP24 | -0.913 | 0.233 | -3.916 | 9.01E-05 | 2.10E-03 | -1.955 | 0.559 | -3.499 | 4.68E-04 | 0.018 |
| ENSG00000124257 | NEURL2 | -1.020 | 0.188 | -5.419 | 6.00E-08 | 9.26E-06 | -1.268 | 0.363 | -3.493 | 4.78E-04 | 0.018 |
| ENSG00000227946 |  | -0.616 | 0.209 | -2.944 | 3.24E-03 | 0.025 | -0.822 | 0.236 | -3.479 | 5.03E-04 | 0.019 |
| ENSG00000144230 | GPR17 | -0.988 | 0.372 | -2.658 | 7.86E-03 | 0.047 | -3.170 | 0.912 | -3.476 | 5.09E-04 | 0.019 |
| ENSG00000183208 | GDPGP1 | 0.479 | 0.122 | 3.943 | 8.06E-05 | 1.94E-03 | 0.805 | 0.232 | 3.465 | 5.29E-04 | 0.020 |
| ENSG00000111052 | LIN7A | -2.101 | 0.390 | -5.383 | 7.32E-08 | 1.09E-05 | -2.983 | 0.862 | -3.461 | 5.38E-04 | 0.020 |
| ENSG00000142168 | SOD1 | 0.467 | 0.131 | 3.557 | 3.75E-04 | 5.55E-03 | 0.680 | 0.197 | 3.458 | 5.45E-04 | 0.020 |
| ENSG00000164266 | SPINK1 | -0.944 | 0.305 | -3.099 | 1.94E-03 | 0.018 | -3.319 | 0.961 | -3.453 | 5.53E-04 | 0.020 |
| ENSG00000256618 | MTRNR2L1 | -2.491 | 0.667 | -3.733 | 1.89E-04 | 3.50E-03 | -1.453 | 0.422 | -3.440 | 5.82E-04 | 0.021 |
| ENSG00000101337 | TM9SF4 | -0.280 | 0.106 | -2.650 | 8.05E-03 | 0.048 | -0.609 | 0.177 | -3.438 | 5.86E-04 | 0.021 |
| ENSG00000167244 | IGF2 | -3.459 | 0.611 | -5.664 | 1.48E-08 | 3.22E-06 | -3.595 | 1.046 | -3.437 | 5.87E-04 | 0.021 |
| ENSG00000240449 | REPIN1-AS1 | -0.962 | 0.190 | -5.073 | 3.91E-07 | 3.85E-05 | -2.291 | 0.666 | -3.437 | 5.88E-04 | 0.021 |
| ENSG00000011295 | TTC19 | 0.616 | 0.114 | 5.385 | 7.24E-08 | 1.09E-05 | 0.732 | 0.213 | 3.429 | 6.05E-04 | 0.022 |
| ENSG00000148734 | NPFFR1 | -1.301 | 0.284 | -4.582 | 4.61E-06 | 2.53E-04 | -2.719 | 0.793 | -3.427 | 6.09E-04 | 0.022 |
| ENSG00000165194 | PCDH19 | -1.233 | 0.417 | -2.954 | 3.14E-03 | 0.025 | -3.978 | 1.161 | -3.426 | 6.12E-04 | 0.022 |
| ENSG00000006652 | IFRD1 | -0.507 | 0.134 | -3.792 | 1.50E-04 | 3.03E-03 | -1.014 | 0.296 | -3.425 | 6.14E-04 | 0.022 |
| ENSG00000135452 | TSPAN31 | -0.452 | 0.101 | -4.459 | 8.22E-06 | 3.84E-04 | -0.831 | 0.244 | -3.410 | 6.50E-04 | 0.022 |
| ENSG00000183621 | ZNF438 | 0.275 | 0.099 | 2.786 | 5.34E-03 | 0.036 | 2.078 | 0.610 | 3.409 | 6.53E-04 | 0.022 |
| ENSG00000148120 | AOPEP | -0.370 | 0.139 | -2.656 | 7.90E-03 | 0.047 | -1.304 | 0.384 | -3.396 | 6.83E-04 | 0.023 |
| ENSG00000162641 | AKNAD1 | -1.483 | 0.289 | -5.131 | 2.88E-07 | 3.05E-05 | -1.911 | 0.563 | -3.394 | 6.89E-04 | 0.023 |
| ENSG00000088386 | SLC15A1 | -1.395 | 0.508 | -2.743 | 6.09E-03 | 0.039 | -4.035 | 1.190 | -3.392 | 6.94E-04 | 0.023 |
| ENSG00000230071 | RPL4P6 | 0.627 | 0.167 | 3.758 | 1.71E-04 | 3.29E-03 | 1.107 | 0.327 | 3.383 | 7.16E-04 | 0.024 |
| ENSG00000152705 | CATSPER3 | 0.559 | 0.173 | 3.225 | 1.26E-03 | 0.013 | 1.235 | 0.365 | 3.380 | 7.26E-04 | 0.024 |
| ENSG00000169764 | UGP2 | -0.360 | 0.116 | -3.092 | 1.99E-03 | 0.018 | -0.786 | 0.233 | -3.377 | 7.33E-04 | 0.024 |
| ENSG00000151366 | NDUFC2 | -0.673 | 0.116 | -5.793 | 6.93E-09 | 1.72E-06 | -0.919 | 0.272 | -3.374 | 7.40E-04 | 0.024 |
| ENSG00000159182 | PRAC1 | -2.698 | 0.793 | -3.403 | 6.67E-04 | 8.37E-03 | -6.728 | 2.003 | -3.359 | 7.81E-04 | 0.025 |
| ENSG00000183287 | CCBE1 | -0.726 | 0.274 | -2.645 | 8.18E-03 | 0.048 | -2.792 | 0.831 | -3.359 | 7.82E-04 | 0.025 |
| ENSG00000130294 | KIF1A | -2.053 | 0.448 | -4.580 | 4.64E-06 | 2.54E-04 | -2.057 | 0.613 | -3.356 | 7.92E-04 | 0.026 |
| ENSG00000077274 | CAPN6 | -2.138 | 0.533 | -4.015 | 5.95E-05 | 1.59E-03 | -4.241 | 1.267 | -3.347 | 8.17E-04 | 0.026 |
| ENSG00000267508 | ZNF285 | -1.354 | 0.403 | -3.356 | 7.91E-04 | 9.43E-03 | -3.060 | 0.916 | -3.341 | 8.35E-04 | 0.027 |
| ENSG00000175445 | LPL | -1.397 | 0.310 | -4.500 | 6.79E-06 | 3.37E-04 | -3.315 | 0.993 | -3.337 | 8.45E-04 | 0.027 |
| ENSG00000198597 | ZNF536 | -1.114 | 0.360 | -3.097 | 1.96E-03 | 0.018 | -4.367 | 1.313 | -3.325 | 8.85E-04 | 0.028 |
| ENSG00000272341 |  | -0.708 | 0.193 | -3.664 | 2.48E-04 | 4.20E-03 | -1.464 | 0.441 | -3.321 | 8.98E-04 | 0.028 |
| ENSG00000249306 | LINC01411 | -2.306 | 0.602 | -3.833 | 1.27E-04 | 2.69E-03 | -2.221 | 0.670 | -3.316 | 9.15E-04 | 0.028 |
| ENSG00000261324 |  | -0.814 | 0.219 | -3.719 | 2.00E-04 | 3.65E-03 | -1.463 | 0.442 | -3.307 | 9.43E-04 | 0.029 |
| ENSG00000115084 | SLC35F5 | -0.418 | 0.140 | -2.992 | 2.77E-03 | 0.023 | -0.839 | 0.254 | -3.302 | 9.60E-04 | 0.029 |
| ENSG00000253626 | EIF5AL1 | 0.979 | 0.332 | 2.946 | 3.22E-03 | 0.025 | 1.309 | 0.397 | 3.301 | 9.62E-04 | 0.029 |
| ENSG00000162419 | GMEB1 | 0.238 | 0.070 | 3.385 | 7.12E-04 | 8.75E-03 | 0.628 | 0.191 | 3.292 | 9.95E-04 | 0.030 |
| ENSG00000229931 | ATXN1-AS1 | -0.812 | 0.202 | -4.020 | 5.81E-05 | 1.57E-03 | -1.352 | 0.411 | -3.289 | 1.01E-03 | 0.030 |
| ENSG00000172817 | CYP7B1 | -0.567 | 0.202 | -2.806 | 5.01E-03 | 0.034 | -4.170 | 1.269 | -3.284 | 1.02E-03 | 0.031 |
| ENSG00000158161 | EYA3 | 0.266 | 0.096 | 2.763 | 5.73E-03 | 0.038 | 0.607 | 0.185 | 3.283 | 1.03E-03 | 0.031 |
| ENSG00000131067 | GGT7 | -0.772 | 0.221 | -3.493 | 4.77E-04 | 6.59E-03 | -1.914 | 0.583 | -3.283 | 1.03E-03 | 0.031 |
| ENSG00000147027 | TMEM47 | -0.597 | 0.206 | -2.902 | 3.71E-03 | 0.028 | -3.562 | 1.087 | -3.275 | 1.05E-03 | 0.031 |
| ENSG00000158296 | SLC13A3 | -1.958 | 0.452 | -4.335 | 1.46E-05 | 5.70E-04 | -2.266 | 0.693 | -3.271 | 1.07E-03 | 0.031 |
| ENSG00000049769 | PPP1R3F | -0.552 | 0.210 | -2.632 | 8.48E-03 | 0.050 | -1.347 | 0.412 | -3.267 | 1.09E-03 | 0.032 |
| ENSG00000133107 | TRPC4 | -0.655 | 0.198 | -3.302 | 9.59E-04 | 0.011 | -3.311 | 1.016 | -3.259 | 1.12E-03 | 0.032 |
| ENSG00000125726 | CD70 | 0.797 | 0.298 | 2.679 | 7.39E-03 | 0.045 | 2.828 | 0.868 | 3.257 | 1.13E-03 | 0.032 |
| ENSG00000130988 | RGN | -1.970 | 0.443 | -4.444 | 8.81E-06 | 4.05E-04 | -3.165 | 0.972 | -3.256 | 1.13E-03 | 0.032 |
| ENSG00000153132 | CLGN | -1.528 | 0.449 | -3.401 | 6.72E-04 | 8.42E-03 | -2.948 | 0.906 | -3.254 | 1.14E-03 | 0.032 |
| ENSG00000144647 | POMGNT2 | 0.567 | 0.148 | 3.823 | 1.32E-04 | 2.78E-03 | 0.789 | 0.243 | 3.253 | 1.14E-03 | 0.032 |
| ENSG00000106415 | GLCCI1 | -0.855 | 0.153 | -5.571 | 2.54E-08 | 4.81E-06 | -1.364 | 0.419 | -3.252 | 1.14E-03 | 0.033 |
| ENSG00000124177 | CHD6 | -0.478 | 0.120 | -3.998 | 6.39E-05 | 1.67E-03 | -0.687 | 0.212 | -3.243 | 1.18E-03 | 0.033 |
| ENSG00000172728 | FUT10 | 0.493 | 0.145 | 3.391 | 6.96E-04 | 8.61E-03 | 0.774 | 0.239 | 3.232 | 1.23E-03 | 0.034 |
| ENSG00000170927 | PKHD1 | -1.512 | 0.455 | -3.323 | 8.89E-04 | 0.010 | -2.635 | 0.816 | -3.229 | 1.24E-03 | 0.034 |
| ENSG00000231292 | IGKV1OR2-108 | -1.229 | 0.385 | -3.191 | 1.42E-03 | 0.014 | -2.547 | 0.791 | -3.221 | 1.28E-03 | 0.035 |
| ENSG00000105472 | CLEC11A | 0.597 | 0.160 | 3.718 | 2.01E-04 | 3.66E-03 | 2.275 | 0.706 | 3.220 | 1.28E-03 | 0.035 |
| ENSG00000151729 | SLC25A4 | 0.474 | 0.122 | 3.884 | 1.03E-04 | 2.31E-03 | 0.651 | 0.202 | 3.218 | 1.29E-03 | 0.035 |
| ENSG00000100083 | GGA1 | 0.298 | 0.094 | 3.180 | 1.47E-03 | 0.015 | 0.469 | 0.146 | 3.212 | 1.32E-03 | 0.035 |
| ENSG00000168672 | LRATD2 | -0.475 | 0.144 | -3.301 | 9.62E-04 | 0.011 | -1.973 | 0.615 | -3.210 | 1.33E-03 | 0.036 |
| ENSG00000044524 | EPHA3 | -0.910 | 0.226 | -4.026 | 5.66E-05 | 1.54E-03 | -2.641 | 0.823 | -3.208 | 1.34E-03 | 0.036 |
| ENSG00000124164 | VAPB | -0.334 | 0.099 | -3.387 | 7.05E-04 | 8.69E-03 | -0.917 | 0.286 | -3.207 | 1.34E-03 | 0.036 |
| ENSG00000259456 | ADNP-AS1 | -0.454 | 0.152 | -2.983 | 2.86E-03 | 0.023 | -0.918 | 0.286 | -3.206 | 1.35E-03 | 0.036 |
| ENSG00000108590 | MED31 | 0.476 | 0.127 | 3.737 | 1.86E-04 | 3.46E-03 | 0.868 | 0.272 | 3.191 | 1.42E-03 | 0.037 |
| ENSG00000120054 | CPN1 | -2.539 | 0.606 | -4.189 | 2.80E-05 | 9.09E-04 | -3.602 | 1.129 | -3.190 | 1.42E-03 | 0.037 |
| ENSG00000197183 | NOL4L | -0.322 | 0.121 | -2.659 | 7.84E-03 | 0.047 | -1.140 | 0.359 | -3.178 | 1.48E-03 | 0.038 |
| ENSG00000139209 | SLC38A4 | -1.763 | 0.399 | -4.416 | 1.00E-05 | 4.42E-04 | -2.854 | 0.898 | -3.178 | 1.49E-03 | 0.038 |
| ENSG00000178821 | TMEM52 | 0.736 | 0.235 | 3.130 | 1.75E-03 | 0.017 | 1.345 | 0.424 | 3.175 | 1.50E-03 | 0.038 |
| ENSG00000183864 | TOB2 | 0.225 | 0.085 | 2.647 | 8.11E-03 | 0.048 | 0.439 | 0.138 | 3.174 | 1.50E-03 | 0.038 |
| ENSG00000184903 | IMMP2L | -0.514 | 0.135 | -3.799 | 1.45E-04 | 2.95E-03 | -0.882 | 0.279 | -3.156 | 1.60E-03 | 0.040 |
| ENSG00000180861 | LINC01559 | -1.345 | 0.258 | -5.214 | 1.85E-07 | 2.20E-05 | -2.748 | 0.873 | -3.147 | 1.65E-03 | 0.041 |
| ENSG00000100395 | L3MBTL2 | 0.261 | 0.081 | 3.235 | 1.22E-03 | 0.013 | 0.568 | 0.181 | 3.139 | 1.70E-03 | 0.041 |
| ENSG00000106069 | CHN2 | -1.119 | 0.233 | -4.797 | 1.61E-06 | 1.10E-04 | -1.867 | 0.596 | -3.131 | 1.74E-03 | 0.042 |
| ENSG00000124222 | STX16 | -0.538 | 0.119 | -4.533 | 5.82E-06 | 2.98E-04 | -0.761 | 0.243 | -3.129 | 1.76E-03 | 0.042 |
| ENSG00000112195 | TREML2 | -1.131 | 0.427 | -2.647 | 8.12E-03 | 0.048 | -3.147 | 1.006 | -3.127 | 1.77E-03 | 0.042 |
| ENSG00000260032 | NORAD | -0.488 | 0.108 | -4.519 | 6.20E-06 | 3.14E-04 | -0.749 | 0.240 | -3.121 | 1.80E-03 | 0.043 |
| ENSG00000212694 | LINC01089 | -0.946 | 0.179 | -5.275 | 1.32E-07 | 1.74E-05 | -1.076 | 0.345 | -3.113 | 1.85E-03 | 0.044 |
| ENSG00000249859 | PVT1 | -0.676 | 0.159 | -4.242 | 2.22E-05 | 7.65E-04 | -2.049 | 0.660 | -3.106 | 1.90E-03 | 0.044 |
| ENSG00000118514 | ALDH8A1 | 1.053 | 0.341 | 3.089 | 2.01E-03 | 0.018 | 1.905 | 0.614 | 3.102 | 1.92E-03 | 0.044 |
| ENSG00000257512 |  | -1.102 | 0.209 | -5.284 | 1.26E-07 | 1.70E-05 | -2.071 | 0.668 | -3.101 | 1.93E-03 | 0.045 |
| ENSG00000178971 | CTC1 | 0.463 | 0.114 | 4.071 | 4.67E-05 | 1.33E-03 | 0.620 | 0.200 | 3.101 | 1.93E-03 | 0.045 |
| ENSG00000100162 | CENPM | 0.466 | 0.173 | 2.693 | 7.08E-03 | 0.044 | 0.724 | 0.234 | 3.099 | 1.94E-03 | 0.045 |
| ENSG00000222033 | LINC01124 | -1.054 | 0.306 | -3.446 | 5.69E-04 | 7.52E-03 | -2.371 | 0.766 | -3.096 | 1.96E-03 | 0.045 |
| ENSG00000177352 | CCDC71 | 0.377 | 0.123 | 3.067 | 2.16E-03 | 0.019 | 0.512 | 0.166 | 3.096 | 1.96E-03 | 0.045 |
| ENSG00000101367 | MAPRE1 | -0.392 | 0.109 | -3.607 | 3.09E-04 | 4.86E-03 | -0.574 | 0.186 | -3.090 | 2.00E-03 | 0.046 |
| ENSG00000114248 | LRRC31 | -0.939 | 0.238 | -3.945 | 7.97E-05 | 1.93E-03 | -2.607 | 0.846 | -3.082 | 2.06E-03 | 0.047 |
| ENSG00000132824 | SERINC3 | -0.353 | 0.110 | -3.208 | 1.33E-03 | 0.014 | -0.624 | 0.202 | -3.081 | 2.06E-03 | 0.047 |
| ENSG00000173083 | HPSE | 0.642 | 0.162 | 3.957 | 7.59E-05 | 1.88E-03 | 1.560 | 0.507 | 3.077 | 2.09E-03 | 0.047 |
| ENSG00000166913 | YWHAB | -0.487 | 0.104 | -4.666 | 3.08E-06 | 1.85E-04 | -0.643 | 0.209 | -3.074 | 2.11E-03 | 0.048 |
| ENSG00000251322 | SHANK3 | 0.415 | 0.136 | 3.052 | 2.27E-03 | 0.020 | 1.753 | 0.571 | 3.069 | 2.15E-03 | 0.048 |
| ENSG00000106615 | RHEB | -0.479 | 0.107 | -4.483 | 7.35E-06 | 3.56E-04 | -0.578 | 0.189 | -3.066 | 2.17E-03 | 0.048 |
| ENSG00000115310 | RTN4 | -0.349 | 0.088 | -3.980 | 6.91E-05 | 1.77E-03 | -0.607 | 0.198 | -3.063 | 2.19E-03 | 0.049 |
| ENSG00000187079 | TEAD1 | -0.471 | 0.119 | -3.971 | 7.16E-05 | 1.79E-03 | -0.793 | 0.260 | -3.055 | 2.25E-03 | 0.050 |
| ENSG00000104435 | STMN2 | -1.175 | 0.366 | -3.209 | 1.33E-03 | 0.014 | -3.502 | 1.150 | -3.046 | 2.32E-03 | 0.051 |
| ENSG00000087495 | PHACTR3 | -1.560 | 0.361 | -4.326 | 1.52E-05 | 5.88E-04 | -2.630 | 0.865 | -3.041 | 2.36E-03 | 0.051 |
| ENSG00000185652 | NTF3 | 0.800 | 0.260 | 3.072 | 2.12E-03 | 0.019 | 2.999 | 0.986 | 3.040 | 2.37E-03 | 0.051 |
| ENSG00000172361 | CFAP53 | 0.826 | 0.206 | 4.012 | 6.02E-05 | 1.60E-03 | 1.654 | 0.544 | 3.039 | 2.37E-03 | 0.051 |
| ENSG00000100294 | MCAT | 0.466 | 0.111 | 4.208 | 2.58E-05 | 8.52E-04 | 0.627 | 0.207 | 3.033 | 2.42E-03 | 0.052 |
| ENSG00000100413 | POLR3H | 0.380 | 0.109 | 3.478 | 5.05E-04 | 6.84E-03 | 0.557 | 0.184 | 3.029 | 2.45E-03 | 0.052 |
| ENSG00000232119 | MCTS1 | -0.359 | 0.106 | -3.375 | 7.38E-04 | 8.98E-03 | -0.581 | 0.192 | -3.024 | 2.49E-03 | 0.053 |
| ENSG00000258738 |  | -0.594 | 0.156 | -3.807 | 1.40E-04 | 2.90E-03 | -0.861 | 0.285 | -3.020 | 2.53E-03 | 0.053 |
| ENSG00000212993 | POU5F1B | -2.461 | 0.294 | -8.362 | 6.18E-17 | 2.31E-13 | -2.494 | 0.827 | -3.015 | 2.57E-03 | 0.054 |
| ENSG00000223551 | TMSB4XP4 | -0.952 | 0.282 | -3.369 | 7.54E-04 | 9.12E-03 | -1.119 | 0.373 | -3.000 | 2.70E-03 | 0.056 |
| ENSG00000181773 | GPR3 | 0.754 | 0.217 | 3.480 | 5.01E-04 | 6.82E-03 | 1.222 | 0.408 | 2.996 | 2.74E-03 | 0.056 |
| ENSG00000133083 | DCLK1 | -0.685 | 0.236 | -2.909 | 3.62E-03 | 0.027 | -2.760 | 0.923 | -2.991 | 2.78E-03 | 0.057 |
| ENSG00000237499 | WAKMAR2 | -0.783 | 0.161 | -4.870 | 1.11E-06 | 8.45E-05 | -1.905 | 0.637 | -2.990 | 2.79E-03 | 0.057 |
| ENSG00000103707 | MTFMT | 0.316 | 0.088 | 3.602 | 3.16E-04 | 4.93E-03 | 0.574 | 0.192 | 2.983 | 2.86E-03 | 0.058 |
| ENSG00000132382 | MYBBP1A | 0.383 | 0.127 | 3.024 | 2.49E-03 | 0.021 | 0.646 | 0.217 | 2.981 | 2.88E-03 | 0.058 |
| ENSG00000224078 | SNHG14 | -1.136 | 0.335 | -3.391 | 6.96E-04 | 8.61E-03 | -2.829 | 0.953 | -2.970 | 2.98E-03 | 0.059 |
| ENSG00000101146 | RAE1 | -0.411 | 0.101 | -4.056 | 4.98E-05 | 1.40E-03 | -0.625 | 0.211 | -2.963 | 3.05E-03 | 0.060 |
| ENSG00000146054 | TRIM7 | 2.258 | 0.319 | 7.080 | 1.44E-12 | 1.92E-09 | 1.551 | 0.524 | 2.962 | 3.06E-03 | 0.060 |
| ENSG00000162929 | KIAA1841 | -0.849 | 0.119 | -7.147 | 8.85E-13 | 1.38E-09 | -0.673 | 0.227 | -2.958 | 3.09E-03 | 0.061 |
| ENSG00000118873 | RAB3GAP2 | -0.400 | 0.104 | -3.865 | 1.11E-04 | 2.44E-03 | -0.539 | 0.182 | -2.955 | 3.13E-03 | 0.061 |
| ENSG00000139679 | LPAR6 | -0.813 | 0.223 | -3.647 | 2.65E-04 | 4.38E-03 | -2.164 | 0.733 | -2.953 | 3.14E-03 | 0.061 |
| ENSG00000066032 | CTNNA2 | -1.902 | 0.596 | -3.192 | 1.41E-03 | 0.014 | -2.966 | 1.005 | -2.952 | 3.15E-03 | 0.061 |
| ENSG00000127863 | TNFRSF19 | -2.116 | 0.408 | -5.187 | 2.13E-07 | 2.49E-05 | -2.488 | 0.843 | -2.952 | 3.16E-03 | 0.061 |
| ENSG00000130830 | MPP1 | -0.724 | 0.218 | -3.319 | 9.05E-04 | 0.010 | -2.017 | 0.684 | -2.948 | 3.20E-03 | 0.061 |
| ENSG00000189298 | ZKSCAN3 | 0.361 | 0.108 | 3.326 | 8.83E-04 | 0.010 | 0.790 | 0.268 | 2.945 | 3.23E-03 | 0.062 |
| ENSG00000124783 | SSR1 | 0.320 | 0.099 | 3.244 | 1.18E-03 | 0.012 | 0.611 | 0.208 | 2.944 | 3.24E-03 | 0.062 |
| ENSG00000116819 | TFAP2E | 0.793 | 0.295 | 2.689 | 7.18E-03 | 0.044 | 1.453 | 0.494 | 2.941 | 3.27E-03 | 0.062 |
| ENSG00000110274 | CEP164 | -0.278 | 0.102 | -2.737 | 6.20E-03 | 0.040 | -0.618 | 0.210 | -2.939 | 3.29E-03 | 0.062 |
| ENSG00000242622 |  | -0.847 | 0.167 | -5.071 | 3.95E-07 | 3.86E-05 | -1.009 | 0.344 | -2.933 | 3.36E-03 | 0.063 |
| ENSG00000169710 | FASN | 0.457 | 0.168 | 2.712 | 6.68E-03 | 0.042 | 0.796 | 0.272 | 2.932 | 3.37E-03 | 0.063 |
| ENSG00000145824 | CXCL14 | -0.936 | 0.312 | -3.001 | 2.69E-03 | 0.022 | -3.390 | 1.159 | -2.926 | 3.44E-03 | 0.064 |
| ENSG00000249464 | LINC01091 | -1.125 | 0.250 | -4.494 | 6.97E-06 | 3.43E-04 | -2.514 | 0.859 | -2.925 | 3.44E-03 | 0.064 |
| ENSG00000186472 | PCLO | -0.897 | 0.332 | -2.700 | 6.93E-03 | 0.043 | -2.094 | 0.716 | -2.925 | 3.45E-03 | 0.064 |
| ENSG00000168924 | LETM1 | 0.496 | 0.107 | 4.631 | 3.63E-06 | 2.09E-04 | 0.580 | 0.199 | 2.913 | 3.58E-03 | 0.065 |
| ENSG00000077380 | DYNC1I2 | -0.285 | 0.085 | -3.364 | 7.67E-04 | 9.22E-03 | -0.619 | 0.213 | -2.910 | 3.62E-03 | 0.066 |
| ENSG00000108244 | KRT23 | -3.201 | 0.451 | -7.105 | 1.21E-12 | 1.73E-09 | -2.934 | 1.011 | -2.903 | 3.70E-03 | 0.067 |
| ENSG00000173598 | NUDT4 | -0.449 | 0.151 | -2.980 | 2.89E-03 | 0.023 | -0.893 | 0.308 | -2.900 | 3.73E-03 | 0.067 |
| ENSG00000121741 | ZMYM2 | -0.451 | 0.138 | -3.275 | 1.06E-03 | 0.012 | -0.855 | 0.295 | -2.900 | 3.73E-03 | 0.067 |
| ENSG00000168065 | SLC22A11 | -1.792 | 0.411 | -4.362 | 1.29E-05 | 5.24E-04 | -2.150 | 0.742 | -2.898 | 3.76E-03 | 0.068 |
| ENSG00000273230 |  | -0.631 | 0.221 | -2.852 | 4.34E-03 | 0.031 | -1.112 | 0.385 | -2.890 | 3.85E-03 | 0.068 |
| ENSG00000196724 | ZNF418 | -0.765 | 0.267 | -2.868 | 4.13E-03 | 0.030 | -2.194 | 0.759 | -2.890 | 3.85E-03 | 0.068 |
| ENSG00000168876 | ANKRD49 | -0.389 | 0.109 | -3.575 | 3.50E-04 | 5.26E-03 | -0.766 | 0.265 | -2.887 | 3.89E-03 | 0.069 |
| ENSG00000269609 | RPARP-AS1 | 0.325 | 0.118 | 2.757 | 5.83E-03 | 0.038 | 0.776 | 0.269 | 2.884 | 3.93E-03 | 0.069 |
| ENSG00000245937 | LINC01184 | -0.662 | 0.115 | -5.736 | 9.71E-09 | 2.24E-06 | -1.327 | 0.461 | -2.880 | 3.97E-03 | 0.070 |
| ENSG00000075461 | CACNG4 | -2.992 | 0.526 | -5.692 | 1.26E-08 | 2.82E-06 | -2.515 | 0.873 | -2.880 | 3.98E-03 | 0.070 |
| ENSG00000183011 | NAA38 | 0.602 | 0.170 | 3.553 | 3.81E-04 | 5.61E-03 | 0.837 | 0.291 | 2.878 | 4.00E-03 | 0.070 |
| ENSG00000170638 | TRABD | 0.608 | 0.144 | 4.215 | 2.50E-05 | 8.34E-04 | 0.548 | 0.191 | 2.875 | 4.05E-03 | 0.070 |
| ENSG00000084764 | MAPRE3 | -0.722 | 0.181 | -3.981 | 6.87E-05 | 1.76E-03 | -1.197 | 0.417 | -2.874 | 4.05E-03 | 0.071 |
| ENSG00000230561 | CCDC192 | -1.190 | 0.226 | -5.268 | 1.38E-07 | 1.76E-05 | -2.011 | 0.700 | -2.872 | 4.08E-03 | 0.071 |
| ENSG00000155287 | SLC25A28 | 0.343 | 0.105 | 3.280 | 1.04E-03 | 0.011 | 0.403 | 0.140 | 2.871 | 4.09E-03 | 0.071 |
| ENSG00000080189 | SLC35C2 | -0.271 | 0.097 | -2.810 | 4.96E-03 | 0.034 | -0.568 | 0.198 | -2.868 | 4.13E-03 | 0.071 |
| ENSG00000101126 | ADNP | -0.347 | 0.090 | -3.868 | 1.10E-04 | 2.41E-03 | -0.442 | 0.155 | -2.856 | 4.28E-03 | 0.073 |
| ENSG00000085840 | ORC1 | 0.360 | 0.130 | 2.775 | 5.51E-03 | 0.037 | 0.729 | 0.256 | 2.844 | 4.46E-03 | 0.075 |
| ENSG00000112039 | FANCE | 0.290 | 0.110 | 2.643 | 8.22E-03 | 0.048 | 0.515 | 0.182 | 2.838 | 4.54E-03 | 0.076 |
| ENSG00000215041 | NEURL4 | 0.429 | 0.130 | 3.315 | 9.18E-04 | 0.010 | 0.443 | 0.156 | 2.835 | 4.59E-03 | 0.076 |
| ENSG00000139055 | ERP27 | -1.422 | 0.379 | -3.748 | 1.78E-04 | 3.39E-03 | -2.325 | 0.821 | -2.833 | 4.61E-03 | 0.077 |
| ENSG00000184956 | MUC6 | 5.249 | 0.548 | 9.571 | 1.06E-21 | 1.98E-17 | 2.561 | 0.905 | 2.829 | 4.67E-03 | 0.077 |
| ENSG00000241224 | C3orf85 | -1.587 | 0.344 | -4.618 | 3.88E-06 | 2.20E-04 | -3.018 | 1.068 | -2.826 | 4.71E-03 | 0.078 |
| ENSG00000197928 | ZNF677 | -0.482 | 0.182 | -2.646 | 8.14E-03 | 0.048 | -2.113 | 0.749 | -2.823 | 4.76E-03 | 0.078 |
| ENSG00000169621 | APLF | -0.762 | 0.187 | -4.074 | 4.62E-05 | 1.33E-03 | -1.133 | 0.402 | -2.820 | 4.80E-03 | 0.079 |
| ENSG00000169962 | TAS1R3 | -1.314 | 0.358 | -3.669 | 2.43E-04 | 4.14E-03 | -1.542 | 0.547 | -2.819 | 4.82E-03 | 0.079 |
| ENSG00000132825 | PPP1R3D | -0.577 | 0.132 | -4.354 | 1.34E-05 | 5.33E-04 | -0.843 | 0.299 | -2.819 | 4.82E-03 | 0.079 |
| ENSG00000015676 | NUDCD3 | -0.332 | 0.096 | -3.467 | 5.26E-04 | 7.06E-03 | -0.553 | 0.196 | -2.819 | 4.82E-03 | 0.079 |
| ENSG00000237686 |  | 0.692 | 0.262 | 2.640 | 8.29E-03 | 0.049 | 1.865 | 0.664 | 2.810 | 4.96E-03 | 0.080 |
| ENSG00000229344 | MTCO2P12 | -2.166 | 0.410 | -5.285 | 1.26E-07 | 1.70E-05 | -0.906 | 0.323 | -2.809 | 4.97E-03 | 0.080 |
| ENSG00000235655 | H3P6 | -1.054 | 0.233 | -4.524 | 6.05E-06 | 3.09E-04 | -0.648 | 0.231 | -2.807 | 5.00E-03 | 0.080 |
| ENSG00000240541 | TM4SF1-AS1 | -0.975 | 0.278 | -3.509 | 4.50E-04 | 6.34E-03 | -1.949 | 0.696 | -2.801 | 5.10E-03 | 0.081 |
| ENSG00000114200 | BCHE | -1.127 | 0.370 | -3.050 | 2.29E-03 | 0.020 | -2.669 | 0.955 | -2.795 | 5.19E-03 | 0.082 |
| ENSG00000154328 | NEIL2 | 0.478 | 0.155 | 3.072 | 2.12E-03 | 0.019 | 0.861 | 0.308 | 2.793 | 5.22E-03 | 0.083 |
| ENSG00000247400 | DNAJC3-DT | -0.760 | 0.194 | -3.918 | 8.94E-05 | 2.09E-03 | -1.122 | 0.402 | -2.790 | 5.27E-03 | 0.083 |
| ENSG00000148935 | GAS2 | -1.463 | 0.282 | -5.193 | 2.07E-07 | 2.42E-05 | -2.092 | 0.753 | -2.780 | 5.44E-03 | 0.084 |
| ENSG00000272732 |  | -0.737 | 0.219 | -3.361 | 7.77E-04 | 9.30E-03 | -1.293 | 0.465 | -2.779 | 5.46E-03 | 0.085 |
| ENSG00000115514 | TXNDC9 | -0.835 | 0.137 | -6.088 | 1.15E-09 | 4.55E-07 | -0.647 | 0.233 | -2.777 | 5.48E-03 | 0.085 |
| ENSG00000100147 | CCDC134 | 0.521 | 0.127 | 4.095 | 4.23E-05 | 1.24E-03 | 0.654 | 0.236 | 2.774 | 5.54E-03 | 0.085 |
| ENSG00000142920 | AZIN2 | 0.575 | 0.201 | 2.861 | 4.23E-03 | 0.030 | 1.281 | 0.462 | 2.770 | 5.60E-03 | 0.086 |
| ENSG00000198134 | PTMAP9 | 1.036 | 0.186 | 5.570 | 2.55E-08 | 4.81E-06 | 0.808 | 0.292 | 2.767 | 5.65E-03 | 0.086 |
| ENSG00000267199 |  | -1.233 | 0.332 | -3.715 | 2.03E-04 | 3.68E-03 | -1.346 | 0.488 | -2.759 | 5.79E-03 | 0.087 |
| ENSG00000196167 | COLCA1 | -1.878 | 0.389 | -4.825 | 1.40E-06 | 1.00E-04 | -2.012 | 0.730 | -2.758 | 5.82E-03 | 0.087 |
| ENSG00000130699 | TAF4 | -0.423 | 0.105 | -4.044 | 5.25E-05 | 1.45E-03 | -0.621 | 0.225 | -2.756 | 5.85E-03 | 0.088 |
| ENSG00000104213 | PDGFRL | 0.880 | 0.235 | 3.747 | 1.79E-04 | 3.40E-03 | 1.636 | 0.594 | 2.753 | 5.91E-03 | 0.088 |
| ENSG00000205643 | CDPF1 | 0.448 | 0.105 | 4.277 | 1.90E-05 | 6.89E-04 | 0.617 | 0.224 | 2.751 | 5.95E-03 | 0.089 |
| ENSG00000235669 | CHN2-AS1 | -1.840 | 0.428 | -4.301 | 1.70E-05 | 6.29E-04 | -3.607 | 1.314 | -2.745 | 6.05E-03 | 0.090 |
| ENSG00000197016 | ZNF470 | -1.009 | 0.249 | -4.051 | 5.11E-05 | 1.42E-03 | -2.396 | 0.874 | -2.743 | 6.09E-03 | 0.090 |
| ENSG00000083720 | OXCT1 | 0.590 | 0.215 | 2.743 | 6.08E-03 | 0.039 | 1.591 | 0.581 | 2.737 | 6.21E-03 | 0.091 |
| ENSG00000226824 |  | -0.544 | 0.186 | -2.924 | 3.45E-03 | 0.026 | -1.264 | 0.462 | -2.735 | 6.24E-03 | 0.091 |
| ENSG00000178015 | GPR150 | -1.454 | 0.353 | -4.120 | 3.79E-05 | 1.14E-03 | -2.127 | 0.781 | -2.724 | 6.45E-03 | 0.093 |
| ENSG00000143344 | RGL1 | -0.434 | 0.152 | -2.851 | 4.36E-03 | 0.031 | -1.721 | 0.632 | -2.722 | 6.48E-03 | 0.093 |
| ENSG00000231252 |  | -1.966 | 0.357 | -5.502 | 3.75E-08 | 6.53E-06 | -3.195 | 1.176 | -2.716 | 6.60E-03 | 0.094 |
| ENSG00000039600 | SOX30 | -1.007 | 0.304 | -3.311 | 9.30E-04 | 0.011 | -2.303 | 0.852 | -2.704 | 6.84E-03 | 0.096 |
| ENSG00000178397 | FAM220A | -0.311 | 0.093 | -3.344 | 8.27E-04 | 0.010 | -0.604 | 0.223 | -2.701 | 6.92E-03 | 0.097 |
| ENSG00000272894 |  | -0.614 | 0.184 | -3.345 | 8.24E-04 | 0.010 | -1.190 | 0.441 | -2.698 | 6.98E-03 | 0.097 |
| ENSG00000061656 | SPAG4 | -0.593 | 0.199 | -2.983 | 2.85E-03 | 0.023 | -1.317 | 0.488 | -2.697 | 6.99E-03 | 0.097 |
| ENSG00000266402 | SNHG25 | 1.235 | 0.399 | 3.094 | 1.98E-03 | 0.018 | 0.880 | 0.328 | 2.685 | 7.26E-03 | 0.099 |
| ENSG00000101193 | GID8 | -0.258 | 0.088 | -2.919 | 3.51E-03 | 0.027 | -0.585 | 0.218 | -2.681 | 7.34E-03 | 0.100 |
| ENSG00000260992 | DOCK9-DT | -0.576 | 0.211 | -2.731 | 6.32E-03 | 0.040 | -1.301 | 0.486 | -2.680 | 7.37E-03 | 0.100 |
| ENSG00000237517 | DGCR5 | -1.017 | 0.379 | -2.681 | 7.34E-03 | 0.045 | -2.176 | 0.812 | -2.679 | 7.38E-03 | 0.100 |
| ENSG00000234456 | MAGI2-AS3 | -0.411 | 0.155 | -2.655 | 7.92E-03 | 0.047 | -2.860 | 1.067 | -2.679 | 7.38E-03 | 0.100 |
| ENSG00000128739 | SNRPN | -1.070 | 0.295 | -3.627 | 2.86E-04 | 4.64E-03 | -3.121 | 1.170 | -2.666 | 7.67E-03 | 0.103 |
| ENSG00000114503 | NCBP2 | -0.348 | 0.103 | -3.364 | 7.69E-04 | 9.24E-03 | -0.496 | 0.186 | -2.663 | 7.74E-03 | 0.103 |
| ENSG00000132970 | WASF3 | -1.026 | 0.336 | -3.056 | 2.25E-03 | 0.019 | -2.700 | 1.015 | -2.661 | 7.80E-03 | 0.104 |
| ENSG00000104915 | STX10 | 0.308 | 0.116 | 2.650 | 8.05E-03 | 0.048 | 0.531 | 0.200 | 2.652 | 8.01E-03 | 0.106 |
| ENSG00000197557 | TTC30A | -0.901 | 0.166 | -5.437 | 5.41E-08 | 8.47E-06 | -1.042 | 0.393 | -2.652 | 8.01E-03 | 0.106 |
| ENSG00000066926 | FECH | 0.746 | 0.123 | 6.053 | 1.42E-09 | 5.27E-07 | 0.960 | 0.362 | 2.651 | 8.03E-03 | 0.106 |
| ENSG00000100505 | TRIM9 | -1.432 | 0.356 | -4.028 | 5.61E-05 | 1.54E-03 | -1.892 | 0.716 | -2.645 | 8.17E-03 | 0.107 |
| ENSG00000166526 | ZNF3 | -0.341 | 0.094 | -3.610 | 3.07E-04 | 4.83E-03 | -0.499 | 0.189 | -2.645 | 8.17E-03 | 0.107 |
| ENSG00000082212 | ME2 | 0.461 | 0.137 | 3.371 | 7.50E-04 | 9.09E-03 | 0.787 | 0.298 | 2.645 | 8.17E-03 | 0.107 |
| ENSG00000168772 | CXXC4 | -1.228 | 0.287 | -4.285 | 1.83E-05 | 6.66E-04 | -2.418 | 0.914 | -2.644 | 8.19E-03 | 0.107 |
| ENSG00000163328 | GPR155 | -1.011 | 0.281 | -3.591 | 3.29E-04 | 5.03E-03 | -2.135 | 0.808 | -2.642 | 8.24E-03 | 0.107 |
| ENSG00000136261 | BZW2 | -0.292 | 0.103 | -2.841 | 4.50E-03 | 0.032 | -0.537 | 0.205 | -2.624 | 8.70E-03 | 0.110 |
| ENSG00000154832 | CXXC1 | 0.436 | 0.118 | 3.702 | 2.14E-04 | 3.81E-03 | 0.703 | 0.268 | 2.620 | 8.80E-03 | 0.111 |
| ENSG00000115828 | QPCT | -1.288 | 0.286 | -4.508 | 6.54E-06 | 3.28E-04 | -1.935 | 0.740 | -2.616 | 8.90E-03 | 0.112 |
| ENSG00000120694 | HSPH1 | -0.979 | 0.157 | -6.222 | 4.90E-10 | 2.18E-07 | -0.785 | 0.301 | -2.608 | 9.10E-03 | 0.114 |
| ENSG00000167034 | NKX3-1 | 1.655 | 0.297 | 5.575 | 2.47E-08 | 4.76E-06 | 1.929 | 0.741 | 2.604 | 9.21E-03 | 0.114 |
| ENSG00000197408 | CYP2B6 | -1.319 | 0.319 | -4.142 | 3.44E-05 | 1.06E-03 | -3.092 | 1.187 | -2.604 | 9.21E-03 | 0.114 |
| ENSG00000167536 | DHRS13 | 0.576 | 0.154 | 3.741 | 1.83E-04 | 3.44E-03 | 0.804 | 0.309 | 2.602 | 9.28E-03 | 0.115 |
| ENSG00000110318 | CEP126 | -0.788 | 0.230 | -3.427 | 6.11E-04 | 7.86E-03 | -1.400 | 0.539 | -2.599 | 9.34E-03 | 0.115 |
| ENSG00000025708 | TYMP | 0.530 | 0.176 | 3.008 | 2.63E-03 | 0.022 | 1.495 | 0.575 | 2.599 | 9.35E-03 | 0.115 |
| ENSG00000128203 | ASPHD2 | 1.210 | 0.177 | 6.837 | 8.10E-12 | 8.17E-09 | 1.482 | 0.570 | 2.599 | 9.36E-03 | 0.115 |
| ENSG00000164825 | DEFB1 | -1.238 | 0.393 | -3.152 | 1.62E-03 | 0.016 | -2.561 | 0.986 | -2.598 | 9.37E-03 | 0.115 |
| ENSG00000149658 | YTHDF1 | -0.296 | 0.086 | -3.457 | 5.47E-04 | 7.31E-03 | -0.610 | 0.235 | -2.598 | 9.37E-03 | 0.115 |
| ENSG00000013297 | CLDN11 | -0.887 | 0.278 | -3.191 | 1.42E-03 | 0.014 | -2.263 | 0.873 | -2.591 | 0.010 | 0.117 |
| ENSG00000188419 | CHM | -0.375 | 0.126 | -2.981 | 2.87E-03 | 0.023 | -0.489 | 0.189 | -2.591 | 0.010 | 0.117 |
| ENSG00000156831 | NSMCE2 | -0.646 | 0.134 | -4.841 | 1.29E-06 | 9.51E-05 | -1.256 | 0.485 | -2.587 | 0.010 | 0.117 |
| ENSG00000174175 | SELP | -0.806 | 0.283 | -2.844 | 4.46E-03 | 0.032 | -2.604 | 1.007 | -2.586 | 0.010 | 0.118 |
| ENSG00000157625 | TAB3 | -0.353 | 0.125 | -2.818 | 4.84E-03 | 0.033 | -0.777 | 0.302 | -2.573 | 0.010 | 0.120 |
| ENSG00000020922 | MRE11 | -0.741 | 0.112 | -6.598 | 4.18E-11 | 2.88E-08 | -0.624 | 0.243 | -2.572 | 0.010 | 0.120 |
| ENSG00000054118 | THRAP3 | 0.248 | 0.072 | 3.434 | 5.96E-04 | 7.72E-03 | 0.411 | 0.160 | 2.570 | 0.010 | 0.120 |
| ENSG00000162676 | GFI1 | 0.589 | 0.209 | 2.817 | 4.85E-03 | 0.033 | 1.464 | 0.571 | 2.567 | 0.010 | 0.121 |
| ENSG00000088340 | FER1L4 | -1.293 | 0.322 | -4.017 | 5.90E-05 | 1.58E-03 | -1.797 | 0.701 | -2.562 | 0.010 | 0.122 |
| ENSG00000152291 | TGOLN2 | -0.359 | 0.111 | -3.226 | 1.26E-03 | 0.013 | -0.570 | 0.223 | -2.558 | 0.011 | 0.123 |
| ENSG00000198133 | TMEM229B | 0.660 | 0.190 | 3.472 | 5.16E-04 | 6.96E-03 | 1.550 | 0.607 | 2.556 | 0.011 | 0.124 |
| ENSG00000148541 | FAM13C | -0.670 | 0.216 | -3.105 | 1.90E-03 | 0.017 | -2.668 | 1.044 | -2.555 | 0.011 | 0.124 |
| ENSG00000179761 | PIPOX | -1.181 | 0.383 | -3.086 | 2.03E-03 | 0.018 | -1.294 | 0.507 | -2.551 | 0.011 | 0.124 |
| ENSG00000254726 | MEX3A | -0.978 | 0.234 | -4.174 | 3.00E-05 | 9.56E-04 | -1.236 | 0.486 | -2.546 | 0.011 | 0.125 |
| ENSG00000137710 | RDX | -0.646 | 0.225 | -2.872 | 4.08E-03 | 0.030 | -1.667 | 0.655 | -2.545 | 0.011 | 0.126 |
| ENSG00000203880 | PCMTD2 | -0.416 | 0.150 | -2.769 | 5.63E-03 | 0.037 | -0.861 | 0.338 | -2.544 | 0.011 | 0.126 |
| ENSG00000246582 |  | 0.439 | 0.155 | 2.828 | 4.69E-03 | 0.033 | 0.889 | 0.350 | 2.542 | 0.011 | 0.126 |
| ENSG00000173227 | SYT12 | 1.399 | 0.325 | 4.306 | 1.66E-05 | 6.18E-04 | 1.958 | 0.772 | 2.535 | 0.011 | 0.127 |
| ENSG00000123561 | SERPINA7 | -3.013 | 0.586 | -5.146 | 2.67E-07 | 2.94E-05 | -5.165 | 2.038 | -2.535 | 0.011 | 0.127 |
| ENSG00000092847 | AGO1 | 0.281 | 0.100 | 2.804 | 5.04E-03 | 0.034 | 0.525 | 0.207 | 2.533 | 0.011 | 0.128 |
| ENSG00000206535 | LNP1 | -0.609 | 0.178 | -3.422 | 6.21E-04 | 7.94E-03 | -1.103 | 0.435 | -2.533 | 0.011 | 0.128 |
| ENSG00000237361 | TUSC8 | -1.157 | 0.377 | -3.066 | 2.17E-03 | 0.019 | -2.613 | 1.032 | -2.532 | 0.011 | 0.128 |
| ENSG00000204070 | SYS1 | -0.290 | 0.109 | -2.665 | 7.70E-03 | 0.047 | -0.631 | 0.249 | -2.531 | 0.011 | 0.128 |
| ENSG00000164761 | TNFRSF11B | -1.080 | 0.296 | -3.646 | 2.67E-04 | 4.39E-03 | -2.192 | 0.866 | -2.530 | 0.011 | 0.128 |
| ENSG00000122420 | PTGFR | -0.706 | 0.248 | -2.840 | 4.51E-03 | 0.032 | -2.744 | 1.085 | -2.529 | 0.011 | 0.128 |
| ENSG00000225345 | SNX18P3 | 1.175 | 0.289 | 4.060 | 4.92E-05 | 1.38E-03 | 1.613 | 0.638 | 2.526 | 0.012 | 0.129 |
| ENSG00000260742 |  | -0.831 | 0.203 | -4.088 | 4.35E-05 | 1.26E-03 | -1.519 | 0.601 | -2.526 | 0.012 | 0.129 |
| ENSG00000145681 | HAPLN1 | -1.040 | 0.279 | -3.726 | 1.95E-04 | 3.58E-03 | -2.603 | 1.031 | -2.524 | 0.012 | 0.129 |
| ENSG00000111678 | C12orf57 | 0.569 | 0.175 | 3.252 | 1.14E-03 | 0.012 | 0.814 | 0.323 | 2.522 | 0.012 | 0.130 |
| ENSG00000109466 | KLHL2 | 0.303 | 0.105 | 2.875 | 4.04E-03 | 0.029 | 0.522 | 0.208 | 2.517 | 0.012 | 0.131 |
| ENSG00000257883 |  | -1.523 | 0.536 | -2.840 | 4.51E-03 | 0.032 | -2.846 | 1.131 | -2.517 | 0.012 | 0.131 |
| ENSG00000152767 | FARP1 | -0.812 | 0.181 | -4.494 | 6.99E-06 | 3.43E-04 | -1.523 | 0.608 | -2.505 | 0.012 | 0.134 |
| ENSG00000006744 | ELAC2 | 0.472 | 0.105 | 4.494 | 6.99E-06 | 3.43E-04 | 0.475 | 0.190 | 2.500 | 0.012 | 0.135 |
| ENSG00000184203 | PPP1R2 | -0.420 | 0.115 | -3.661 | 2.51E-04 | 4.21E-03 | -0.627 | 0.251 | -2.498 | 0.013 | 0.135 |
| ENSG00000173530 | TNFRSF10D | 0.601 | 0.202 | 2.968 | 2.99E-03 | 0.024 | 1.090 | 0.437 | 2.494 | 0.013 | 0.136 |
| ENSG00000163513 | TGFBR2 | -0.989 | 0.127 | -7.767 | 8.01E-15 | 2.49E-11 | -1.228 | 0.492 | -2.494 | 0.013 | 0.136 |
| ENSG00000234390 | USP27X-AS1 | -0.796 | 0.198 | -4.019 | 5.85E-05 | 1.57E-03 | -1.165 | 0.468 | -2.490 | 0.013 | 0.137 |
| ENSG00000158480 | SPATA2 | -0.404 | 0.102 | -3.945 | 7.97E-05 | 1.93E-03 | -0.549 | 0.221 | -2.487 | 0.013 | 0.137 |
| ENSG00000106443 | PHF14 | -0.443 | 0.100 | -4.421 | 9.82E-06 | 4.36E-04 | -0.658 | 0.265 | -2.484 | 0.013 | 0.138 |
| ENSG00000272173 |  | 0.519 | 0.190 | 2.738 | 6.18E-03 | 0.040 | 0.829 | 0.334 | 2.482 | 0.013 | 0.139 |
| ENSG00000151413 | NUBPL | -0.883 | 0.130 | -6.780 | 1.20E-11 | 1.07E-08 | -0.621 | 0.252 | -2.469 | 0.014 | 0.141 |
| ENSG00000136205 | TNS3 | -0.343 | 0.101 | -3.381 | 7.21E-04 | 8.83E-03 | -1.214 | 0.492 | -2.466 | 0.014 | 0.142 |
| ENSG00000131389 | SLC6A6 | -0.830 | 0.184 | -4.509 | 6.51E-06 | 3.27E-04 | -0.929 | 0.378 | -2.459 | 0.014 | 0.144 |
| ENSG00000241258 | CRCP | -0.568 | 0.084 | -6.787 | 1.15E-11 | 1.07E-08 | -0.470 | 0.191 | -2.459 | 0.014 | 0.144 |
| ENSG00000116017 | ARID3A | -0.793 | 0.211 | -3.760 | 1.70E-04 | 3.27E-03 | -1.017 | 0.414 | -2.458 | 0.014 | 0.144 |
| ENSG00000108561 | C1QBP | 0.512 | 0.139 | 3.676 | 2.37E-04 | 4.08E-03 | 0.609 | 0.248 | 2.457 | 0.014 | 0.144 |
| ENSG00000187398 | LUZP2 | -1.439 | 0.378 | -3.812 | 1.38E-04 | 2.86E-03 | -3.097 | 1.260 | -2.457 | 0.014 | 0.144 |
| ENSG00000135679 | MDM2 | 0.494 | 0.142 | 3.476 | 5.09E-04 | 6.87E-03 | 0.903 | 0.367 | 2.457 | 0.014 | 0.144 |
| ENSG00000272636 | DOC2B | 1.093 | 0.332 | 3.293 | 9.91E-04 | 0.011 | 2.699 | 1.099 | 2.457 | 0.014 | 0.144 |
| ENSG00000265399 |  | -0.588 | 0.219 | -2.692 | 7.10E-03 | 0.044 | -0.851 | 0.346 | -2.455 | 0.014 | 0.144 |
| ENSG00000106565 | TMEM176B | -0.748 | 0.201 | -3.724 | 1.96E-04 | 3.60E-03 | -2.205 | 0.898 | -2.455 | 0.014 | 0.144 |
| ENSG00000153904 | DDAH1 | 0.334 | 0.114 | 2.937 | 3.31E-03 | 0.026 | 0.907 | 0.369 | 2.454 | 0.014 | 0.145 |
| ENSG00000134440 | NARS1 | 0.353 | 0.089 | 3.976 | 7.02E-05 | 1.77E-03 | 0.634 | 0.258 | 2.454 | 0.014 | 0.145 |
| ENSG00000124215 | CDH26 | -1.229 | 0.349 | -3.517 | 4.36E-04 | 6.22E-03 | -1.527 | 0.622 | -2.454 | 0.014 | 0.145 |
| ENSG00000213981 |  | -2.687 | 0.508 | -5.287 | 1.24E-07 | 1.69E-05 | -2.777 | 1.135 | -2.447 | 0.014 | 0.146 |
| ENSG00000150687 | PRSS23 | -0.900 | 0.196 | -4.600 | 4.23E-06 | 2.37E-04 | -1.623 | 0.663 | -2.447 | 0.014 | 0.146 |
| ENSG00000188706 | ZDHHC9 | -0.331 | 0.118 | -2.809 | 4.96E-03 | 0.034 | -0.554 | 0.226 | -2.447 | 0.014 | 0.146 |
| ENSG00000122490 | SLC66A2 | 0.707 | 0.138 | 5.133 | 2.85E-07 | 3.04E-05 | 0.611 | 0.250 | 2.446 | 0.014 | 0.146 |
| ENSG00000204390 | HSPA1L | -0.377 | 0.143 | -2.640 | 8.28E-03 | 0.049 | -0.793 | 0.325 | -2.442 | 0.015 | 0.147 |
| ENSG00000170577 | SIX2 | -2.164 | 0.502 | -4.316 | 1.59E-05 | 6.03E-04 | -2.554 | 1.047 | -2.440 | 0.015 | 0.147 |
| ENSG00000135740 | SLC9A5 | 0.532 | 0.201 | 2.645 | 8.17E-03 | 0.048 | 0.942 | 0.387 | 2.437 | 0.015 | 0.148 |
| ENSG00000105982 | RNF32 | -0.626 | 0.166 | -3.778 | 1.58E-04 | 3.15E-03 | -0.740 | 0.304 | -2.437 | 0.015 | 0.148 |
| ENSG00000167680 | SEMA6B | 0.566 | 0.152 | 3.727 | 1.94E-04 | 3.57E-03 | 1.479 | 0.607 | 2.437 | 0.015 | 0.148 |
| ENSG00000091972 | CD200 | 0.687 | 0.259 | 2.654 | 7.96E-03 | 0.048 | 2.477 | 1.018 | 2.433 | 0.015 | 0.149 |
| ENSG00000130035 | GALNT8 | -1.055 | 0.314 | -3.360 | 7.81E-04 | 9.34E-03 | -1.788 | 0.735 | -2.432 | 0.015 | 0.149 |
| ENSG00000130856 | ZNF236 | 0.261 | 0.095 | 2.761 | 5.77E-03 | 0.038 | 0.472 | 0.194 | 2.431 | 0.015 | 0.150 |
| ENSG00000005483 | KMT2E | -0.287 | 0.108 | -2.666 | 7.69E-03 | 0.047 | -0.446 | 0.184 | -2.430 | 0.015 | 0.150 |
| ENSG00000196335 | STK31 | -1.201 | 0.329 | -3.651 | 2.61E-04 | 4.34E-03 | -1.880 | 0.774 | -2.430 | 0.015 | 0.150 |
| ENSG00000126562 | WNK4 | -0.776 | 0.211 | -3.678 | 2.35E-04 | 4.06E-03 | -1.147 | 0.473 | -2.427 | 0.015 | 0.150 |
| ENSG00000104044 | OCA2 | -1.845 | 0.509 | -3.622 | 2.92E-04 | 4.68E-03 | -2.448 | 1.010 | -2.424 | 0.015 | 0.151 |
| ENSG00000258655 | ARHGAP5-AS1 | -0.842 | 0.169 | -4.966 | 6.82E-07 | 5.81E-05 | -0.963 | 0.398 | -2.422 | 0.015 | 0.151 |
| ENSG00000229180 |  | -0.559 | 0.154 | -3.634 | 2.79E-04 | 4.56E-03 | -0.705 | 0.291 | -2.421 | 0.015 | 0.152 |
| ENSG00000127928 | GNGT1 | -2.126 | 0.642 | -3.312 | 9.26E-04 | 0.011 | -2.764 | 1.144 | -2.416 | 0.016 | 0.153 |
| ENSG00000075336 | TIMM21 | 0.309 | 0.108 | 2.854 | 4.32E-03 | 0.031 | 0.661 | 0.274 | 2.415 | 0.016 | 0.153 |
| ENSG00000147655 | RSPO2 | -2.373 | 0.402 | -5.911 | 3.41E-09 | 9.78E-07 | -2.404 | 0.997 | -2.412 | 0.016 | 0.154 |
| ENSG00000103472 | RRN3P2 | -0.959 | 0.229 | -4.191 | 2.78E-05 | 9.04E-04 | -1.287 | 0.535 | -2.406 | 0.016 | 0.155 |
| ENSG00000272849 |  | -0.949 | 0.204 | -4.643 | 3.44E-06 | 2.03E-04 | -0.885 | 0.368 | -2.406 | 0.016 | 0.155 |
| ENSG00000175899 | A2M | -0.382 | 0.129 | -2.973 | 2.95E-03 | 0.024 | -1.514 | 0.631 | -2.398 | 0.016 | 0.157 |
| ENSG00000112902 | SEMA5A | -0.887 | 0.202 | -4.397 | 1.10E-05 | 4.71E-04 | -2.065 | 0.864 | -2.391 | 0.017 | 0.159 |
| ENSG00000185630 | PBX1 | -0.997 | 0.195 | -5.121 | 3.04E-07 | 3.17E-05 | -1.442 | 0.605 | -2.385 | 0.017 | 0.161 |
| ENSG00000166529 | ZSCAN21 | -0.326 | 0.099 | -3.292 | 9.96E-04 | 0.011 | -0.526 | 0.221 | -2.383 | 0.017 | 0.161 |
| ENSG00000244687 | UBE2V1 | -0.378 | 0.132 | -2.862 | 4.21E-03 | 0.030 | -0.453 | 0.190 | -2.382 | 0.017 | 0.161 |
| ENSG00000272821 |  | 0.431 | 0.153 | 2.818 | 4.83E-03 | 0.033 | 0.614 | 0.258 | 2.378 | 0.017 | 0.163 |
| ENSG00000105991 | HOXA1 | -0.764 | 0.237 | -3.226 | 1.25E-03 | 0.013 | -1.240 | 0.522 | -2.377 | 0.017 | 0.163 |
| ENSG00000203739 | PRDX6-AS1 | -0.652 | 0.180 | -3.618 | 2.97E-04 | 4.73E-03 | -0.969 | 0.409 | -2.371 | 0.018 | 0.164 |
| ENSG00000185156 | MFSD6L | 0.795 | 0.290 | 2.741 | 6.13E-03 | 0.040 | 2.182 | 0.920 | 2.371 | 0.018 | 0.164 |
| ENSG00000106178 | CCL24 | -1.157 | 0.312 | -3.713 | 2.05E-04 | 3.69E-03 | -2.518 | 1.064 | -2.366 | 0.018 | 0.165 |
| ENSG00000173221 | GLRX | 0.531 | 0.191 | 2.780 | 5.44E-03 | 0.036 | 1.403 | 0.593 | 2.366 | 0.018 | 0.165 |
| ENSG00000253669 | GASAL1 | -0.652 | 0.199 | -3.277 | 1.05E-03 | 0.011 | -1.027 | 0.437 | -2.352 | 0.019 | 0.168 |
| ENSG00000007952 | NOX1 | -1.303 | 0.275 | -4.734 | 2.20E-06 | 1.41E-04 | -1.947 | 0.828 | -2.351 | 0.019 | 0.169 |
| ENSG00000269887 |  | -0.872 | 0.267 | -3.269 | 1.08E-03 | 0.012 | -1.380 | 0.588 | -2.345 | 0.019 | 0.170 |
| ENSG00000249395 | CASC9 | -1.578 | 0.341 | -4.632 | 3.62E-06 | 2.09E-04 | -1.549 | 0.661 | -2.344 | 0.019 | 0.171 |
| ENSG00000170471 | RALGAPB | -0.411 | 0.105 | -3.928 | 8.56E-05 | 2.02E-03 | -0.500 | 0.214 | -2.343 | 0.019 | 0.171 |
| ENSG00000101470 | TNNC2 | -1.989 | 0.346 | -5.754 | 8.73E-09 | 2.12E-06 | -1.687 | 0.721 | -2.341 | 0.019 | 0.171 |
| ENSG00000139160 | ETFBKMT | 0.597 | 0.129 | 4.629 | 3.68E-06 | 2.10E-04 | 0.686 | 0.293 | 2.340 | 0.019 | 0.171 |
| ENSG00000132153 | DHX30 | 0.279 | 0.105 | 2.662 | 7.76E-03 | 0.047 | 0.354 | 0.152 | 2.331 | 0.020 | 0.174 |
| ENSG00000197971 | MBP | 0.576 | 0.111 | 5.164 | 2.42E-07 | 2.72E-05 | 0.760 | 0.326 | 2.330 | 0.020 | 0.174 |
| ENSG00000025293 | PHF20 | -0.371 | 0.093 | -3.977 | 6.98E-05 | 1.77E-03 | -0.487 | 0.209 | -2.329 | 0.020 | 0.174 |
| ENSG00000173334 | TRIB1 | -0.538 | 0.169 | -3.188 | 1.43E-03 | 0.014 | -1.332 | 0.572 | -2.327 | 0.020 | 0.174 |
| ENSG00000099949 | LZTR1 | 0.301 | 0.113 | 2.667 | 7.65E-03 | 0.046 | 0.523 | 0.225 | 2.327 | 0.020 | 0.175 |
| ENSG00000101162 | TUBB1 | -0.737 | 0.248 | -2.971 | 2.96E-03 | 0.024 | -0.947 | 0.407 | -2.327 | 0.020 | 0.175 |
| ENSG00000075945 | KIFAP3 | -0.386 | 0.131 | -2.946 | 3.21E-03 | 0.025 | -0.668 | 0.287 | -2.325 | 0.020 | 0.175 |
| ENSG00000165617 | DACT1 | -0.658 | 0.205 | -3.205 | 1.35E-03 | 0.014 | -1.950 | 0.840 | -2.321 | 0.020 | 0.176 |
| ENSG00000143878 | RHOB | -0.511 | 0.188 | -2.714 | 6.64E-03 | 0.042 | -0.997 | 0.430 | -2.320 | 0.020 | 0.176 |
| ENSG00000204054 | LINC00963 | -0.350 | 0.121 | -2.896 | 3.78E-03 | 0.028 | -0.688 | 0.297 | -2.318 | 0.020 | 0.177 |
| ENSG00000158352 | SHROOM4 | -0.900 | 0.178 | -5.044 | 4.56E-07 | 4.28E-05 | -2.357 | 1.020 | -2.311 | 0.021 | 0.178 |
| ENSG00000104177 | MYEF2 | -1.361 | 0.354 | -3.847 | 1.20E-04 | 2.57E-03 | -1.944 | 0.842 | -2.309 | 0.021 | 0.179 |
| ENSG00000229939 |  | -1.195 | 0.268 | -4.467 | 7.93E-06 | 3.72E-04 | -0.781 | 0.339 | -2.303 | 0.021 | 0.181 |
| ENSG00000068489 | PRR11 | -0.445 | 0.121 | -3.680 | 2.33E-04 | 4.04E-03 | -0.463 | 0.201 | -2.301 | 0.021 | 0.181 |
| ENSG00000168913 | ENHO | 1.267 | 0.336 | 3.775 | 1.60E-04 | 3.17E-03 | 1.574 | 0.684 | 2.301 | 0.021 | 0.181 |
| ENSG00000101958 | GLRA2 | -1.856 | 0.554 | -3.352 | 8.04E-04 | 0.010 | -2.620 | 1.139 | -2.300 | 0.021 | 0.181 |
| ENSG00000176771 | NCKAP5 | -1.450 | 0.334 | -4.347 | 1.38E-05 | 5.46E-04 | -2.559 | 1.114 | -2.298 | 0.022 | 0.182 |
| ENSG00000115896 | PLCL1 | -0.776 | 0.186 | -4.179 | 2.93E-05 | 9.42E-04 | -2.125 | 0.925 | -2.297 | 0.022 | 0.182 |
| ENSG00000136603 | SKIL | -0.697 | 0.179 | -3.899 | 9.67E-05 | 2.22E-03 | -0.951 | 0.414 | -2.296 | 0.022 | 0.182 |
| ENSG00000106038 | EVX1 | -1.443 | 0.443 | -3.256 | 1.13E-03 | 0.012 | -2.278 | 0.993 | -2.294 | 0.022 | 0.183 |
| ENSG00000261455 | LINC01003 | -0.578 | 0.171 | -3.377 | 7.33E-04 | 8.95E-03 | -0.830 | 0.362 | -2.294 | 0.022 | 0.183 |
| ENSG00000122557 | HERPUD2 | -0.347 | 0.087 | -3.978 | 6.94E-05 | 1.77E-03 | -0.563 | 0.245 | -2.294 | 0.022 | 0.183 |
| ENSG00000139433 | GLTP | 0.241 | 0.088 | 2.732 | 6.30E-03 | 0.040 | 0.529 | 0.231 | 2.290 | 0.022 | 0.184 |
| ENSG00000146411 | SLC2A12 | -1.135 | 0.305 | -3.718 | 2.01E-04 | 3.66E-03 | -1.462 | 0.638 | -2.290 | 0.022 | 0.184 |
| ENSG00000215912 | TTC34 | -0.781 | 0.265 | -2.944 | 3.24E-03 | 0.025 | -1.350 | 0.590 | -2.288 | 0.022 | 0.185 |
| ENSG00000196659 | TTC30B | -0.507 | 0.122 | -4.137 | 3.52E-05 | 1.08E-03 | -0.733 | 0.321 | -2.286 | 0.022 | 0.185 |
| ENSG00000141759 | TXNL4A | 0.421 | 0.105 | 4.018 | 5.87E-05 | 1.58E-03 | 0.508 | 0.224 | 2.273 | 0.023 | 0.189 |
| ENSG00000169057 | MECP2 | -0.266 | 0.092 | -2.887 | 3.89E-03 | 0.029 | -0.432 | 0.190 | -2.273 | 0.023 | 0.189 |
| ENSG00000187566 | NHLRC1 | -0.878 | 0.295 | -2.979 | 2.89E-03 | 0.023 | -1.734 | 0.763 | -2.273 | 0.023 | 0.189 |
| ENSG00000107105 | ELAVL2 | -2.300 | 0.506 | -4.545 | 5.50E-06 | 2.89E-04 | -2.443 | 1.075 | -2.272 | 0.023 | 0.189 |
| ENSG00000137440 | FGFBP1 | 0.992 | 0.319 | 3.107 | 1.89E-03 | 0.017 | 1.702 | 0.749 | 2.272 | 0.023 | 0.189 |
| ENSG00000135917 | SLC19A3 | -1.301 | 0.333 | -3.906 | 9.39E-05 | 2.17E-03 | -2.194 | 0.967 | -2.268 | 0.023 | 0.190 |
| ENSG00000119899 | SLC17A5 | 0.328 | 0.116 | 2.837 | 4.55E-03 | 0.032 | 0.620 | 0.273 | 2.268 | 0.023 | 0.190 |
| ENSG00000116194 | ANGPTL1 | -1.032 | 0.324 | -3.181 | 1.47E-03 | 0.015 | -1.718 | 0.758 | -2.266 | 0.023 | 0.190 |
| ENSG00000141002 | TCF25 | 0.382 | 0.096 | 3.974 | 7.07E-05 | 1.78E-03 | 0.398 | 0.176 | 2.264 | 0.024 | 0.191 |
| ENSG00000081913 | PHLPP1 | 0.415 | 0.118 | 3.512 | 4.45E-04 | 6.31E-03 | 0.609 | 0.269 | 2.263 | 0.024 | 0.191 |
| ENSG00000104549 | SQLE | -0.526 | 0.192 | -2.747 | 6.01E-03 | 0.039 | -1.086 | 0.480 | -2.262 | 0.024 | 0.191 |
| ENSG00000126003 | PLAGL2 | -0.754 | 0.149 | -5.073 | 3.92E-07 | 3.85E-05 | -0.587 | 0.260 | -2.259 | 0.024 | 0.192 |
| ENSG00000109063 | MYH3 | 0.796 | 0.228 | 3.495 | 4.73E-04 | 6.58E-03 | 1.107 | 0.490 | 2.257 | 0.024 | 0.193 |
| ENSG00000198718 | TOGARAM1 | -0.610 | 0.155 | -3.928 | 8.57E-05 | 2.02E-03 | -0.914 | 0.406 | -2.254 | 0.024 | 0.194 |
| ENSG00000156110 | ADK | -0.429 | 0.114 | -3.769 | 1.64E-04 | 3.20E-03 | -0.431 | 0.192 | -2.253 | 0.024 | 0.194 |
| ENSG00000092931 | MFSD11 | -0.283 | 0.096 | -2.933 | 3.36E-03 | 0.026 | -0.431 | 0.192 | -2.251 | 0.024 | 0.195 |
| ENSG00000099937 | SERPIND1 | -1.585 | 0.525 | -3.019 | 2.54E-03 | 0.021 | -1.742 | 0.774 | -2.250 | 0.024 | 0.195 |
| ENSG00000076258 | FMO4 | -0.636 | 0.164 | -3.877 | 1.06E-04 | 2.35E-03 | -0.969 | 0.431 | -2.247 | 0.025 | 0.196 |
| ENSG00000023839 | ABCC2 | -1.333 | 0.389 | -3.424 | 6.17E-04 | 7.92E-03 | -1.915 | 0.853 | -2.245 | 0.025 | 0.196 |
| ENSG00000196227 | FAM217B | -0.544 | 0.154 | -3.545 | 3.92E-04 | 5.74E-03 | -0.642 | 0.286 | -2.245 | 0.025 | 0.196 |
| ENSG00000231826 | LINC01819 | -2.063 | 0.635 | -3.248 | 1.16E-03 | 0.012 | -2.689 | 1.200 | -2.240 | 0.025 | 0.198 |
| ENSG00000167840 | ZNF232 | 0.451 | 0.141 | 3.206 | 1.35E-03 | 0.014 | 0.626 | 0.280 | 2.240 | 0.025 | 0.198 |
| ENSG00000122644 | ARL4A | -0.485 | 0.148 | -3.274 | 1.06E-03 | 0.012 | -0.753 | 0.337 | -2.236 | 0.025 | 0.199 |
| ENSG00000137968 | SLC44A5 | -1.620 | 0.499 | -3.249 | 1.16E-03 | 0.012 | -1.800 | 0.805 | -2.235 | 0.025 | 0.199 |
| ENSG00000137411 | VARS2 | 0.302 | 0.105 | 2.870 | 4.10E-03 | 0.030 | 0.502 | 0.225 | 2.234 | 0.025 | 0.199 |
| ENSG00000110911 | SLC11A2 | -0.473 | 0.161 | -2.936 | 3.32E-03 | 0.026 | -0.613 | 0.275 | -2.233 | 0.026 | 0.199 |
| ENSG00000254290 |  | -1.076 | 0.217 | -4.965 | 6.88E-07 | 5.84E-05 | -1.791 | 0.802 | -2.232 | 0.026 | 0.200 |
| ENSG00000176153 | GPX2 | -0.526 | 0.175 | -3.010 | 2.61E-03 | 0.022 | -1.737 | 0.779 | -2.230 | 0.026 | 0.200 |
| ENSG00000132517 | SLC52A1 | 1.142 | 0.334 | 3.421 | 6.23E-04 | 7.96E-03 | 1.882 | 0.847 | 2.223 | 0.026 | 0.203 |
| ENSG00000004864 | SLC25A13 | -0.427 | 0.110 | -3.898 | 9.72E-05 | 2.22E-03 | -0.533 | 0.240 | -2.222 | 0.026 | 0.203 |
| ENSG00000164855 | TMEM184A | -0.485 | 0.163 | -2.966 | 3.02E-03 | 0.024 | -1.095 | 0.495 | -2.214 | 0.027 | 0.205 |
| ENSG00000140511 | HAPLN3 | 0.487 | 0.178 | 2.737 | 6.20E-03 | 0.040 | 1.274 | 0.576 | 2.213 | 0.027 | 0.206 |
| ENSG00000177788 |  | -0.509 | 0.188 | -2.710 | 6.73E-03 | 0.042 | -0.762 | 0.345 | -2.210 | 0.027 | 0.206 |
| ENSG00000105865 | DUS4L | -0.627 | 0.121 | -5.194 | 2.06E-07 | 2.42E-05 | -0.513 | 0.233 | -2.207 | 0.027 | 0.207 |
| ENSG00000157368 | IL34 | -0.927 | 0.225 | -4.120 | 3.78E-05 | 1.14E-03 | -1.634 | 0.741 | -2.204 | 0.028 | 0.208 |
| ENSG00000215018 | COL28A1 | -1.084 | 0.337 | -3.215 | 1.30E-03 | 0.013 | -1.992 | 0.904 | -2.203 | 0.028 | 0.209 |
| ENSG00000163082 | SGPP2 | 0.576 | 0.164 | 3.510 | 4.48E-04 | 6.34E-03 | 1.069 | 0.487 | 2.195 | 0.028 | 0.211 |
| ENSG00000101346 | POFUT1 | -0.611 | 0.139 | -4.397 | 1.10E-05 | 4.71E-04 | -0.618 | 0.282 | -2.194 | 0.028 | 0.211 |
| ENSG00000109738 | GLRB | -0.918 | 0.253 | -3.633 | 2.80E-04 | 4.58E-03 | -1.885 | 0.860 | -2.191 | 0.028 | 0.212 |
| ENSG00000132964 | CDK8 | -0.441 | 0.112 | -3.922 | 8.80E-05 | 2.07E-03 | -0.517 | 0.236 | -2.191 | 0.028 | 0.212 |
| ENSG00000148655 | LRMDA | -0.802 | 0.268 | -2.996 | 2.73E-03 | 0.022 | -1.768 | 0.808 | -2.188 | 0.029 | 0.213 |
| ENSG00000186907 | RTN4RL2 | 1.257 | 0.245 | 5.133 | 2.85E-07 | 3.04E-05 | 1.259 | 0.577 | 2.181 | 0.029 | 0.215 |
| ENSG00000104537 | ANXA13 | -1.334 | 0.319 | -4.174 | 2.99E-05 | 9.56E-04 | -2.058 | 0.945 | -2.178 | 0.029 | 0.216 |
| ENSG00000187546 | AGMO | -2.029 | 0.388 | -5.224 | 1.75E-07 | 2.12E-05 | -1.935 | 0.890 | -2.174 | 0.030 | 0.217 |
| ENSG00000109472 | CPE | -1.910 | 0.305 | -6.258 | 3.90E-10 | 1.82E-07 | -1.769 | 0.814 | -2.174 | 0.030 | 0.217 |
| ENSG00000232850 |  | -0.822 | 0.263 | -3.120 | 1.81E-03 | 0.017 | -1.103 | 0.507 | -2.173 | 0.030 | 0.217 |
| ENSG00000182247 | UBE2E2 | -0.684 | 0.196 | -3.491 | 4.81E-04 | 6.62E-03 | -1.582 | 0.728 | -2.173 | 0.030 | 0.217 |
| ENSG00000123405 | NFE2 | -1.804 | 0.407 | -4.436 | 9.15E-06 | 4.12E-04 | -1.745 | 0.803 | -2.172 | 0.030 | 0.217 |
| ENSG00000119772 | DNMT3A | -0.331 | 0.116 | -2.858 | 4.26E-03 | 0.031 | -0.642 | 0.296 | -2.167 | 0.030 | 0.219 |
| ENSG00000170852 | KBTBD2 | -0.293 | 0.075 | -3.901 | 9.57E-05 | 2.20E-03 | -0.396 | 0.183 | -2.163 | 0.031 | 0.220 |
| ENSG00000078699 | CBFA2T2 | -0.414 | 0.115 | -3.595 | 3.25E-04 | 5.00E-03 | -0.489 | 0.226 | -2.162 | 0.031 | 0.221 |
| ENSG00000213337 | ANKRD39 | 0.413 | 0.114 | 3.628 | 2.86E-04 | 4.63E-03 | 0.376 | 0.174 | 2.158 | 0.031 | 0.222 |
| ENSG00000100359 | SGSM3 | 0.349 | 0.109 | 3.209 | 1.33E-03 | 0.014 | 0.400 | 0.185 | 2.157 | 0.031 | 0.222 |
| ENSG00000213420 | GPC2 | -0.873 | 0.215 | -4.050 | 5.11E-05 | 1.42E-03 | -0.939 | 0.436 | -2.150 | 0.032 | 0.224 |
| ENSG00000256443 |  | -0.748 | 0.283 | -2.643 | 8.22E-03 | 0.048 | -1.187 | 0.552 | -2.150 | 0.032 | 0.224 |
| ENSG00000261423 | TMEM202-AS1 | -0.431 | 0.153 | -2.823 | 4.75E-03 | 0.033 | -0.605 | 0.282 | -2.149 | 0.032 | 0.225 |
| ENSG00000226453 | LINC02542 | -1.590 | 0.360 | -4.418 | 9.97E-06 | 4.40E-04 | -1.671 | 0.779 | -2.144 | 0.032 | 0.226 |
| ENSG00000178184 | PARD6G | 0.541 | 0.160 | 3.387 | 7.05E-04 | 8.69E-03 | 1.030 | 0.481 | 2.142 | 0.032 | 0.227 |
| ENSG00000101546 | RBFA | 0.419 | 0.116 | 3.609 | 3.07E-04 | 4.83E-03 | 0.485 | 0.227 | 2.141 | 0.032 | 0.227 |
| ENSG00000163755 | HPS3 | -0.337 | 0.094 | -3.588 | 3.33E-04 | 5.09E-03 | -0.363 | 0.170 | -2.138 | 0.033 | 0.228 |
| ENSG00000091164 | TXNL1 | 0.323 | 0.105 | 3.080 | 2.07E-03 | 0.018 | 0.439 | 0.205 | 2.137 | 0.033 | 0.228 |
| ENSG00000144306 | SCRN3 | -0.386 | 0.124 | -3.105 | 1.90E-03 | 0.017 | -0.483 | 0.226 | -2.136 | 0.033 | 0.228 |
| ENSG00000228223 | HCG11 | -1.104 | 0.249 | -4.442 | 8.93E-06 | 4.07E-04 | -1.492 | 0.700 | -2.133 | 0.033 | 0.229 |
| ENSG00000144233 | AMMECR1L | -0.195 | 0.069 | -2.806 | 5.01E-03 | 0.034 | -0.363 | 0.170 | -2.133 | 0.033 | 0.229 |
| ENSG00000119711 | ALDH6A1 | 0.648 | 0.149 | 4.347 | 1.38E-05 | 5.46E-04 | 0.751 | 0.352 | 2.133 | 0.033 | 0.229 |
| ENSG00000101158 | NELFCD | -0.430 | 0.117 | -3.687 | 2.27E-04 | 3.98E-03 | -0.738 | 0.346 | -2.132 | 0.033 | 0.229 |
| ENSG00000105707 | HPN | -1.685 | 0.521 | -3.233 | 1.23E-03 | 0.013 | -1.818 | 0.855 | -2.126 | 0.034 | 0.231 |
| ENSG00000225526 | MKRN2OS | 0.843 | 0.174 | 4.841 | 1.29E-06 | 9.51E-05 | 0.858 | 0.404 | 2.123 | 0.034 | 0.232 |
| ENSG00000186376 | ZNF75D | -0.402 | 0.123 | -3.277 | 1.05E-03 | 0.011 | -0.510 | 0.240 | -2.121 | 0.034 | 0.232 |
| ENSG00000146955 | RAB19 | 0.639 | 0.172 | 3.719 | 2.00E-04 | 3.65E-03 | 1.123 | 0.529 | 2.121 | 0.034 | 0.232 |
| ENSG00000225177 |  | -0.572 | 0.167 | -3.432 | 5.99E-04 | 7.75E-03 | -1.069 | 0.505 | -2.119 | 0.034 | 0.233 |
| ENSG00000130751 | NPAS1 | 0.621 | 0.225 | 2.767 | 5.66E-03 | 0.037 | 0.956 | 0.452 | 2.116 | 0.034 | 0.234 |
| ENSG00000253320 | MAILR | -0.764 | 0.208 | -3.669 | 2.43E-04 | 4.14E-03 | -0.838 | 0.396 | -2.115 | 0.034 | 0.234 |
| ENSG00000242575 | TUBAP13 | -3.237 | 0.676 | -4.785 | 1.71E-06 | 1.14E-04 | -2.380 | 1.128 | -2.111 | 0.035 | 0.236 |
| ENSG00000263050 |  | 0.573 | 0.164 | 3.486 | 4.91E-04 | 6.70E-03 | 0.794 | 0.377 | 2.105 | 0.035 | 0.238 |
| ENSG00000253161 | LINC01605 | -0.835 | 0.255 | -3.276 | 1.05E-03 | 0.011 | -1.737 | 0.826 | -2.103 | 0.035 | 0.238 |
| ENSG00000176890 | TYMS | 0.467 | 0.136 | 3.443 | 5.76E-04 | 7.56E-03 | 0.628 | 0.299 | 2.103 | 0.036 | 0.239 |
| ENSG00000170291 | ELP5 | 0.453 | 0.115 | 3.932 | 8.43E-05 | 2.00E-03 | 0.464 | 0.221 | 2.101 | 0.036 | 0.239 |
| ENSG00000101464 | PIGU | -0.366 | 0.118 | -3.092 | 1.99E-03 | 0.018 | -0.496 | 0.236 | -2.101 | 0.036 | 0.239 |
| ENSG00000266208 |  | 0.513 | 0.179 | 2.864 | 4.18E-03 | 0.030 | 0.814 | 0.387 | 2.100 | 0.036 | 0.239 |
| ENSG00000254111 |  | -1.746 | 0.360 | -4.846 | 1.26E-06 | 9.42E-05 | -1.734 | 0.826 | -2.098 | 0.036 | 0.240 |
| ENSG00000185722 | ANKFY1 | 0.363 | 0.105 | 3.461 | 5.38E-04 | 7.20E-03 | 0.330 | 0.157 | 2.098 | 0.036 | 0.240 |
| ENSG00000182405 | PGBD4 | -0.700 | 0.162 | -4.322 | 1.55E-05 | 5.94E-04 | -0.674 | 0.322 | -2.094 | 0.036 | 0.241 |
| ENSG00000172828 | CES3 | 0.709 | 0.266 | 2.663 | 7.75E-03 | 0.047 | 1.370 | 0.655 | 2.091 | 0.037 | 0.243 |
| ENSG00000178252 | WDR6 | 0.361 | 0.118 | 3.054 | 2.26E-03 | 0.020 | 0.298 | 0.142 | 2.089 | 0.037 | 0.243 |
| ENSG00000100373 | UPK3A | -1.265 | 0.471 | -2.685 | 7.26E-03 | 0.045 | -2.548 | 1.220 | -2.088 | 0.037 | 0.243 |
| ENSG00000126243 | LRFN3 | 0.728 | 0.147 | 4.958 | 7.12E-07 | 5.99E-05 | 0.677 | 0.325 | 2.086 | 0.037 | 0.244 |
| ENSG00000126261 | UBA2 | -0.700 | 0.104 | -6.714 | 1.90E-11 | 1.42E-08 | -0.419 | 0.201 | -2.086 | 0.037 | 0.244 |
| ENSG00000132950 | ZMYM5 | -0.380 | 0.128 | -2.961 | 3.07E-03 | 0.024 | -0.570 | 0.273 | -2.084 | 0.037 | 0.244 |
| ENSG00000225889 |  | -0.984 | 0.253 | -3.894 | 9.87E-05 | 2.25E-03 | -1.327 | 0.637 | -2.083 | 0.037 | 0.245 |
| ENSG00000146039 | SLC17A4 | -1.240 | 0.342 | -3.624 | 2.90E-04 | 4.67E-03 | -2.312 | 1.113 | -2.077 | 0.038 | 0.247 |
| ENSG00000132792 | CTNNBL1 | -0.443 | 0.111 | -3.991 | 6.59E-05 | 1.71E-03 | -0.445 | 0.214 | -2.075 | 0.038 | 0.248 |
| ENSG00000231764 | DLX6-AS1 | -2.607 | 0.654 | -3.987 | 6.71E-05 | 1.73E-03 | -1.827 | 0.882 | -2.072 | 0.038 | 0.249 |
| ENSG00000131019 | ULBP3 | 0.823 | 0.184 | 4.472 | 7.77E-06 | 3.72E-04 | 0.819 | 0.395 | 2.072 | 0.038 | 0.249 |
| ENSG00000198626 | RYR2 | -1.463 | 0.339 | -4.316 | 1.59E-05 | 6.03E-04 | -1.940 | 0.937 | -2.070 | 0.038 | 0.249 |
| ENSG00000158941 | CCAR2 | 0.362 | 0.118 | 3.066 | 2.17E-03 | 0.019 | 0.440 | 0.213 | 2.070 | 0.038 | 0.249 |
| ENSG00000074356 | NCBP3 | 0.378 | 0.099 | 3.810 | 1.39E-04 | 2.89E-03 | 0.335 | 0.162 | 2.068 | 0.039 | 0.250 |
| ENSG00000189056 | RELN | -0.921 | 0.345 | -2.671 | 7.57E-03 | 0.046 | -1.647 | 0.798 | -2.065 | 0.039 | 0.251 |
| ENSG00000114646 | CSPG5 | 1.229 | 0.292 | 4.203 | 2.64E-05 | 8.67E-04 | 0.977 | 0.474 | 2.064 | 0.039 | 0.252 |
| ENSG00000177238 | TRIM72 | 2.310 | 0.625 | 3.696 | 2.19E-04 | 3.88E-03 | 1.815 | 0.880 | 2.063 | 0.039 | 0.252 |
| ENSG00000267060 | PTGES3L | -0.670 | 0.220 | -3.041 | 2.35E-03 | 0.020 | -0.991 | 0.481 | -2.061 | 0.039 | 0.252 |
| ENSG00000122912 | SLC25A16 | -0.360 | 0.101 | -3.555 | 3.78E-04 | 5.58E-03 | -0.441 | 0.214 | -2.059 | 0.039 | 0.253 |
| ENSG00000179674 | ARL14 | -0.577 | 0.203 | -2.839 | 4.53E-03 | 0.032 | -1.404 | 0.682 | -2.058 | 0.040 | 0.253 |
| ENSG00000101417 | PXMP4 | -0.356 | 0.124 | -2.871 | 4.10E-03 | 0.030 | -0.896 | 0.436 | -2.058 | 0.040 | 0.253 |
| ENSG00000236423 | LINC01134 | -0.691 | 0.242 | -2.855 | 4.30E-03 | 0.031 | -1.095 | 0.532 | -2.056 | 0.040 | 0.254 |
| ENSG00000135709 | KIAA0513 | 0.575 | 0.180 | 3.190 | 1.43E-03 | 0.014 | 0.946 | 0.461 | 2.053 | 0.040 | 0.255 |
| ENSG00000179715 | PCED1B | -0.789 | 0.187 | -4.229 | 2.35E-05 | 8.03E-04 | -1.503 | 0.733 | -2.052 | 0.040 | 0.255 |
| ENSG00000186994 | KANK3 | 0.669 | 0.156 | 4.293 | 1.76E-05 | 6.46E-04 | 1.318 | 0.645 | 2.046 | 0.041 | 0.257 |
| ENSG00000157978 | LDLRAP1 | 0.412 | 0.110 | 3.740 | 1.84E-04 | 3.45E-03 | 0.460 | 0.225 | 2.043 | 0.041 | 0.258 |
| ENSG00000132334 | PTPRE | 0.481 | 0.105 | 4.562 | 5.06E-06 | 2.69E-04 | 0.980 | 0.480 | 2.040 | 0.041 | 0.259 |
| ENSG00000196605 | ZNF846 | -0.577 | 0.137 | -4.219 | 2.46E-05 | 8.23E-04 | -0.794 | 0.390 | -2.035 | 0.042 | 0.261 |
| ENSG00000132763 | MMACHC | 0.316 | 0.100 | 3.163 | 1.56E-03 | 0.015 | 0.559 | 0.276 | 2.024 | 0.043 | 0.264 |
| ENSG00000174365 | SNHG11 | -0.510 | 0.134 | -3.820 | 1.33E-04 | 2.80E-03 | -0.527 | 0.261 | -2.019 | 0.043 | 0.266 |
| ENSG00000272711 |  | -0.726 | 0.187 | -3.881 | 1.04E-04 | 2.33E-03 | -0.847 | 0.420 | -2.019 | 0.043 | 0.266 |
| ENSG00000181826 | RELL1 | 0.598 | 0.161 | 3.713 | 2.05E-04 | 3.69E-03 | 0.638 | 0.316 | 2.019 | 0.044 | 0.266 |
| ENSG00000232874 |  | -1.115 | 0.259 | -4.307 | 1.66E-05 | 6.18E-04 | -0.898 | 0.445 | -2.018 | 0.044 | 0.267 |
| ENSG00000167721 | TSR1 | 0.445 | 0.100 | 4.471 | 7.79E-06 | 3.72E-04 | 0.384 | 0.191 | 2.014 | 0.044 | 0.267 |
| ENSG00000141499 | WRAP53 | 0.554 | 0.110 | 5.051 | 4.38E-07 | 4.15E-05 | 0.431 | 0.214 | 2.014 | 0.044 | 0.267 |
| ENSG00000213937 | CLDN9 | -1.057 | 0.346 | -3.056 | 2.24E-03 | 0.019 | -1.441 | 0.716 | -2.014 | 0.044 | 0.267 |
| ENSG00000182621 | PLCB1 | -1.284 | 0.245 | -5.233 | 1.67E-07 | 2.04E-05 | -1.397 | 0.695 | -2.010 | 0.044 | 0.269 |
| ENSG00000123892 | RAB38 | 0.760 | 0.287 | 2.648 | 8.11E-03 | 0.048 | 1.704 | 0.848 | 2.009 | 0.045 | 0.269 |
| ENSG00000132182 | NUP210 | 0.568 | 0.207 | 2.745 | 6.05E-03 | 0.039 | 0.895 | 0.446 | 2.008 | 0.045 | 0.269 |
| ENSG00000119537 | KDSR | 0.587 | 0.102 | 5.764 | 8.19E-09 | 2.01E-06 | 0.554 | 0.276 | 2.008 | 0.045 | 0.269 |
| ENSG00000120690 | ELF1 | -0.432 | 0.132 | -3.277 | 1.05E-03 | 0.011 | -0.503 | 0.252 | -2.001 | 0.045 | 0.272 |
| ENSG00000102796 | DHRS12 | -0.461 | 0.138 | -3.348 | 8.14E-04 | 0.010 | -0.731 | 0.366 | -2.001 | 0.045 | 0.272 |
| ENSG00000091428 | RAPGEF4 | -0.709 | 0.222 | -3.190 | 1.42E-03 | 0.014 | -1.712 | 0.856 | -2.001 | 0.045 | 0.272 |
| ENSG00000187624 | C17orf97 | 0.734 | 0.234 | 3.138 | 1.70E-03 | 0.016 | 0.938 | 0.469 | 1.999 | 0.046 | 0.273 |
| ENSG00000174292 | TNK1 | 0.745 | 0.112 | 6.649 | 2.95E-11 | 2.12E-08 | 0.695 | 0.348 | 1.998 | 0.046 | 0.273 |
| ENSG00000138336 | TET1 | -0.627 | 0.228 | -2.752 | 5.92E-03 | 0.038 | -1.389 | 0.696 | -1.997 | 0.046 | 0.273 |
| ENSG00000227502 | MROCKI | -0.965 | 0.235 | -4.097 | 4.18E-05 | 1.23E-03 | -1.581 | 0.792 | -1.995 | 0.046 | 0.274 |
| ENSG00000101544 | ADNP2 | 0.443 | 0.092 | 4.842 | 1.29E-06 | 9.51E-05 | 0.460 | 0.231 | 1.994 | 0.046 | 0.274 |
| ENSG00000213462 | ERV3-1 | -1.078 | 0.243 | -4.435 | 9.19E-06 | 4.13E-04 | -1.019 | 0.511 | -1.994 | 0.046 | 0.274 |
| ENSG00000100429 | HDAC10 | 0.404 | 0.153 | 2.650 | 8.06E-03 | 0.048 | 0.579 | 0.291 | 1.990 | 0.047 | 0.275 |
| ENSG00000270012 |  | -0.918 | 0.199 | -4.613 | 3.96E-06 | 2.23E-04 | -0.766 | 0.385 | -1.989 | 0.047 | 0.275 |
| ENSG00000103550 | KNOP1 | -0.296 | 0.096 | -3.086 | 2.03E-03 | 0.018 | -0.389 | 0.196 | -1.985 | 0.047 | 0.277 |
| ENSG00000010704 | HFE | 0.410 | 0.125 | 3.273 | 1.06E-03 | 0.012 | 0.696 | 0.351 | 1.980 | 0.048 | 0.278 |
| ENSG00000022267 | FHL1 | -0.727 | 0.181 | -4.005 | 6.21E-05 | 1.63E-03 | -1.339 | 0.676 | -1.980 | 0.048 | 0.278 |
| ENSG00000227110 | LMCD1-AS1 | -0.790 | 0.234 | -3.369 | 7.54E-04 | 9.12E-03 | -1.181 | 0.597 | -1.978 | 0.048 | 0.279 |
| ENSG00000158691 | ZSCAN12 | -0.732 | 0.164 | -4.470 | 7.83E-06 | 3.72E-04 | -0.674 | 0.341 | -1.976 | 0.048 | 0.280 |
| ENSG00000065883 | CDK13 | -0.247 | 0.077 | -3.228 | 1.25E-03 | 0.013 | -0.371 | 0.188 | -1.971 | 0.049 | 0.282 |
| ENSG00000164830 | OXR1 | -0.583 | 0.157 | -3.719 | 2.00E-04 | 3.65E-03 | -0.750 | 0.381 | -1.971 | 0.049 | 0.282 |
| ENSG00000173597 | SULT1B1 | -0.965 | 0.273 | -3.537 | 4.05E-04 | 5.88E-03 | -2.112 | 1.073 | -1.969 | 0.049 | 0.283 |
| ENSG00000267493 | CIRBP-AS1 | 0.612 | 0.178 | 3.441 | 5.80E-04 | 7.60E-03 | 0.370 | 0.188 | 1.968 | 0.049 | 0.283 |
| ENSG00000164961 | WASHC5 | -0.431 | 0.115 | -3.737 | 1.86E-04 | 3.46E-03 | -0.956 | 0.487 | -1.963 | 0.050 | 0.284 |
| ENSG00000171105 | INSR | 0.400 | 0.119 | 3.365 | 7.66E-04 | 9.22E-03 | 0.581 | 0.296 | 1.963 | 0.050 | 0.284 |
