# Supplementary material for: Controlling for cellular heterogeneity using single-cell deconvolution of gene expression reveals novel markers of colorectal tumors exhibiting microsatellite instability
Source: Oncotarget. 2021 Apr 13;12(8):767–82. doi: 10.18632/oncotarget.27935 (PMC8057268; doi:10.18632/oncotarget.27935)
Supplement: Supplementary file 4 [file oncotarget-12-767-s004.docx]

**Supplementary Table 4: Overview of modules identified in WGCNA**. NA values for PPI were generated in some small modules or in modules consisting of few protein coding genes. Negative correlations between the module eigengene and MSI status indicate modules consisting of genes that display reduced expression in MSI-H versus MSS/MSI-L tumors.

| Module | Module Size | No. Novel Genes | Novel Gene (%) | Cor | *P* | Bonferroni | PPI |
| --- | --- | --- | --- | --- | --- | --- | --- |
| blueviolet | 28 | 1 | 3.571 | -0.773 | 9.45E-59 | 9.07E-57 | 0.019 |
| antiquewhite2 | 207 | 13 | 6.280 | 0.563 | 1.35E-25 | 1.30E-23 | 6.66E-16 |
| darkseagreen3 | 87 | 11 | 12.644 | 0.552 | 1.72E-24 | 1.65E-22 | 4.05E-09 |
| lightblue4 | 106 | 4 | 3.774 | -0.471 | 2.36E-17 | 2.26E-15 | 3.51E-03 |
| cornflowerblue | 18 | 1 | 5.556 | 0.419 | 1.07E-13 | 1.03E-11 | 2.93E-06 |
| darkseagreen2 | 259 | 0 | 0.000 | -0.405 | 7.79E-13 | 7.48E-11 | 4.10E-11 |
| lightblue1 | 194 | 22 | 11.340 | -0.404 | 9.44E-13 | 9.06E-11 | < 1.0e-16 |
| lavenderblush | 19 | 2 | 10.526 | 0.402 | 1.20E-12 | 1.15E-10 | 1.77E-04 |
| bisque4 | 66 | 8 | 12.121 | 0.367 | 1.29E-10 | 1.23E-08 | 4.83E-04 |
| tan4 | 27 | 3 | 11.111 | -0.359 | 3.26E-10 | 3.13E-08 | 1.97E-11 |
| deeppink2 | 30 | 2 | 6.667 | 0.343 | 2.02E-09 | 1.94E-07 | 1.30E-05 |
| indianred3 | 127 | 13 | 10.236 | 0.343 | 2.23E-09 | 2.14E-07 | < 1.0e-16 |
| green3 | 19 | 5 | 26.316 | 0.333 | 6.22E-09 | 5.97E-07 | < 1.0e-16 |
| aliceblue | 95 | 6 | 6.316 | 0.312 | 5.81E-08 | 5.58E-06 | 4.11E-14 |
| purple2 | 16 | 2 | 12.500 | 0.305 | 1.22E-07 | 1.18E-05 | 2.94E-08 |
| paleturquoise4 | 53 | 7 | 13.208 | 0.304 | 1.39E-07 | 1.33E-05 | 1.03E-03 |
| wheat3 | 13 | 1 | 7.692 | -0.295 | 3.14E-07 | 3.01E-05 | < 1.0e-16 |
| lightblue3 | 75 | 9 | 12.000 | 0.284 | 8.91E-07 | 8.56E-05 | 9.35E-07 |
| brown4 | 1213 | 81 | 6.678 | 0.283 | 1.01E-06 | 9.69E-05 | < 1.0e-16 |
| darkolivegreen2 | 48 | 1 | 2.083 | 0.261 | 7.01E-06 | 6.73E-04 | 4.66E-10 |
| blue2 | 39 | 2 | 5.128 | 0.259 | 7.98E-06 | 7.66E-04 | 1.37E-04 |
| coral | 61 | 2 | 3.279 | -0.259 | 8.31E-06 | 7.98E-04 | 9.64E-04 |
| paleturquoise | 97 | 3 | 3.093 | 0.258 | 8.58E-06 | 8.24E-04 | < 1.0e-16 |
| plum | 46 | 1 | 2.174 | 0.255 | 1.14E-05 | 1.10E-03 | < 1.0e-16 |
| tan3 | 20 | 1 | 5.000 | 0.248 | 1.95E-05 | 1.87E-03 | < 1.0e-16 |
| darkturquoise | 120 | 4 | 3.333 | -0.246 | 2.28E-05 | 2.19E-03 | < 1.0e-16 |
| tomato | 127 | 2 | 1.575 | -0.244 | 2.81E-05 | 2.69E-03 | < 1.0e-16 |
| yellow3 | 208 | 8 | 3.846 | 0.241 | 3.50E-05 | 3.36E-03 | < 1.0e-16 |
| darkseagreen | 14 | 0 | 0.000 | 0.239 | 3.93E-05 | 3.77E-03 | 1.31E-11 |
| darkslateblue | 64 | 0 | 0.000 | -0.235 | 5.66E-05 | 5.43E-03 | < 1.0e-16 |
| dodgerblue4 | 36 | 0 | 0.000 | -0.231 | 7.59E-05 | 7.29E-03 | 9.46E-05 |
| whitesmoke | 17 | 0 | 0.000 | -0.214 | 2.41E-04 | 0.023 | 2.62E-05 |
| coral2 | 6989 | 197 | 2.819 | -0.212 | 2.92E-04 | 0.028 | < 1.0e-16 |
| chocolate2 | 13 | 0 | 0.000 | -0.207 | 4.08E-04 | 0.039 | < 1.0e-16 |
| blue3 | 20 | 0 | 0.000 | -0.205 | 4.57E-04 | 0.044 | NA |
| diumpurple4 | 31 | 0 | 0.000 | -0.195 | 8.69E-04 | 0.083 | 1.57E-03 |
| grey60 | 150 | 16 | 10.667 | -0.189 | 1.27E-03 | 0.122 | < 1.0e-16 |
| pink3 | 22 | 1 | 4.545 | -0.184 | 1.64E-03 | 0.157 | 9.63E-03 |
| lightskyblue4 | 22 | 1 | 4.545 | 0.184 | 1.66E-03 | 0.159 | 4.40E-03 |
| antiquewhite1 | 130 | 0 | 0.000 | -0.176 | 2.62E-03 | 0.251 | 1 |
| salmon1 | 27 | 0 | 0.000 | -0.175 | 2.79E-03 | 0.268 | < 1.0e-16 |
| darkolivegreen4 | 40 | 0 | 0.000 | 0.165 | 4.96E-03 | 0.476 | 0.048 |
| orange4 | 16 | 0 | 0.000 | 0.164 | 5.15E-03 | 0.495 | < 1.0e-16 |
| royalblue2 | 15 | 3 | 20.000 | 0.155 | 8.48E-03 | 0.814 | 4.38E-07 |
| floralwhite | 69 | 2 | 2.899 | -0.045 | 0.443 | 1 | 1.11E-16 |
| navajowhite3 | 17 | 0 | 0.000 | 0.016 | 0.781 | 1 | 3.34E-14 |
| thistle4 | 27 | 0 | 0.000 | -0.087 | 0.141 | 1 | 5.56E-11 |
| orangered1 | 279 | 0 | 0.000 | -0.094 | 0.112 | 1 | 1.12E-10 |
| lightskyblue2 | 186 | 5 | 2.688 | 0.145 | 0.014 | 1 | 2.79E-10 |
| pink2 | 48 | 0 | 0.000 | 0.003 | 0.958 | 1 | 3.70E-09 |
| midnightblue | 160 | 1 | 0.625 | -0.126 | 0.032 | 1 | 7.70E-09 |
| coral1 | 48 | 0 | 0.000 | 0.119 | 0.043 | 1 | 2.58E-07 |
| firebrick3 | 28 | 0 | 0.000 | -0.024 | 0.687 | 1 | 6.63E-07 |
| lightpink | 198 | 0 | 0.000 | 0.128 | 0.030 | 1 | 2.01E-05 |
| ivory | 72 | 0 | 0.000 | 0.077 | 0.190 | 1 | 2.51E-05 |
| darkgoldenrod3 | 10 | 0 | 0.000 | -0.129 | 0.028 | 1 | 8.96E-05 |
| darkgreen | 120 | 2 | 1.667 | 0.073 | 0.215 | 1 | 1.36E-04 |
| antiquewhite | 13 | 0 | 0.000 | 0.052 | 0.374 | 1 | 2.07E-04 |
| tan2 | 15 | 0 | 0.000 | 0.113 | 0.054 | 1 | 3.32E-04 |
| plum2 | 64 | 0 | 0.000 | -0.147 | 0.013 | 1 | 3.99E-04 |
| indianred1 | 16 | 0 | 0.000 | -0.103 | 0.079 | 1 | 7.16E-04 |
| lavenderblush1 | 41 | 2 | 4.878 | 0.139 | 0.018 | 1 | 1.16E-03 |
| lightblue2 | 16 | 0 | 0.000 | 0.087 | 0.141 | 1 | 1.68E-03 |
| skyblue2 | 47 | 3 | 6.383 | 0.133 | 0.024 | 1 | 2.49E-03 |
| sienna | 12 | 1 | 8.333 | -0.026 | 0.658 | 1 | 0.016 |
| magenta1 | 38 | 0 | 0.000 | 0.037 | 0.526 | 1 | 0.031 |
| lavender | 14 | 0 | 0.000 | 0.067 | 0.259 | 1 | 0.032 |
| magenta2 | 20 | 0 | 0.000 | -0.098 | 0.097 | 1 | 0.082 |
| lightpink1 | 20 | 0 | 0.000 | -0.073 | 0.214 | 1 | 0.203 |
| orangered | 22 | 4 | 18.182 | -0.062 | 0.296 | 1 | 0.263 |
| navajowhite4 | 13 | 0 | 0.000 | 0.027 | 0.647 | 1 | 1 |
| antiquewhite4 | 47 | 1 | 2.128 | 0.133 | 0.024 | 1 | < 1.0e-16 |
| thistle1 | 60 | 1 | 1.667 | 0.126 | 0.032 | 1 | < 1.0e-16 |
| orange3 | 11 | 1 | 9.091 | -0.112 | 0.058 | 1 | < 1.0e-16 |
| diumpurple1 | 29 | 1 | 3.448 | 0.079 | 0.179 | 1 | < 1.0e-16 |
| sienna3 | 88 | 1 | 1.136 | -0.009 | 0.880 | 1 | < 1.0e-16 |
| brown2 | 39 | 2 | 5.128 | -0.091 | 0.124 | 1 | < 1.0e-16 |
| darkorange2 | 525 | 2 | 0.381 | 0.037 | 0.527 | 1 | < 1.0e-16 |
| darkmagenta | 296 | 2 | 0.676 | 0.036 | 0.546 | 1 | < 1.0e-16 |
| indianred | 100 | 6 | 6.000 | -0.097 | 0.101 | 1 | < 1.0e-16 |
| darkolivegreen1 | 311 | 18 | 5.788 | 0.150 | 0.011 | 1 | < 1.0e-16 |
| blanchedalmond | 587 | 18 | 3.066 | -0.127 | 0.031 | 1 | < 1.0e-16 |
| chocolate4 | 1950 | 32 | 1.641 | -0.065 | 0.271 | 1 | < 1.0e-16 |
| blue1 | 43 | 0 | 0.000 | -0.113 | 0.054 | 1 | < 1.0e-16 |
| brown1 | 20 | 0 | 0.000 | -0.111 | 0.059 | 1 | < 1.0e-16 |
| navajowhite | 26 | 0 | 0.000 | 0.105 | 0.075 | 1 | < 1.0e-16 |
| diumorchid | 47 | 0 | 0.000 | 0.090 | 0.127 | 1 | < 1.0e-16 |
| plum1 | 78 | 0 | 0.000 | -0.080 | 0.176 | 1 | < 1.0e-16 |
| lavenderblush2 | 34 | 0 | 0.000 | 0.033 | 0.580 | 1 | < 1.0e-16 |
| magenta4 | 36 | 0 | 0.000 | 0.027 | 0.643 | 1 | < 1.0e-16 |
| yellow2 | 22 | 0 | 0.000 | -0.021 | 0.717 | 1 | < 1.0e-16 |
| diumorchid4 | 16 | 0 | 0.000 | 0.013 | 0.823 | 1 | < 1.0e-16 |
| indianred4 | 40 | 0 | 0.000 | 0.011 | 0.850 | 1 | < 1.0e-16 |
| red | 271 | 5 | 1.845 | 0.088 | 0.136 | 1 | NA |
| grey | 2 | 0 | 0.000 | -0.072 | 0.221 | 1 | NA |
| yellow4 | 46 | 0 | 0.000 | -0.048 | 0.420 | 1 | NA |
