# Supplementary material for: Controlling for cellular heterogeneity using single-cell deconvolution of gene expression reveals novel markers of colorectal tumors exhibiting microsatellite instability
Source: Oncotarget. 2021 Apr 13;12(8):767–82. doi: 10.18632/oncotarget.27935 (PMC8057268; doi:10.18632/oncotarget.27935)
Supplement: Supplementary file 5 [file oncotarget-12-767-s005.docx]

**Supplementary Table 5: Novel significant DEGs (*q* = 0.05) identified in TCGA-COAD regression of MSI-H vs MSS/MSI-L tumors following adjustment for cell composition that were replicated in a similar analysis of colon cancer cell lines
(*P* = 0.05).** Negative log_2_Fold change corresponds to DEGs displaying significantly reduced expression in MSI-H tumors and cell lines.

| Ensembl | HGNC | TCGA-COAD | | | | | CCLE | | | | |
| --- | --- | --- | --- | --- | --- | --- | --- | --- | --- | --- | --- |
|  |  | **log2Fold Change** | **lfcSE** | **Stat** | ***P*** | **FDR** | **log2Fold Change** | **lfcSE** | **Stat** | ***P*** | **FDR** |
| ENSG00000187546 | AGMO | -2.029 | 0.388 | -5.224 | 1.75E-07 | 2.12E-05 | -1.935 | 0.890 | -2.174 | 0.030 | 0.217 |
| ENSG00000228742 | LINC02577 | -1.638 | 0.336 | -4.879 | 1.06E-06 | 8.21E-05 | -3.420 | 0.869 | -3.937 | 8.25E-05 | 5.76E-03 |
| ENSG00000130294 | KIF1A | -2.053 | 0.448 | -4.580 | 4.64E-06 | 2.54E-04 | -2.057 | 0.613 | -3.356 | 7.92E-04 | 0.026 |
| ENSG00000198626 | RYR2 | -1.463 | 0.339 | -4.316 | 1.59E-05 | 6.03E-04 | -1.940 | 0.937 | -2.070 | 0.038 | 0.249 |
| ENSG00000185905 | C16orf54 | -1.044 | 0.249 | -4.191 | 2.78E-05 | 9.04E-04 | -4.330 | 0.981 | -4.416 | 1.00E-05 | 1.28E-03 |
| ENSG00000104537 | ANXA13 | -1.334 | 0.319 | -4.174 | 2.99E-05 | 9.56E-04 | -2.058 | 0.945 | -2.178 | 0.029 | 0.216 |
| ENSG00000225889 |  | -0.984 | 0.253 | -3.894 | 9.87E-05 | 2.25E-03 | -1.327 | 0.637 | -2.083 | 0.037 | 0.245 |
| ENSG00000168913 | ENHO | 1.267 | 0.336 | 3.775 | 1.60E-04 | 3.17E-03 | 1.574 | 0.684 | 2.301 | 0.021 | 0.181 |
| ENSG00000157978 | LDLRAP1 | 0.412 | 0.110 | 3.740 | 1.84E-04 | 3.45E-03 | 0.460 | 0.225 | 2.043 | 0.041 | 0.258 |
| ENSG00000146955 | RAB19 | 0.639 | 0.172 | 3.719 | 2.00E-04 | 3.65E-03 | 1.123 | 0.529 | 2.121 | 0.034 | 0.232 |
| ENSG00000105472 | CLEC11A | 0.597 | 0.160 | 3.718 | 2.01E-04 | 3.66E-03 | 2.275 | 0.706 | 3.220 | 1.28E-03 | 0.035 |
| ENSG00000106178 | CCL24 | -1.157 | 0.312 | -3.713 | 2.05E-04 | 3.69E-03 | -2.518 | 1.064 | -2.366 | 0.018 | 0.165 |
| ENSG00000068489 | PRR11 | -0.445 | 0.121 | -3.680 | 2.33E-04 | 4.04E-03 | -0.463 | 0.201 | -2.301 | 0.021 | 0.181 |
| ENSG00000164761 | TNFRSF11B | -1.080 | 0.296 | -3.646 | 2.67E-04 | 4.39E-03 | -2.192 | 0.866 | -2.530 | 0.011 | 0.128 |
| ENSG00000163755 | HPS3 | -0.337 | 0.094 | -3.588 | 3.33E-04 | 5.09E-03 | -0.363 | 0.170 | -2.138 | 0.033 | 0.228 |
| ENSG00000182247 | UBE2E2 | -0.684 | 0.196 | -3.491 | 4.81E-04 | 6.62E-03 | -1.582 | 0.728 | -2.173 | 0.030 | 0.217 |
| ENSG00000222033 | LINC01124 | -1.054 | 0.306 | -3.446 | 5.69E-04 | 7.52E-03 | -2.371 | 0.766 | -3.096 | 1.96E-03 | 0.045 |
| ENSG00000206535 | LNP1 | -0.609 | 0.178 | -3.422 | 6.21E-04 | 7.94E-03 | -1.103 | 0.435 | -2.533 | 0.011 | 0.128 |
| ENSG00000159182 | PRAC1 | -2.698 | 0.793 | -3.403 | 6.67E-04 | 8.37E-03 | -6.728 | 2.003 | -3.359 | 7.81E-04 | 0.025 |
| ENSG00000123560 | PLP1 | -1.470 | 0.437 | -3.368 | 7.56E-04 | 9.14E-03 | -3.037 | 0.788 | -3.856 | 1.15E-04 | 7.28E-03 |
| ENSG00000130035 | GALNT8 | -1.055 | 0.314 | -3.360 | 7.81E-04 | 9.34E-03 | -1.788 | 0.735 | -2.432 | 0.015 | 0.149 |
| ENSG00000039600 | SOX30 | -1.007 | 0.304 | -3.311 | 9.30E-04 | 0.011 | -2.303 | 0.852 | -2.704 | 6.84E-03 | 0.096 |
| ENSG00000231826 | LINC01819 | -2.063 | 0.635 | -3.248 | 1.16E-03 | 0.012 | -2.689 | 1.200 | -2.240 | 0.025 | 0.198 |
| ENSG00000165617 | DACT1 | -0.658 | 0.205 | -3.205 | 1.35E-03 | 0.014 | -1.950 | 0.840 | -2.321 | 0.020 | 0.176 |
| ENSG00000135709 | KIAA0513 | 0.575 | 0.180 | 3.190 | 1.43E-03 | 0.014 | 0.946 | 0.461 | 2.053 | 0.040 | 0.255 |
| ENSG00000116194 | ANGPTL1 | -1.032 | 0.324 | -3.181 | 1.47E-03 | 0.015 | -1.718 | 0.758 | -2.266 | 0.023 | 0.190 |
| ENSG00000187624 | C17orf97 | 0.734 | 0.234 | 3.138 | 1.70E-03 | 0.016 | 0.938 | 0.469 | 1.999 | 0.046 | 0.273 |
| ENSG00000266402 | SNHG25 | 1.235 | 0.399 | 3.094 | 1.98E-03 | 0.018 | 0.880 | 0.328 | 2.685 | 7.26E-03 | 0.099 |
| ENSG00000118514 | ALDH8A1 | 1.053 | 0.341 | 3.089 | 2.01E-03 | 0.018 | 1.905 | 0.614 | 3.102 | 1.92E-03 | 0.044 |
| ENSG00000185652 | NTF3 | 0.800 | 0.260 | 3.072 | 2.12E-03 | 0.019 | 2.999 | 0.986 | 3.040 | 2.37E-03 | 0.051 |
| ENSG00000178252 | WDR6 | 0.361 | 0.118 | 3.054 | 2.26E-03 | 0.020 | 0.298 | 0.142 | 2.089 | 0.037 | 0.243 |
| ENSG00000114200 | BCHE | -1.127 | 0.370 | -3.050 | 2.29E-03 | 0.020 | -2.669 | 0.955 | -2.795 | 5.19E-03 | 0.082 |
| ENSG00000181449 | SOX2 | -2.011 | 0.663 | -3.032 | 2.43E-03 | 0.021 | -4.701 | 0.911 | -5.159 | 2.49E-07 | 9.15E-05 |
| ENSG00000243955 | GSTA1 | -1.326 | 0.451 | -2.939 | 3.29E-03 | 0.026 | -4.596 | 0.980 | -4.691 | 2.72E-06 | 4.90E-04 |
| ENSG00000173826 | KCNH6 | -1.271 | 0.437 | -2.910 | 3.61E-03 | 0.027 | -2.995 | 0.823 | -3.638 | 2.74E-04 | 0.013 |
| ENSG00000133083 | DCLK1 | -0.685 | 0.236 | -2.909 | 3.62E-03 | 0.027 | -2.760 | 0.923 | -2.991 | 2.78E-03 | 0.057 |
| ENSG00000142920 | AZIN2 | 0.575 | 0.201 | 2.861 | 4.23E-03 | 0.030 | 1.281 | 0.462 | 2.770 | 5.60E-03 | 0.086 |
| ENSG00000174175 | SELP | -0.806 | 0.283 | -2.844 | 4.46E-03 | 0.032 | -2.604 | 1.007 | -2.586 | 0.010 | 0.118 |
| ENSG00000136261 | BZW2 | -0.292 | 0.103 | -2.841 | 4.50E-03 | 0.032 | -0.537 | 0.205 | -2.624 | 8.70E-03 | 0.110 |
| ENSG00000122420 | PTGFR | -0.706 | 0.248 | -2.840 | 4.51E-03 | 0.032 | -2.744 | 1.085 | -2.529 | 0.011 | 0.128 |
| ENSG00000257883 |  | -1.523 | 0.536 | -2.840 | 4.51E-03 | 0.032 | -2.846 | 1.131 | -2.517 | 0.012 | 0.131 |
| ENSG00000104332 | SFRP1 | -1.054 | 0.374 | -2.819 | 4.81E-03 | 0.033 | -3.740 | 1.017 | -3.679 | 2.34E-04 | 0.012 |
| ENSG00000172817 | CYP7B1 | -0.567 | 0.202 | -2.806 | 5.01E-03 | 0.034 | -4.170 | 1.269 | -3.284 | 1.02E-03 | 0.031 |
| ENSG00000183722 | LHFPL6 | -0.310 | 0.111 | -2.794 | 5.20E-03 | 0.035 | -3.337 | 0.794 | -4.204 | 2.62E-05 | 2.64E-03 |
| ENSG00000169710 | FASN | 0.457 | 0.168 | 2.712 | 6.68E-03 | 0.042 | 0.796 | 0.272 | 2.932 | 3.37E-03 | 0.063 |
| ENSG00000265399 |  | -0.588 | 0.219 | -2.692 | 7.10E-03 | 0.044 | -0.851 | 0.346 | -2.455 | 0.014 | 0.144 |
| ENSG00000237517 | DGCR5 | -1.017 | 0.379 | -2.681 | 7.34E-03 | 0.045 | -2.176 | 0.812 | -2.679 | 7.38E-03 | 0.100 |
| ENSG00000099949 | LZTR1 | 0.301 | 0.113 | 2.667 | 7.65E-03 | 0.046 | 0.523 | 0.225 | 2.327 | 0.020 | 0.175 |
| ENSG00000117114 | ADGRL2 | -0.409 | 0.154 | -2.663 | 7.74E-03 | 0.047 | -2.541 | 0.684 | -3.713 | 2.05E-04 | 0.011 |
| ENSG00000172828 | CES3 | 0.709 | 0.266 | 2.663 | 7.75E-03 | 0.047 | 1.370 | 0.655 | 2.091 | 0.037 | 0.243 |
| ENSG00000148120 | AOPEP | -0.370 | 0.139 | -2.656 | 7.90E-03 | 0.047 | -1.304 | 0.384 | -3.396 | 6.83E-04 | 0.023 |
| ENSG00000100429 | HDAC10 | 0.404 | 0.153 | 2.650 | 8.06E-03 | 0.048 | 0.579 | 0.291 | 1.990 | 0.047 | 0.275 |
| ENSG00000204335 | SP5 | -0.895 | 0.338 | -2.647 | 8.12E-03 | 0.048 | -2.936 | 0.753 | -3.898 | 9.71E-05 | 6.47E-03 |
| ENSG00000112195 | TREML2 | -1.131 | 0.427 | -2.647 | 8.12E-03 | 0.048 | -3.147 | 1.006 | -3.127 | 1.77E-03 | 0.042 |
| ENSG00000183287 | CCBE1 | -0.726 | 0.274 | -2.645 | 8.18E-03 | 0.048 | -2.792 | 0.831 | -3.359 | 7.82E-04 | 0.025 |
| ENSG00000237686 |  | 0.692 | 0.262 | 2.640 | 8.29E-03 | 0.049 | 1.865 | 0.664 | 2.810 | 4.96E-03 | 0.080 |
